# A MAGIC population-based genome-wide association study reveals functional association of *GhRBB1_A07* gene with superior fiber quality in cotton

**DOI:** 10.1186/s12864-016-3249-2

**Published:** 2016-11-09

**Authors:** Md Sariful Islam, Gregory N. Thyssen, Johnie N. Jenkins, Linghe Zeng, Christopher D. Delhom, Jack C. McCarty, Dewayne D. Deng, Doug J. Hinchliffe, Don C. Jones, David D. Fang

**Affiliations:** 1 Cotton Fiber Bioscience Research Unit, USDA-ARS, Southern Regional Research Center, New Orleans, LA 70124 USA; 2 Cotton Chemistry and Utilization Research Unit, USDA-ARS, Southern Regional Research Center, New Orleans, LA 70124 USA; 3 Genetics & Sustainable Agriculture Research Unit, USDA-ARS, Mississippi State, MS 39762 USA; 4 Crop Genetics Research Unit, USDA-ARS, Stoneville, MS 38772 USA; 5 Cotton Structure and Quality Research Unit, USDA-ARS, Southern Regional Research Center, New Orleans, LA 70124 USA; 6 Cotton Incorporated, Cary, NC 27513 USA

**Keywords:** Cotton, Fiber quality, Genome wide association study, Genotyping-by-sequencing, Multi parent advanced generation inter-cross

## Abstract

**Background:**

Cotton supplies a great majority of natural fiber for the global textile industry. The negative correlation between yield and fiber quality has hindered breeders’ ability to improve these traits simultaneously. A multi-parent advanced generation inter-cross (MAGIC) population developed through random-mating of multiple diverse parents has the ability to break this negative correlation. Genotyping-by-sequencing (GBS) is a method that can rapidly identify and genotype a large number of single nucleotide polymorphisms (SNP). Genotyping a MAGIC population using GBS technologies will enable us to identify marker-trait associations with high resolution.

**Results:**

An Upland cotton MAGIC population was developed through random-mating of 11 diverse cultivars for five generations. In this study, fiber quality data obtained from four environments and 6071 SNP markers generated via GBS and 223 microsatellite markers of 547 recombinant inbred lines (RILs) of the MAGIC population were used to conduct a genome wide association study (GWAS). By employing a mixed linear model, GWAS enabled us to identify markers significantly associated with fiber quantitative trait loci (QTL). We identified and validated one QTL cluster associated with four fiber quality traits [short fiber content (SFC), strength (STR), length (UHM) and uniformity (UI)] on chromosome A07. We further identified candidate genes related to fiber quality attributes in this region. Gene expression and amino acid substitution analysis suggested that a regeneration of bulb biogenesis 1 (*GhRBB1_A07*) gene is a candidate for superior fiber quality in Upland cotton. The DNA marker *CFBid0004* designed from an 18 bp deletion in the coding sequence of *GhRBB1_A07* in Acala Ultima is associated with the improved fiber quality in the MAGIC RILs and 105 additional commercial Upland cotton cultivars.

**Conclusion:**

Using GBS and a MAGIC population enabled more precise fiber QTL mapping in Upland cotton. The fiber QTL and associated markers identified in this study can be used to improve fiber quality through marker assisted selection or genomic selection in a cotton breeding program. Target manipulation of the *GhRBB1_A07* gene through biotechnology or gene editing may potentially improve cotton fiber quality.

**Electronic supplementary material:**

The online version of this article (doi:10.1186/s12864-016-3249-2) contains supplementary material, which is available to authorized users.

## Background

Cotton is one of the most important natural fibers for the textile industry and is a significant food source for livestock and human consumption. The industries associated with cotton fiber production and processing have a significant impact on the world economy [1, 2]. Although there are more than 50 species in the G*ossypium* genus, only four (*G. barbadense* L., *G. hirsutum* L., *G. arboreum* L. and *G. herbaceum* L.) are domesticated for cultivation [3]. Among these four cultivated species, Upland cotton (*G. hirsutum*) constitutes about 95 % of the world cotton production [2, 4]. Hence, most breeding efforts focus on the improvement of Upland cotton with the primary goal to improve yield and fiber quality. However, a simultaneous improvement of fiber quality and yield is challenging due to the presence of a negative genetic correlation between yield and quality [5]. Cotton breeders need to break this negative association in order to successfully breed improved cultivars.

In traditional QTL mapping, a bi-parental population is usually utilized to identify the genomic location and magnitude of effect of a locus that affects a phenotypic trait [5–7]. QTL mapping using such bi-parental populations is usually low in resolution since only two alleles per locus are analyzed and genetic recombination is limited [8]. A multi-parent advanced generation inter-cross (MAGIC) strategy has been anticipated to have higher genetic diversity, smaller haplotype blocks, higher recombination and better mapping resolution [8]. Thus, a MAGIC population in cotton has a better chance to break the negative linkage between yield and fiber quality [9]. Very recently, use of a MAGIC population to identify QTL has become a new approach, thanks to the advancement of next generation sequencing (NGS) techniques and novel statistical and bioinformatics tools to analyze large data sets.

Genome-wide association study (GWAS) falls into two categories: broad-based and narrow-based. Usually, a broad-based GWAS uses germplasm, landraces, cultivars, and natural populations to identify marker-trait associations. A narrow-based GWAS uses a man-made population involving more than two parents such as a MAGIC population to identify marker-trait associations. GWAS has several advantages over traditional QTL analysis, such as higher diversity, better utility of the identified QTL across diverse germplasm, and reduced time required for population development [10–12]. GWAS has been successfully employed in many plants, such as Arabidopsis [13], maize [10, 14], barley [15, 16], wheat [17, 18], rice [19], oat [20], sorghum [21] and soybean [11, 22]. In cotton, a few genetic studies using GWAS have been reported [2, 4, 23, 24]. Very recently another two GWAS papers in cotton have been published [25, 26]. All of those studies were based on using simple sequence repeat (SSR) markers.

With the advent of NGS technologies, GBS has already been proven to rapidly identify and genotype large numbers of single nucleotide polymorphism (SNP) markers in many crops [14] as well as in Upland cotton [27]. Although scientists have successfully identified marker-trait associations using GBS-based SNPs in many plant species [14, 21, 28, 29], so far no GWAS study has been reported in Upland cotton using SNPs. There are a few GWAS studies that were reported using SSR markers in Upland cotton [2, 4, 23, 24, 26, 30] as mentioned earlier. Among those reports, our laboratory previously identified 131 fiber QTL and 37 QTL clusters using 1582 SSR markers and 275 RILs of the same MAGIC population [4]. Recently, some candidate genes for fiber strength have been detected through gene expression and mapping-by-sequencing analysis [31–36]. So far, no report has been published to identify candidate genes in conjunction with GWAS analysis in cotton. In this study, a large number of GBS-based SNPs and a set of 223 SSRs were used to identify associations between fiber quality traits and DNA markers. We also used phenotypic data obtained from four environments for all RILs of the MAGIC population. In order to identify fiber quality candidate genes, we also investigated the most significant genomic location in more depth including whole genome sequencing (WGS) of the parental lines and gene expression analysis. We identified a positive correlation between the candidate gene regeneration of bulb biogenesis 1 (*GhRBB1_A07*) and fiber qualities in both the MAGIC RILs and a panel of 105 commercial cultivars. The marker-trait associations and candidate genes identified in this study may be useful to improve fiber quality in a cotton breeding program via marker-assisted selection (MAS) or genomic selection.

## Results

### Upland cotton MAGIC population structure assessment

**Fig. 1:**
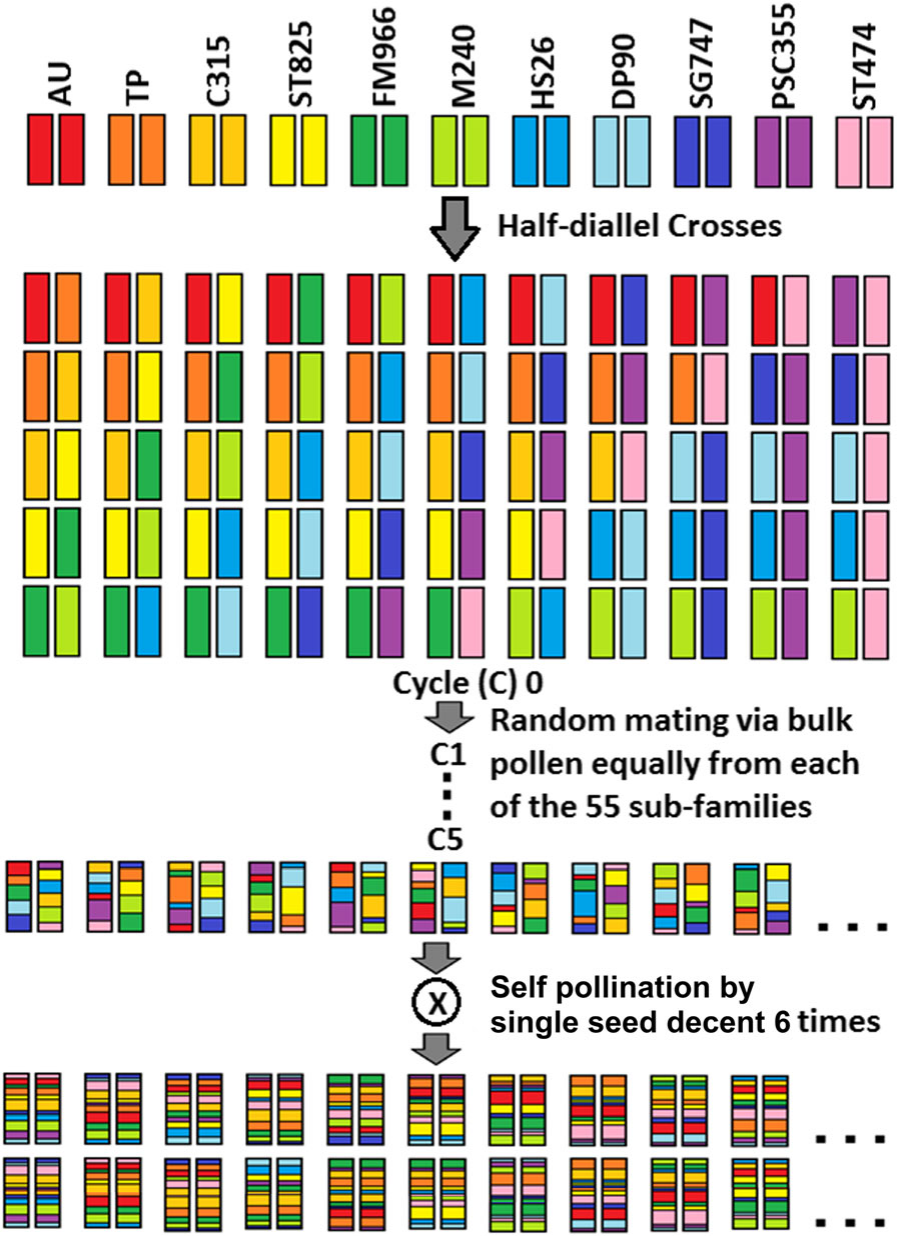
Breeding scheme of Upland cotton MAGIC population. Recombinant inbred lines (RILs) were developed according to the crossing scheme as below and 55 sub-families (10 shown here) were established. Individual colors represents the 11 parents (AU - Acala Ultima, TP - Tamcot Pyramid, C315 - Coker 315, ST825 - Stoneville 825, FM966 – Fibermax 966, M240 - M-240RNR, HS26 - Paymaster HS-26, DP90 - Deltapine Acala 90, SG747 – Suregrow 747, PSC355 – Phytogen 355, ST474 – Stoneville 474)

Ten cultivars and one non-commercial variety (Additional file 1) that represent broad Upland cotton diversity in the US were crossed in a half-diallel design to produce the MAGIC RIL population (C_5_S_6_) (Fig. 1 and Additional file 2) [9]. The effectiveness of random-mating and its consequence on structure of this population was evaluated using 6039 SNP and 223 SSR markers. The relatedness between the 547 RILs is presented as a heat map (Additional file 3). The relatedness matrix value of RIL pairs ranged from 0.253 to 0.637 which indicated that no specific population structure exists among the RILs. Kinship analysis also showed no specific clustering pattern of the RILs (Additional file 4). These results confirmed our previous conclusion that no obvious population structure was observed in this population [4].

### Fiber quality analysis

Fiber quality data of 11 parents and RILs were collected from 3 years (2009, 2010 and 2011) in Starkville, MS and 1 year (2013) in Stoneville, MS. In this study, six fiber quality traits were measured and results are shown in Table 1. Parents’ trait mean and RILs’ mean were similar. However, ranges of all the traits of RILs were broader possibly due to transgressive segregation. The correlation coefficient analysis between all the traits of parents and RILs were conducted separately. Results revealed positive strong correlation between strength (STR) and length (UHM), STR and uniformity (UI), and UHM and UI, in both parents and RILs fiber data (Table 2). We previously analyzed the heritability (*H* ^*2*^ *)* of fiber quality traits of this MAGIC population. Their heritability was moderate to high [4]. Analyses of variance for data collected in all environments indicated highly significant genotypic effects for all tested fiber quality traits (Additional file 5). The environment (location year) effects were significant for combined data for each trait under field conditions. RIL environment interactions under field conditions were also significant suggesting that there were differences in the response of some genotypes to environment over the location years of these experiments.

**Table 1:** Fiber quality measurements of parents and RILs collected in four environments

Trait	Parents					RILs				
	Mean	SE	SD	Min	Max	Mean	SE	SD	Min	Max
ELO (%)	6.10	0.131	1.23	3.75	8.90	6.03	0.029	1.56	2.80	9.93
MIC	4.63	0.047	0.44	3.17	5.55	4.67	0.011	0.46	3.29	6.25
SFC (%)	7.44	0.074	0.70	5.78	9.20	7.38	0.020	0.81	5.01	10.86
STR (g/tex)	30.68	0.250	2.34	25.80	36.8	30.59	0.056	2.29	24.13	40.97
UHM (mm)	28.19	0.152	1.27	24.64	30.99	28.19	0.051	1.78	22.61	34.04
UI (%)	83.68	0.112	1.06	81.25	85.78	83.76	0.030	1.22	79.20	88.13

*ELO* percent elongation of fibers before breaking, *MIC* a measurement of fiber fineness or maturity by an airflow instrument that measures the air permeability of a constant mass of cotton fibers compressed to a fixed volume, *SFC* short fiber content, calculated as the content (%) of fiber shorter than 12.7 mm, *STR* force required to break a bundle of fibers one tex unit in size, *UHM* upper half mean fiber length, the average length of the longer one-half of the fibers sampled, *UI* uniformity index, calculated as the (mean length/UHM) × 100, *SE* Standard error mean, *SD* standard deviation

**Table 2:** Pearson (r) correlations among fiber quality traits in RILs of MAGIC population

Trait	MIC	SFC	STR	UHM	UI
Parents					
ELO	0.34**	−0.36**	−0.14	0.13	0.30**
MIC		−0.24*	−0.05	−0.23*	0.16
SFC			−0.50**	−0.48**	−0.84**
STR				0.41**	0.48**
UHM					0.58**
RILs					
ELO	0.15**	−0.47**	−0.01	0.27**	0.43**
MIC		−0.07**	−0.14**	−0.39**	−0.04
SFC			−0.56**	−0.48**	−0.89**
STR				0.52**	0.61**
UHM					0.65**

* Significant at the *p* value <0.05. **Significant at the *p* value < 0.01

### Alignment and distribution of SNP markers in allotetraploid Upland cotton TM-1 draft genome

Three RILs had very poor sequencing data, and were discarded from the study. Thus, the sequence data of 547 MAGIC RILs were used to obtain SNPs. A total of 128,212 SNP contigs were called. Of them, 6071 polymorphic SNPs were identified after filtering using the criteria: missing rate ≤ 20 %, minor allele frequency (MAF) ≥ 5 %, number of genotypes ≥ 2 and MAF difference between average of parents and RILs ≤ 20 %. In the case of SSRs, a total of 223 were selected based on our previous study result, and 255 SSR loci were scored. A summary of SNPs in both parents and RILs is shown in Table 3. Of the 11 parents, DP90 had the highest rate of SNP heterozygosity. All 6071 GBS-based SNP contigs and 223 SSR clone sequences were aligned to the allotetraploid Upland cotton TM-1 draft genome [37]. A total of 6039 SNPs and all 223 SSRs produced a hit, and were able to be aligned to the TM-1 genome (Additional file 6). The 32 non-aligned SNPs are probably located in contigs that have not been assembled into chromosomes in the TM-1 draft genome. The marker distribution along each chromosome is shown in Additional file 7 as histograms generated by counting markers in each Mb interval.

**Table 3:** SNP summary of 11 parents and 547 RILs

Sample^a^	Genotype (%)				Reads (million)	bp (million)
	Homozygote (major allele)	Homozygote (minor allele)	Heterozygote	Missing		
AU	59.2	16.1	18.8	5.9	2.55	163.07
C315	61.4	12.8	20.5	5.3	2.21	141.30
DP90	63.4	11.7	20.9	4.0	2.48	158.58
FM966	60.6	16.9	17.7	4.8	2.63	168.27
HS26	60.5	17.3	18.2	4.1	2.64	169.22
M240	61.0	16.9	17.8	4.4	2.73	174.61
PSC355	62.6	13.2	19.4	4.8	2.40	153.40
PSC355	62.6	13.2	19.4	4.8	2.40	153.40
SG747	65.5	12.1	17.9	4.5	2.35	150.30
ST474	66.4	10.6	19.6	3.3	2.17	138.96
ST825	63.8	14.0	17.1	5.1	2.20	141.11
TP	58.9	17.9	17.8	5.4	2.51	160.70
RIL avg.	62.1	14.8	16.1	6.9	2.12	135.93

^a^ *AU* Acala Ultima, *C315* Coker 315, *DP90* Deltapine Acala 90, *FM966* Fibermax 966, *HS26* Paymaster HS-26, *M240* M-240RNR, *PSC355* Phytogen 355, *SG747* Suregrow 747, *ST474* Stoneville 474, *ST825* Stoneville 825, *TP* Tamcot Pyramid

### Linkage disequilibrium (LD)

**Fig. 2:**
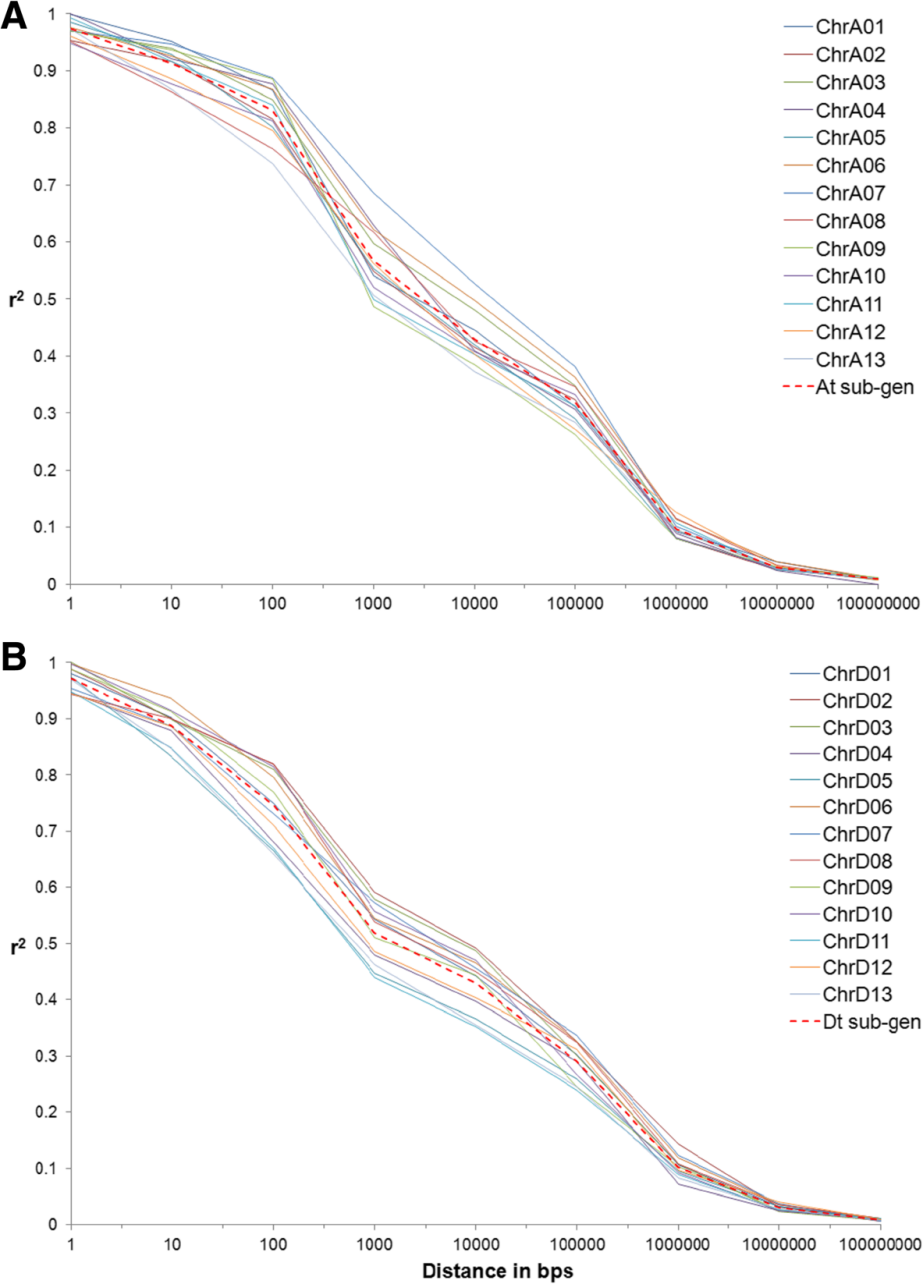
Linkage disequilibrium (LD) decay across all chromosomes. **a** A_t_ sub-genome, **b** D_t_ sub-genome

The square of correlation coefficient (r^2^) between markers located on each chromosome was measured to create a LD relationship between loci by using TASSEL 5.0 software [38]. We prefer to use r^2^ values over normalized LD coefficient (D′) since r^2^ between two loci have more reliable sampling properties and are influenced by both mutation and recombination events in the population. The LD decay plots for each chromosome and sub-genome were created by plotting r^2^ value onto physical distance measured in base pairs (Fig. 2). As anticipated, the r^2^ value negatively correlated with the physical distance between the loci. Results revealed that LD decay varied between chromosomes. Both sub-genomes had similar physical distances of reaching the LD threshold (r^2^ = 0.2) with ~520 kb and ~480 kb for A_t_-subgenome and D_t_-subgenome, respectively. Among the chromosomes, the slowest and most rapid LD decay was observed in chromosome A07 and D11, respectively (Fig. 2). Chromosome-wide LD contour plots are shown in Additional file 8.

### Genome-wide association analysis of fiber quality traits

**Fig. 3:**
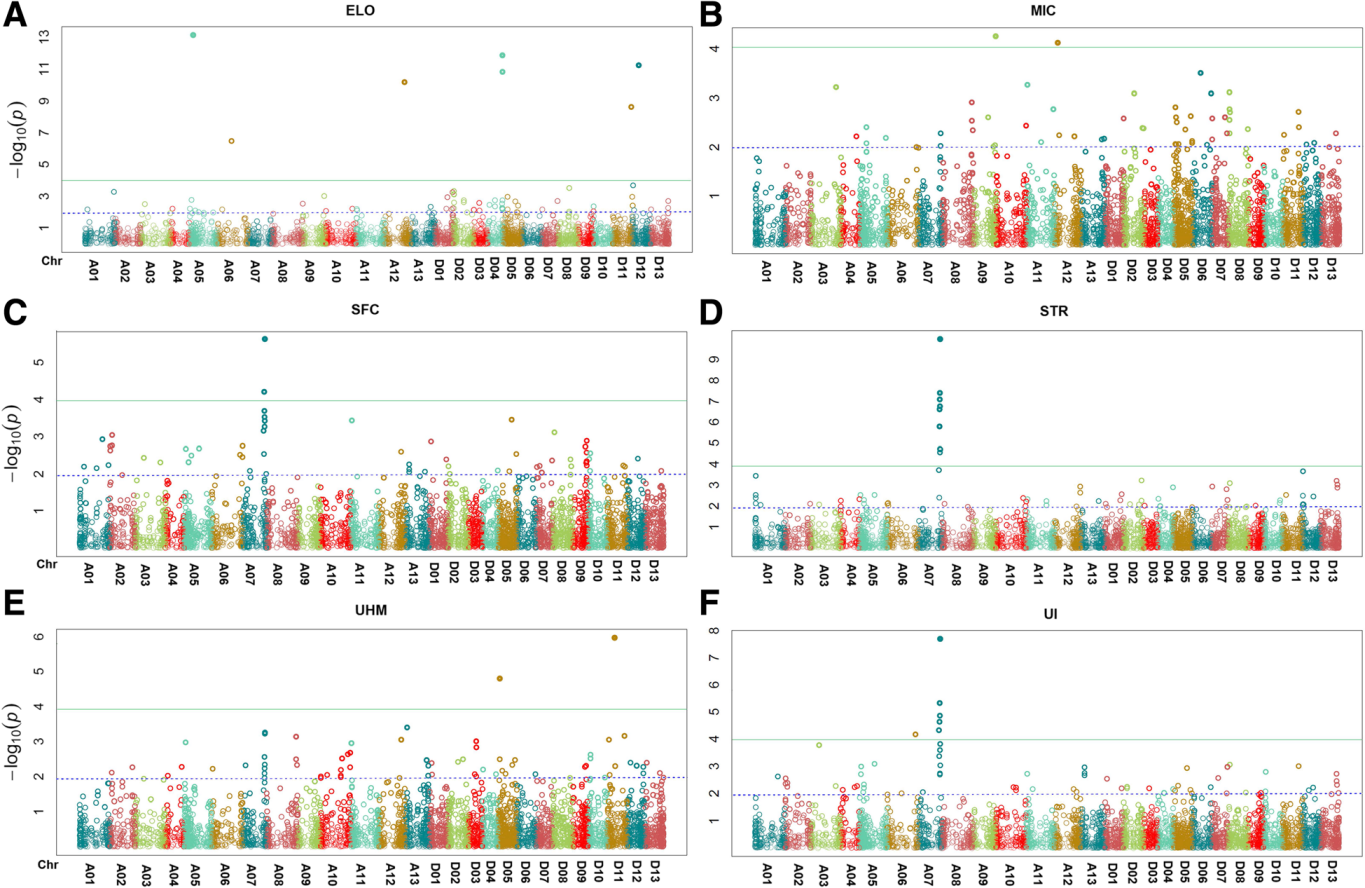
Manhattan plots generated from GAPIT software for six fiber quality traits, **a** Elongation (ELO), **b** Micronaire (MIC), **c** Short fiber content (SFC), **d** Fiber strength (STR), **e** Upper half mean (UHM) fiber length, and **f** Uniformity (UI). The negative log_10_ transformed *p* values were plotted against the marker positions on the physical map of each of the 26 Upland cotton chromosome. The significant thresholds (*p* = 0.01 and 0.0001) are indicated by the purple and green horizontal dot line, respectively

First, we performed GWAS using the best linear unbiased predictor (BLUPs) of RILs’ mean over four environments in a general linear model (GLM) with incorporation of principle component analysis (PCA) as Q matrix (population structure) by employing TASSEL 5.0 software [38]. The quantile-quantile (Q-Q) plots representing expected and observed probability of obtaining association of markers with respective traits revealed the possibility of false positive associations, since many of the observed *p*-values deviated from the uniform distribution (Additional file 9). Hence, a mixed linear model (MLM) [39] was used for further analysis in order to control the genomic inflation effectively using both TASSEL 5.0 [38] and GAPIT [40] software. This time, both PCA as Q and relatedness between RILs as K matrices were incorporated in the association analysis. Updated Q-Q plots showed that most of the observed *p*-values follow a uniform distribution but the few that are in LD with a causal polymorphism have significant *p*-values in the tail (Additional file 10). The Manhattan plots for six tested fiber quality traits were generated from GAPIT and TASSEL software and are presented in Fig. 3 and Additional file 11, respectively. Results from both programs were more or less identical, so we decided to proceed further with the results generated from GAPIT. At *p* value ≤ 0.01, the MLM identified 357 unique markers associated with 86 fiber QTL on 24 chromosomes (Table 4).

**Table 4:** Marker-trait associations and QTL identified at different significance level

Trait	Marker trait association				QTL^b^				
	*p* ≤ 0.01	*p* ≤ 0.001	*p* ≤ 0.0001	Total	*p* ≤ 0.01	*p* ≤ 0.001	*p* ≤ 0.0001	Total	# Chr.
ELO	54	8	7	69	13	1	1	15	10
MIC	58	6	2	66	16	0	0	16	14
SFC	54	9	2	65	10	0	1	11	10
STR	55	7	9	71	11	1	1	13	12
UHM	62	9	2	73	15	2	0	17	12
UI	58	8	6	72	13	0	1	14	13
Total	294^a^	41^a^	22^a^	357^a^	78	4	4	86	24^a^

^a^Some marker loci were associated with more than one trait

^b^Marker loci mapped within 5 Mb intervals were considered as a unique QTL

**Fig. 4:**
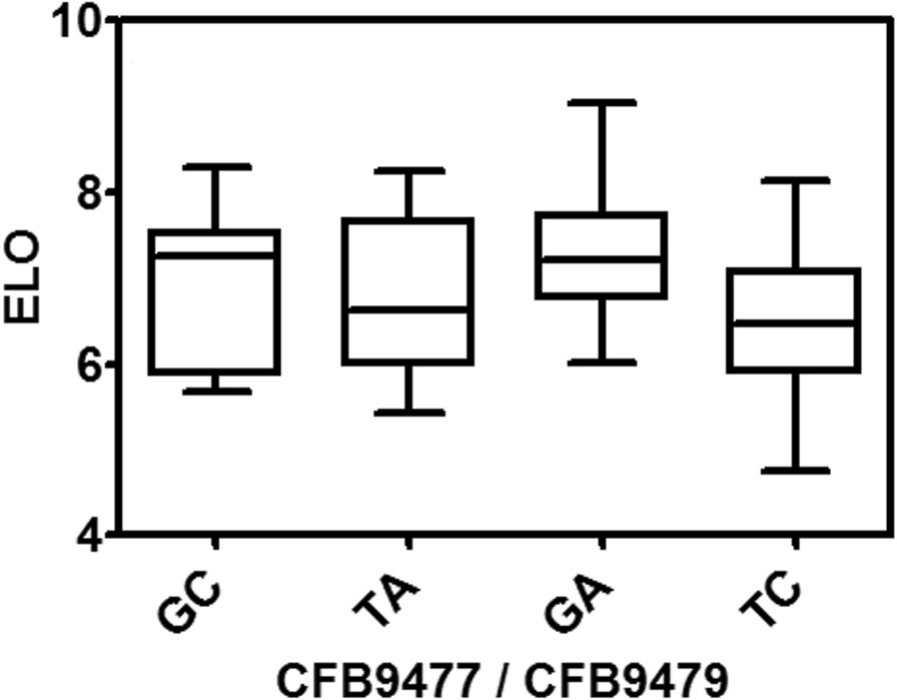
The effect of two marker loci selection on fiber elongation of RILs. RILs were divided into four groups based on the allele combinations at two marker loci that flanked a significant ELO QTL

The extremely significant (*p* value ≤ 0.0001) loci (*qELO-cD04*) associated with fiber elongation (ELO) comprises two SNPs (*CFB9477* and *CFB9479*) covering 152 Kb on chromosome D04 (Table 5, Additional file 12). The SNP (*CFB9477*) with strongest association with ELO (*p* value = 1.38E-12) is located at position 47,719,839 bp and explains 31 % of the phenotypic variation. Genotypes carrying the minor alleles for both the flanking SNPs (*GA*) at this QTL location had substantially higher ELO value than those carrying the flanking major alleles (*TC*) (Fig. 4).

**Table 5:** The extremely significant (*p* ≤ 0.0001) associations between markers and phenotypes

Trait	Marker	Chr.	Position (bp)	*p* value	MAF	R^2^	Allelic effect
ELO	MGHES-021	A05	12471766	7.48E-14	0.49	0.32	0.306
	MUSS275	A06	47495244	3.28E-07	0.50	0.28	0.196
	DPL0644c	A12	66210831	7.02E-11	0.40	0.30	−0.279
	CFB9477	D04	47719839	1.38E-12	0.30	0.31	−0.313
	CFB9479	D04	47872771	1.58E-11	0.32	0.31	−0.305
	SHIN-0966	D11	61712633	2.30E-09	0.49	0.29	0.223
	MUSB0846	D12	20174365	5.74E-12	0.49	0.31	0.274
MIC	CFB7672	A09	73256219	5.53E-05	0.21	0.11	−0.089
	CFB8181	A12	9895997	7.48E-05	0.50	0.11	−0.138
SFC	CFB7265	A07	70370764	6.22E-05	0.12	0.07	−0.266
	C2-0114	A07	72331925	2.32E-06	0.14	0.08	−0.240
STR	CFB7265	A07	70370764	2.41E-07	0.12	0.15	0.897
	DPL0852	A07	71015749	8.34E-08	0.18	0.15	0.689
	CFB7266	A07	71506333	4.17E-08	0.23	0.16	−0.701
	CFB7267	A07	71510617	1.90E-05	0.18	0.14	0.572
	CFB7268	A07	71510635	2.77E-05	0.19	0.14	−0.559
	DPL0757	A07	71588662	1.73E-07	0.17	0.15	0.698
	CFB7269	A07	72135309	1.45E-06	0.19	0.14	−0.619
	C2-0114	A07	72331925	1.07E-10	0.14	0.18	0.868
	CFB7271	A07	72856692	1.98E-05	0.13	0.14	0.625
UHM	CFB9550	D05	5051755	1.57E-05	0.49	0.14	0.011
	CFB11211	D11	24009159	1.07E-06	0.10	0.15	0.018
UI	MUSS122	A06	93195954	6.92E-05	0.25	0.07	0.207
	SHIN-1138	A07	68260333	4.85E-05	0.19	0.07	−0.010
	CFB7265	A07	70370764	2.31E-05	0.12	0.08	0.384
	DPL0852	A07	71015749	4.66E-06	0.18	0.08	0.309
	DPL0757	A07	71588662	1.36E-05	0.17	0.08	0.304
	C2-0114	A07	72331925	2.01E-08	0.14	0.10	0.404

*Chr*. Chromosome, *MAF* Minor allele frequency (%)

**Fig. 5:**
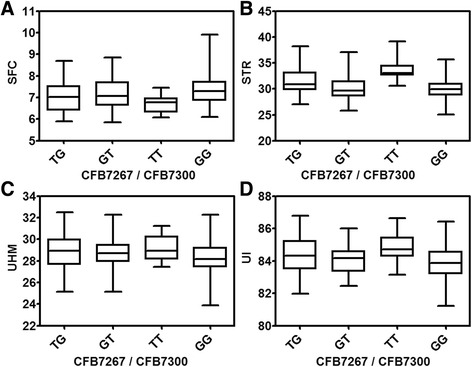
The effect of two marker loci selection on four fiber quality traits of RILs. **a** Short fiber content (SFC), **b** Fiber bundle strength (STR), **c** Fiber length (UHM), and **d** Uniformity (UI). RILs were divided into four groups based on the allele combinations at two marker loci that flanked a significant QTL of the respective trait

On chromosome A07, one of the most significant (*p* value ≤ 0.0001) loci (*qSTR-cA07*) associated with fiber strength had nine markers spanning 2.5 Mb. The same genomic region also housed QTL for SFC (*qSFC-cA07*) and UI (*qUI-cA07*) at *p* value ≤ 0.0001; and UHM at *p* value ≤ 0.001. The most significant linked marker is SSR marker *C2-0114* located at position 72,331,925 bp which contributes 8, 18, 13, and 10 %, of phenotypic variation for SFC, STR, UHM and UI, respectively (Table 5). Since this genomic region between 70 to 76 Mb on chromosome A07 is significantly associated with four fiber quality traits, we further investigated the annotated genes located in this region. In order to first find the effective border of the QTL region, we analyzed all the available haplotype combinations affecting the STR phenotype. Results showed that RILs carrying the minor alleles (*TT*) of SNPs *CFB7267* and *CFB7300* located between 71,510,617 and 76,885,949 bp produced significantly improved fiber strength than RILs carrying major alleles (*GG*) of those two SNPs (Fig. 5b). RILs carrying both major and minor alleles of markers located up and down stream of this border did not differ significantly (Additional file 13). The same result was also true for SFC, UHM and UI (Fig. 5a, c, d).

### Genomic architecture in the region flanked by SNP markers CFB7267 and CFB7300

The LD contour plot indicated that there are three large LD blocks present with a few small LD blocks in the genomic region between *CFB7267* and *CFB7300* (Additional file 14). Gene annotation of the Upland cotton TM-1 genome suggested 303 genes in this region. Based on the gene ontology, many of the genes are predicted to be transcription factors involved in cotton fiber development such as *CONSTANT-like 9* (*Gh_A07G1753*) and *oxydoreductase zinc-binding dehydogenase family protein* (*Gh_A07G1803*). Of the 303 genes, 142 had at least 1 read Per kilobase of transcript per million mapped reads (RPKM) expression in RNA-seq data from developing fiber cells of four Upland cotton cultivars and three cotton fiber mutant lines (Additional file 15).

Out of 142 genes, 18 genes, which were previously reported as candidates for fiber cell and/or cell wall development were selected for RT-qPCR gene expression analysis. In this experiment, we only selected two varieties Acala Ultima (AU, superior fiber quality) and Tamcot Pyramid (TP, inferior fiber quality) for comparative gene expression analysis through RT-qPCR. Of the 18 tested genes, the expression level of five genes (*Gh_A07G1753*, *Gh_A07G1758*, *Gh_A07G1784*, *Gh_A07G1795*, *Gh_A07G1803*) were significantly higher in AU developing fibers while one (*Gh_A07G1802*) showed down regulated expression in AU developing fibers compared with TP (Additional file 16).

### Identification of non-synonymous mutations near the *CFB7267*/*CFB7300* interval

**Fig. 6:**
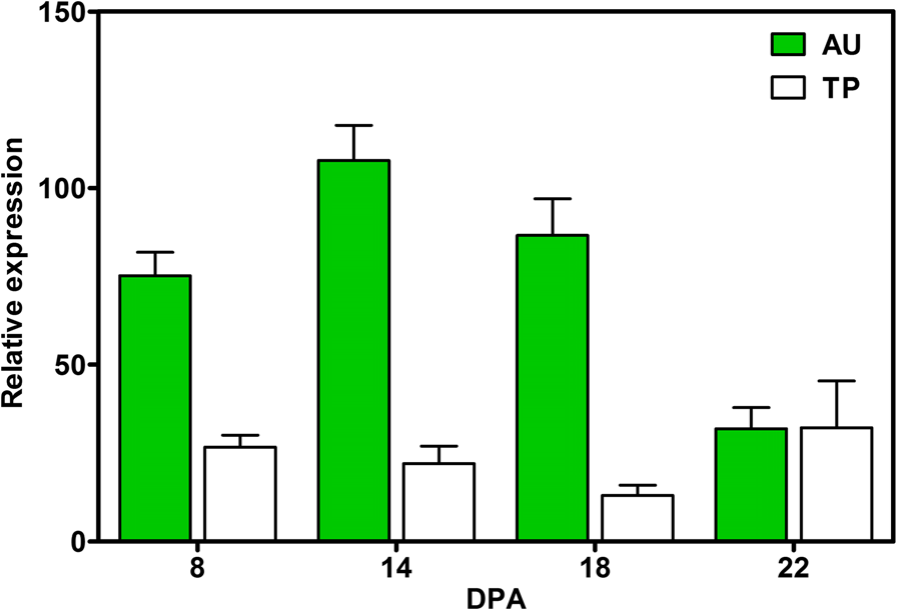
RT-qPCR gene expression analyses of gene *Gh_A07G2049* (*GhRBB1_A07*) during fiber development between Acala Ultima (AU) and Tamcot Pyramid (TP)

To identify mutations in the vicinity of the QTL cluster on chromosome A07 that were not represented in the GBS data, we analyzed whole genome sequence data from each of the 11 parents. By comparing AU with other parents, only 28 SNPs in nine genes produced mis-sense (27) and nonsense (1) substitutions of amino acid sequence (Table 6). The only nonsense amino acid mutation was observed in *Gh_A07G1913* (*Protein of unknown function, DUF593*). Since this gene was not well characterized and did not express in any of the developing fiber RNA-seq data (Additional file 15), we did not pursue further investigation. To investigate which mis-sense amino acid substitutions might have significant effect on fiber quality development, we compared the chemical nature of the substituted to the original amino acids and searched the Genebank for orthologous proteins with identical mutations. We found that 11 mis-sense mutations were conversions to chemically similar amino acids, while another 16 were synonymous to orthologous proteins (Table 6). However, one interesting mutation was detected in gene *Gh_A07G2049* (*Regeneration of bulb biogenesis 1, GhRBB1_A07*) at genomic location 76,911,659 bp which has an 18 bp deletion in the coding sequence in AU (Additional files 17 and 18). In order to find its possible effect on fiber development, we conducted RT-qPCR gene expression analysis using four [8, 14, 18 and 22 days post anthesis (DPA)] developmental stage fiber tissues of two parental lines AU and TP. The expression level of the gene was significantly higher in AU developing fibers than in TP (Fig. 6).

**Table 6:** Amino acid change caused by the SNP between Acala Ultima (AU) and others based on Upland cotton TM-1draft genome

Gene	Position (bp)	Amino acid code		TAIR10 gene	Description
		AU	Others		
Gh_A07G1913	74778174	L	Stop	AT2G30690	Protein of unknown function, DUF593
Gh_A07G2012	76384997	V	L	AT3G01480	Cyclophilin 38
Gh_A07G2019	76483636	E	Q	AT3G16520	UDP-glucosyl transferase 88A1
	76483537	L	F		
	76483477	S	A		
	76483123	C	F		
Gh_A07G2025	76582935	H	R	AT3G01510	Like SEX4 1
Gh_A07G2041	76841398	G	S	AT5G14570	High affinity nitrate transporter 2.7
Gh_A07G2042	76843818	H	R	AT1G50170	Sirohydrochlorin ferrochelatase B
Gh_A07G2045	76868459	L	F	AT3G01570	Oleosin family protein
Gh_A07G2046	76871281	T	A	AT3G27640	Transducin/WD40 repeat-like superfamily protein
Gh_A07G2049	76921226	Q	R	AT5G40450	Regulation of bulb biogenesis, *GhRBB1_A07*
	76917593	K	E		
	76917404	G	E		
	76914909	C	Y		
	76914822	Q	H		
	76914298	I	T		
	76911876	K	N		
	76911659	InDel	-		
	76910857	T	I		
	76909194	L	P		
	76908681	D	V		
	76907407	Q	E		
	76906421	N	S		
	76904993	D	N		
	76904590	A	T		
	76901416	K	E		
	76900228	F	L		
	76899907	S	C

**Fig. 7:**
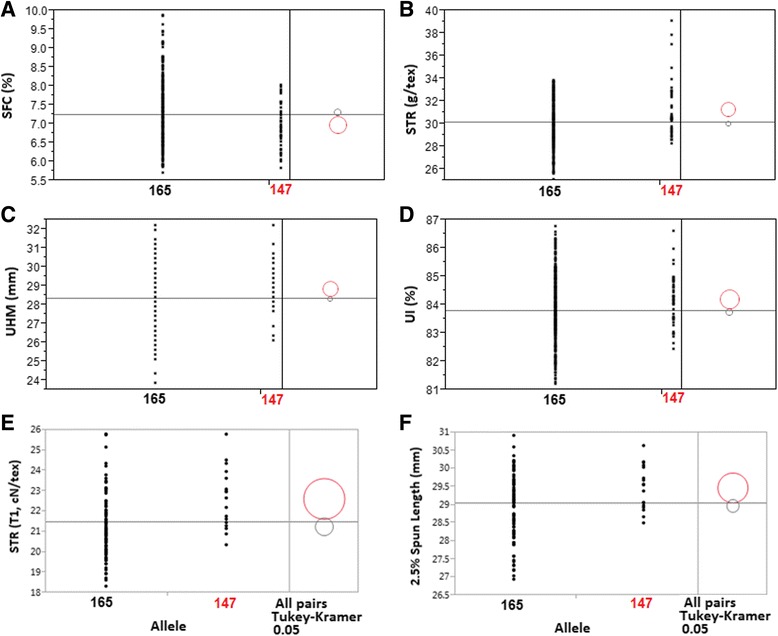
The correlation between the InDel marker CFBid0004 and fiber quality traits in MAGIC RILs and NCVT cultivars. Based on the marker genotypes, all 550 RILs of MAGIC population (**a**-**d**) and 105 Upland cotton cultivars (**e** and **f**) were divided into two groups: 1) RILs or cultivars carrying 147 bp allele and 2) the remaining RILs or cultivars containing 165 bp. The significant mean fiber traits of the genotype having 147 and 165 bp allele were shown in red and black circle, respectively

In order to explore the practical utility of this deletion, we designed an InDel marker (*CFBid0004*) (Forward primer, 5′ –TCTTTGATGACAACAACATTATAGA-3′ and reverse primer, 5′-AGAAACAGAAGAAACAGACATAAA-3′) and genotyped all 550 MAGIC RILs and 105 Upland cotton cultivars (Additional file 19) selected from the United States National Cotton Variety Test (NCVT) program. The InDel marker *CFBid0004* locus has two alleles: 147 bp and 165 bp. Two alleles of the locus were grouped for One-way analyses. For comparison of the means of the grouping performance, Tukey’s honest significant difference test was conducted for all pairs of levels. RILs carrying the 147 bp allele had a mean STR of 31.29 g/tex which was significantly higher than the mean STR (30.02 g/tex) of RILs carrying the reference allele 165 bp (Fig. 7b). Similarly, UHM and UI were also significantly increased while SFC was significantly decreased due to the presence of the deletion allele in the RILs genotype (Fig. 7a, c, d). The most remarkable results were found with historic phenotype data from NCVT trials. Upland cotton cultivars carrying the deletion allele (147 bp) had mean fiber strength and length of 29.46 cN/tex and 22.61 mm, which are significantly higher than mean fiber bundle strength and length (28.97 cN/tex and 21.24 mm), respectively, of cultivars carrying reference 165 bp allele (Fig. 7e, f).

## Discussion

### A MAGIC population is excellent for GWAS

Existence of various structures in a population can create spurious associations between markers and traits in a GWAS if the structure issue is not properly understood [41]. By combining high genetic diversity and low population structure, a MAGIC population creates positive characteristics for QTL study through association mapping [42]. By completing five random matings and six self-pollinations, we effectively reduced the population structure yet maintained relatively high allelic diversity within the MAGIC population. Both Kinship and relationship matrix revealed that there is essentially no particular population structure in the MAGIC population (Additional files 3 and 4). We previously reported similar result based on 1582 SSR markers [4]. Our results are also comparable with the genetic studies using MAGIC populations developed in other crops such as maize [43], rice [28] and wheat [44]. Thus, we believe that our Upland cotton MAGIC population is an excellent resource for GWAS as well as other genetic studies.

### Linkage disequilibrium in the Upland cotton MAGIC population

One of the major factors for GWAS analysis is the LD between loci. The minimum number of markers that are required to conduct a successful association analysis depends on the extent of LD over physical distance in a given population. Many factors affect LD such as recombination and mutation rate, relatedness (kinship), gene conversion, selection (natural, artificial, and balancing), mating systems (self or cross pollination), genetic diversity, and population structure [29, 45]. In this study, the average LD decay was approximately 520 and 480 Kb in A_t_ and D_t_ sub-genome, respectively, and genome wide ~500 Kb (Fig. 2). The cotton genome is about 2.5 Gb. Thus, for this cotton MAGIC population, we expect that a minimum number of markers required for GWAS are about 5000, and we used 6326 SNP and SSR loci. This is the first study to use more than 6000 marker loci for a GWAS research in cotton to the best of our knowledge. There have been some reports on LD measurements in Upland cotton, however none of those studies were based on physical distance (bp) but instead relied on genetic distance (cM). Thus, it is hard to compare prior results with this study. If we convert genetic distance to physical distance at the rate of 1 cM = 681 Kb as reported by Hulse-Kemp et al. [46], then LD decay in this study is faster than the 5–6 cM [23], or 3 to 4 cM [30] previously reported. This faster LD decay might be due to the use of MAGIC population in this study. The five random-matings may have effectively broken possible linkages and consequently reduced LD. In this study, the LD blocks are more or less uniform across the genome except on a few chromosomes (Additional file 8). This uniformity may also be due to the lack of selection during population development, since human selection inflates LD [43].

### GWAS of fiber quality traits

GWAS is a forward genetics approach which has been used to identify underlying causal genes, mutations and putative functional markers that affect complex quantitative traits [11, 14]. In this study, we identified 86 fiber QTL at *p* ≤ 0.01. This number is lower than 131 we reported earlier using phenotypic data from two environments of 275 RILs analyzed by 1582 SSR markers [4]. In this study, we used 6326 marker loci with known physical locations, 547 RILs, and phenotypic data from 4 environments. Thus, the mapping accuracy is expected to be higher. After comparing the 86 QTL with the previously-identified 131 QTL, a great majority are congruent in genomic locations. The region encompassing a major fiber QTL and *GhRBB1_A07* gene was identified in both reports with very high confidence.

The 86 QTL detected in this study were distributed across 24 chromosomes (Table 4, Additional file 12). Chromosomes A02 and D05 did not have any QTL. With agreement with previous reports [47, 48], we identified 26.32 % more fiber QTL on D_t_ sub-genome than A_t_ sub-genome. Similarly, our previous study also reported 21 % more fiber QTL on D_t_ sub-genome when compared with A_t_ sub-genome using 275 RILs of the same MAGIC population [4]. Once again this emphasizes that the D_t_ sub-genome has greater impact in determining fiber quality in Upland cotton than the A_t_ sub-genome. QTL clusters are more interesting to cotton breeders since the markers flanking these regions can be used to select more than one trait through MAS. It is very common to identify QTL clusters in cotton [4, 47–49]. In the present study, we identified 16 QTL clusters ranging from two to five QTL in a cluster (Additional file 12).

The QTL cluster on chromosome A07 for STR, SFC, UHM and UI appeared to be particularly interesting and valuable for cotton breeding. From the allelic effect results, it is clear that this QTL cluster has a positive effect on fiber quality by increasing STR, UHM, and UI value while decreasing SFC value (Additional file 12). This QTL cluster region is co-localized with the QTL cluster identified on chromosome 07 in our previous report [4] and in other reports [7, 50, 51]. Cao et al. [7] suggested that the QTL for fiber length and strength might come from the introgression from *G. barbadense*. Because of the large effects of this QTL on multiple traits, it may serve as an excellent candidate for MAS to improve fiber quality in breeding. In order to prove the effectiveness of these loci in MAS, we examined the allelic effects of the flanking markers on the fiber phenotypes in the MAGIC RILs. The four traits (SFC, STR, UHM and UI) could be simultaneously improved by selecting the minor alleles of the two flanking SNPs *CFB7267* and *CFB7300* in the MAGIC population.

### Identification of candidate gene for superior fiber quality

We followed a novel strategy to predict candidate genes for superior fiber quality in Upland cotton by integrating several approaches along with GWAS results. At first, the list of genes in the genomic region on chromosome A07 (71 to 77 Mb) encompassing a major fiber QTL and their annotation information were extracted from the allotetraploid cotton TM-1 draft genome. The ability to identify candidate genes in MAGIC population QTL mapping was further improved by incorporating WGS and gene expression data of the founder lines. Moreover, our previous RNA-seq data from seven Upland cotton germplasm were used to identify the expressed fiber-related genes. Finally, RT-qPCR gene expression results confirmed expression of the candidate genes in developing fibers. Out of 18 possible candidate genes, six were differentially expressed in the superior fiber quality parent line (AU) as compared to an inferior parent line (TP) fiber tissue (Additional file 16).

By utilizing WGS data from the 11 founder lines, we were able to detect non-sense and mis-sense amino acid mutations as well as other kind of mutations among the founder lines. Of all the detected mutations, the 18 bp deletion at genomic location 76,911,659 bp on chromosome A07 in Acala Ultima was particularly interesting. This deletion is in the exon of the gene *Gh_A07G2049* (*Regeneration of bulb biogenesis 1, GhRBB1_A07*). Interestingly, *RBB1* corresponds to a very large protein of unknown function that is specific to plants, is present in the cytosol, and may associate with cellular membranes. This gene is involved in the regulation of vacuole morphology and may be involved in the establishment or stability of trans-vacuolar strands (TVS) and bulbs. TVS form predominantly along the root hair tip growing axis and are thought to deliver cytosolic components to the growing tip [52]. In this research, RT-qPCR results revealed that transcripts of *GhRBB1_A07* gene were highly abundant in developing fibers (8, 14 and 18 DPA) of the superior founder line AU as compared to the inferior founder line TP. Interestingly, only AU has the deletion, thus we hypothesize that the *GhRBB1_A07* allele in AU may transport higher amount of cytosolic liquid to developing fiber cells by increasing TVS in vacuoles which may lead to superior fiber quality. We also took advantage of the deletion in *Gh_A07G2049* by designing an InDel marker (*CFBid0004*) to validate its association with fiber quality by genotyping MAGIC RILs and NCVT cultivars. Results showed that RILs having the 147 bp allele had significantly more favorable SFC, STR, UHM, and UI than RILs with the 165 bp allele (Fig. 7). Furthermore, NCVT historic data independently confirmed the finding that the deletion allele has an impact on fiber quality in diverse Upland cotton germplasm. We believe that the *GhRBB1_A07* gene is involved in cotton fiber development and the InDel marker *CFBid0004* can be used in MAS to improve fiber quality in a cotton breeding program.

## Conclusions

Use of a MAGIC population with little structure coupled with a high density marker coverage obtained through GBS enabled us to more precisely describe LD over the whole genome and to conduct GWAS at high resolution in Upland cotton. By employing an appropriate statistical model, GWAS identified markers significantly associated with fiber QTL. We further confirmed one major QTL cluster associated with four fiber quality traits (SFC, STR, UHM and UI) on chromosome A07. The availability of the reference Upland cotton genome and gene annotation also facilitated the identification of candidate genes, leading to the development of a functional marker for MAS. We were able to identify candidate genes by integrating WGS, and gene expression data of the founder lines. Finally, RT-qPCR gene expression results confirmed fiber expression of candidate genes for superior fiber quality in Upland cotton. Gene expression and amino acid mutation analysis suggested the *GhRBB1_A07* gene is likely one of the candidates for superior fiber quality in Upland cotton. The InDel marker *CFBid0004* has been proven to be associated with improved fiber quality in the MAGIC RILs and NCVT cultivars. The identified QTL can potentially be used to breed cotton cultivars with higher fiber quality through MAS or genomic selection. Further work will investigate the specific role of *GhRBB1_A07* gene in cotton fiber development.

## Methods

### Upland cotton MAGIC population

A set of 10 cultivars and one breeding line (Additional file 1) from major breeding programs across the United States were used as parents of the MAGIC population. The breeding scheme of the MAGIC population used in this study is shown in Fig. 1. The details of the population development were previously described by [4, 9, 27]. In brief, a half-diallel crossing scheme between 11 parents were followed to produce 55 F_1_ in 2002. The 55 F_1_ were considered as 55 half-sib families and designated as Cycle 0 (C_0_). Five cycles (C_1_ to C_5_) of random mating were made by bulking the equal amount of pollen from each of the 55 families. After that, self-pollination was followed for six generations using single seed descent method to produce RILs. Ten RILs were randomly selected from each of the 55 families and used in this study (Additional file 2).

### Field experiments, fiber quality measurement and phenotypic data analysis

Five hundred fifty MAGIC RILs and 11 parents were planted in Starkville (2009, 2010, and 2011) and Stoneville (2013), Mississippi, USA. Entries were laid out in single row plots 12 m long with about 120 plants per plot with two replications per RIL at each location-year. Standard field practices were applied over the plant growing seasons across years and locations. Twenty-five naturally opened bolls were harvested manually from the central part of a plant from each RIL and parent in all the location-years. Cotton bolls were ginned by using a 10-saw laboratory gin. Fiber quality attributes were measured using a High Volume Instrument (HVI, USTER technologies Inc., Charlotte, NC) for the following traits: ELO (%), MIC, SFC (%), STR (g/tex), UHM (mm), and UI (%). Variance components were estimated using the following statistical model.

1 $$ {y}_{ijk}=\upmu + {\mathrm{G}}_{\mathrm{i}} + {\mathrm{E}}_{\mathrm{j}} + {\mathrm{G}\mathrm{E}}_{\mathrm{i}\mathrm{j}} + {\mathrm{R}}_{\mathrm{j}\left(\mathrm{k}\right)} + {\upvarepsilon}_{\mathrm{i}\mathrm{j}\mathrm{k}} $$

where y_ijk_ is an observed value, μ the overall mean, G_i_ the effect of the inbred line i, E_j_ the effect of location year j, GE_ij_ the interaction between inbred line i with the location year j, R_j(k)_ the effect of replication k within environment j, and Ɛ_ijk_ the residual. In this study, all effects except μ were considered as random to estimate variance components. Lines adjusted means were obtained from BLUP considering overall mean. Computations were performed by using PROC MIXED in SAS (SAS Institute, Cary, NC, USA). Simple Pearson correlation coefficients (r) were calculated among all traits based on the adjusted means of the 550 RILs and parents separately. Analyses of variance of fiber quality traits was conducted using SAS package as well.

### DNA extraction and genotyping

Seeds from the RILs and parents were sown in small pots in a greenhouse in 2013 in New Orleans, Louisiana, USA. Young leaves were collected from ten plants of each of the entries and bulked. Leaves were stored at −80 °C. Total DNA was extracted from the frozen leaves following a protocol described earlier [27]. DNA quantity and quality was measured using a Nanodrop 2000 spectrophotometer (Thermo Fisher Scientific, Waltham, MA) as well as on a 1.5 % agarose gel.

Genotyping was conducted using both SSR and GBS-based SNP markers. A total of 223 SSR markers were selected based on our previous report [4] and used for genotyping all the RILs along with parents. SSR genotyping method was described earlier [4]. All the SSR marker sequences were BLASTed against Upland cotton TM-1 draft genome [37] in order to obtain their physical locations.

For GBS, DNA of RILs along with parents were sent to Institute for Genomic Diversity (IGD, Cornell University, Ithaca, NY) for library preparation, sequencing, and subsequent bioinformatics. The detail protocol for library preparation and sequencing was described earlier [53]. The raw reads alignment and SNP calling was conducted using reference genome sequence of *G. arboreum* [54] for sub-genome A and *G. raimondii* [55] for sub-genome D. The SNP sequences were filtered according to the criteria: missing rate ≤ 20 %, allele number = 2, MAF ≥ 5 %, number of genotypes ≥ 2. Polymorphisms and MAF among the parents and RILs were checked separately for each of the filtered SNPs. The SNPs with > 20 % MAF difference between the means of parents and RILs were discarded. Finally all filtered SNP sequences were aligned against allotetraploid Upland cotton TM-1 draft genome [37]. The SNP nomenclature was created in our laboratory starting with CFB (cotton fiber bioscience), followed by a serial number.

### Linkage disequilibrium (LD) determination

The genome-wide LD between pairs of loci was performed by using the software TASSEL 5.0 [38]. The estimates of the LD were measured using the squared allele-frequency correlations (r^2^) for pairs of loci. The distance in base pairs that loci could be expected to be in LD or LD decay was computed by plotting r^2^ onto physical distance using the threshold r^2^ = 0.2 as a cutoff. All markers with less than 20 % missing data and a minor allele frequency ≥ 5 % were used to measure LD decay. After getting r^2^ values, data were summarized using R statistical software for each of the chromosomes individually as well as combining all chromosomes to test a sub-genome wide LD decay. LD contour plot were generated from JMP genomics 6.0.

### Genome-wide association study

All marker-trait associations were performed using TASSEL 5.0 [38] and GAPIT (Genome Association and Prediction Integrated Tool-R package) [40]. All polymorphic markers that met the filtering criteria were used for GWAS analysis. A PCA was conducted to assess population structure, and a kinship (K) matrix was calculated using the VanRaden method and the EMMA method to determine the familial relatedness between lines. At first, the GLM including the PCA were tested for analyzing GWAS using TASSEL 5.0 [38]. An MLM [39] was also used for performing GWAS by incorporating K matrix along with PCA employing the program GAPIT and TASSEL 5.0. Q-Q plots were created to evaluate how much a significant result was produced by the analysis than expected by chance. A significant marker-trait association was declared when *p* value was equal to or smaller than 0.01. We also categorized the significant level as follows: significant *p* ≤ 0.01, highly significant *p* ≤ 0.001 and extremely significant *p* ≤ 0.0001. When multiple loci were associated with a trait and were within a 5 Mb interval, they were considered as a single QTL. The QTL nomenclature was according to [56].

### RNA extraction and gene expression analysis by reverse transcription quantitative PCR (RT-qPCR)

The parental lines were grown in Stoneville, MS in 2015 for RNA extraction. Total RNA was extracted from the developing cotton fibers (8, 14, 18 and 22 DPA) using the Sigma Spectrum™ Plant Total RNA Kit (Sigma-Aldrich, St. Louis, MO) with DNaseI digestion according to the manufacturer’s protocol. The quality and quantity of total RNA was determined using a NanoDrop 2000 spectrophotometer (NanoDrop Technologies Inc., Wilmington, DE, USA) and an Agilent Bioanalyzer 2100 (Agilent Technologies Inc., Santa Clara, CA, USA). The experimental procedures and data analysis related to RT-qPCR were performed according to the minimum information for publication of quantitative real-time PCR experiments guidelines [57]. The detail descriptions of cDNA preparation, RT-qPCR and calculation were previously reported [31]. Three biological replications and two technical replications for each time-point were used for RT-qPCR. Primer sequences for tested genes are included in Additional file 20.

### DNA and RNA sequencing and analysis

The 11 parental lines were sequenced at 20 × coverage with 101-bp paired end using Illumina HiSeq 2000. Sequence reads were aligned to the draft TM-1 genome using GSNAP software [37, 58]. DNA sequence polymorphisms in the vicinity of the major QTL cluster on Chr. A07 were identified with samtools and bcftools software and by manual inspection of alignment files with IGV software [59, 60]. Variants were analyzed for non-synonymous substitutions to annotated proteins as before [32, 61]. RNA-seq data previously published by our group was consolidated to identify fiber-expressed genes in the vicinity of the QTL cluster [31, 62, 63].

### Analysis of the InDel marker in 105 cotton cultivars

The InDel marker (*CFBid0004*) was designed from the sequence of the gene *Gh_A07G2049* using NCBI Primer Blast tools (Additional file 18). Then this marker was used to genotype all the MAGIC RILs and 105 Upland cotton cultivars. These 105 cultivars (Additional file 19) were selected because they were tested in multiple locations and multiple years through the NCVT program, and fiber quality data are available at http://www.ars.usda.gov/main/docs.htm?docid=23813 [64]. The InDel marker genotyping method was according to [63]. Phenotypic data for two alleles of 165 and 147 bp of the locus are grouped for the one-way analyses. For comparison of the means of the grouping performance, Tukey’s honest significant difference test was conducted for all pairs of levels.

## Additional files

Additional file 1: Title: Eleven Upland cotton cultivars that were used for MAGIC population development. Description of data: This table contains names of 11 founder lines used to develop the MAGIC population. This table was taken from DD Fang, JN Jenkins, DD Deng, JC McCarty, P Li and J Wu [4]. (DOCX 13 kb)
Additional file 2: Title: MAGIC population RILs pedigree and their corresponding family information. Description of data: Five hundred fifty RIL names, their pedigree and family information were included in this table. (XLSX 78 kb)
Additional file 3: Title: A heat map showing the relationships between RILs. Marker data were used to measure the relationships among 547 RILs of the MAGIC population. The red diagonal represents perfect relationship of each RIL and the symmetric off-diagonal elements represent relationship measured [in this case identity by descent (IBD)] for pairs of lines. No cluster of related lines was found as no block of diagonal warmer color appeared. The dendrogram on the right shows the results of a cluster analysis on the IBD matrix. Description of data: The relationship between the RILs of MAGIC population derived from JMPGenomics 6.0 is included in this figure. (DOCX 111 kb)
Additional file 4: Title: Kinship matrix among the 547 RILs of Upland cotton MAGIC population using GBS based SNP and SSR marker. Description of data: This figure contains the information of Kinship relationship among the tested RILs of MAGIC population. (DOCX 400 kb)
Additional file 5: Title: Analyses of variance for fiber quality trait data from recombinant inbred lines (RILs) of the Upland cotton MAGIC population. Description of data: ANOVA analysis of fiber quality trait data in Starkville, MS. (DOCX 13 kb)
Additional file 6: Title: SNP and SSR markers and their physical location on TM-1 draft genome. Description of data: This figure contains 6039 GBS based SNP and 223 SSR markers name, associated allele and physical location on allotetraploid cotton TM-1 draft genome. (XLSX 343 kb)
Additional file 7: Title: Polymorphic SNP and SSR marker distribution across the TM-1 genome. The length of X axis for all chromosomes is based on the highest physical length chromosome for the respective sub-genome. Description of data: GBS based SNP and SSR markers distributions per 1 Mb across TM-1 draft genome are shown in this figure. (DOCX 49 kb)
Additional file 8: Title: Linkage disequilibrium (LD) contour plot by chromosome generated from JMP genomics 6.0. Description of data: The LD contour plots for all the 26 Upland cotton chromosomes are included in this figure. The LD contour plot were generated from the square of correlation coefficients (r^2^) between markers located on each chromosome at different physical distances (bp) using JMP genomics 6.0 software. The X and Y axis have the physical distance of two markers. The square of correlation coefficients (r^2^) presents as color code (blue to red – 1.000 to 0.000). (DOCX 1734 kb)
Additional file 9: Title: Quantile-quantile (Q-Q) plot of six fiber traits generated from GWAS analysis following general linear model (GLM) using TASSEL 5.0 software. A) Fiber elongation (ELO), B) Micronaire (MIC), C) Short fiber content (SFC), D) Fiber strength (STR), E) Upper half mean fiber length (UHM), and F) Uniformity index (UI). Description of data: Q-Q plots of six fiber traits generated from GWAS analysis following GLM are included in this figure. The X and Y axis have the expected and observed negative logarithm 10 of *p* value, respectively generated during GWAS analysis. (DOCX 538 kb)
Additional file 10: Title: Quantile-quantile (Q-Q) Plot of six fiber traits generated from GWAS analysis following mixed linear model (MLM) using GAPIT software. A) Fiber elongation (ELO), B) Micronaire (MIC), C) Short fiber content (SFC), D) Fiber strength (STR), E) Upper half mean fiber length (UHM), and F) Uniformity index (UI). Description of data: Q-Q plots of six fiber traits generated from GWAS analysis following MLM are included in this figure. The X and Y axis have the expected and observed negative logarithm 10 of *p* value, respectively generated during GWAS analysis. (DOCX 207 kb)
Additional file 11: Title: Manhattan plots generated from TASSEL 5.0 software for six fiber quality traits, A) Elongation (ELO), B) Micronaire (MIC), C) Short fiber content (SFC), D) Fiber strength (STR), E) Upper half mean (UHM) fiber length, and F) Uniformity (UI). The negative log_10_ transformed *p* values were plotted against the marker positions on the physical map of each of the 26 Upland cotton chromosome. The significant thresholds (*p* = 0.01 and 0.0001) are indicated by the purple and green horizontal dot line, respectively. Description of data: Manhattan plots generated from TASSEL 5.0 software for six fiber quality traits from GWAS analysis following MLM are included in this figure. The X and Y axis have chromosome name and observed negative logarithm 10 of *p* value, respectively. (DOCX 813 kb)
Additional file 12: Title: Significant associations between markers and fiber quality traits. Description of data: This table contains the information of significant QTL at *p* ≤ 0.01 associated with six fiber quality traits detected from GWAS analysis following MLM. The first column have QTL name for the respective fiber traits (first letter ‘q’ for QTL then trait name and finally chromosome number). The second column contains marker name followed by chromosome number, physical location (bp), *p* vale, minor allele frequency (MAF), R^2^ of model without SNP, R^2^ of model with SNP, and estimated allelic effect. (XLSX 38 kb)
Additional file 13: Title: Effect of haplotype grouping near the major QTL on chromosome A07 on fiber bundle strength (g/tex). RILs were divided into two groups (major and minor types) based on genotypes major and minor alleles of respective two SNPs. Description of data: This box plot shows the effect of major and minor allele combination of haplotype group near the QTL on chromosome A07 on fiber strength. The X axis has the fiber strength value (g/tex) and Y axis contain possible major and minor type haplotype groups and their physical locations. (DOCX 101 kb)
Additional file 14: Title: LD contour plot in the Upland cotton genomic region of 71 to 77 Mb on chromosome A07. LD contour was created from genotypic data of 547 RILs of Upland cotton MAGIC population using JMP genomics 6.0 software. X axis is physical distance in Mb and r^2^ (CorrCoeff2) between marker pair is shown in different color block as per legend. Description of data: LD contour plot of genomic region of 71 to 77 Mb on chromosome A07 is included in this figure. Red color represents the higher r^2^ value between two markers due to LD block. The square of correlation coefficients (r^2^) presents as color code (red to blue – 1.000 to 0.000). (DOCX 69 kb)
Additional file 15: Title: Annotated genes within 71 M bp and 76 M bp region of chromosome A07 and their RNA-seq data. Description of data: This table contains gene name and associated annotation near the detected loci on chromosome A07. The RNA-seq data from our previous studies for the gene are also included in this table. First column have the gene name followed by physical location, Arabidopsis ortholog gene, gene description and RPKM value of RNA-seq data for the different RNA libraries. (XLSX 75 kb)
Additional file 16: Title: RT-qPCR gene expression analyses of selected genes related to cell wall activity during fiber development between Acala Ultima (AU) and Tamcot Pyramid (TP). A) CONSTANS-like 9 (*Gh_A07G1753*); B) RAB GTPase homolog A5E (*Gh_A07G1758*); C) Unknown protein family, DUF538 (*Gh_A07G1784*); D) Auxin-responsive protein (*Gh_A07G1795*); E) Oxidoreductase, zinc-binding dehydrogenase family protein (*Gh_A07G1803*); F) O-Glycosyl hydrolases family protein (*Gh_A07G1802*). Description of data: This file contains the results of RT-qPCR analysis data performed on six genes that were used to compare relative expression levels between superior line AU and inferior line TP. Four (8, 14, 28 and 22 DPA) developing fiber samples were used for RT-qPCR analysis. RT-qPCR values were corrected to 18S gene for each sample. For each treatment group two qPCR measurements were taken for each of three biological replicates and then averaged. (DOCX 161 kb)
Additional file 17: Title: Sashimi plot of parental lines showing 18 bp of deletion on parent Acala Ultima (AU) at genomic region 76,911,659 to 76,911,676 bp on chromosome A07. Description of data: Sashimi plot of parental lines generated from Integrated Genome Viewer (IGV) software is included in this file. The parent AU (row number 1) has an 18 bp deletion at genomic region 76,911,659 to 76,911,676 bp on exon 29 of gene *Gh_A07G2049* on chromosome A07 while other parental lines don’t have that deletion. (DOCX 50 kb)
Additional file 18: Title: Alignment of Acala Ultima (AU) and TM-1 (Ref) alleles of *Gh_A07G2049*. Primer locations for CFBid0004 are highlighted yellow. Description of data: This file contains alignment of parental line AU (row number 1) and TM-1 (row number 2) alleles of gene *Gh_A07G2049*. The 18 bp deletion in the coding sequence in AU at 17,875 to 17,893 bp on gene has also been presented. The forward and reverse primer sequences for indel marker CFBid0004 has been highlighted as yellow at either side of the 18 bp deletion. (PDF 4439 kb)
Additional file 19: Title: The 105 Upland cotton cultivars used for finding the efficacy of deletion marker allele CFBid0004 in this study. Description of data: This table contains 105 NCVT cultivars name. The historic phenotypic data of those 105 cultivars were used to find out the practical efficacy of the InDel marker CFBid0004 in Upland cotton breeding. (XLSX 11 kb)
Additional file 20: Title: Sub-genome specific primer names and their sequences used in this study for RT-qPCR gene expression. Description of data: This file contains 19 RT-qPCR primer pair sequences that were used to analyze gene expression near the major QTL on chromosome A07. Corresponding gene name and descriptions are also included here. (XLSX 13 kb)

## Abbreviations

AU: Acala Ultima
BLUP: Best linear unbiased predictor
DPA: Days post anthesis
ELO: Elongation
GBS: Genotyping-by-sequencing
GLM: General linear model
GWAS: Genome wide association study
HVI: High volume instrument
LD: Linkage disequilibrium
MAF: Minor allele frequency
MAGIC: Multi parent advanced generation inter-cross
MAS: Marker assisted selection
MIC: Micronaire
MLM: Mixed linear model
NCVT: National Cotton Variety Test
QTL: Quantitative trait locus (Loci)
RBB1: Regeneration of bulb biogenesis 1
RIL: Recombinant inbreed line
RPKM: Reads per kilobase of transcript per million mapped reads
RT-qPCR: Reverse transcription quantitative polymerase chain reaction
SFC: Short fiber content
STR: Fiber strength
TP: Tamcot pyramid
TVS: Trans-vacuolar strands
UHM: Upper-half mean fiber length
UI: Uniformity index
WGS: Whole genome sequence
