# Supplementary material for: A MAGIC population-based genome-wide association study reveals functional association of GhRBB1_A07 gene with superior fiber quality in cotton
Source: BMC Genomics. 2016 Nov 9;17:903. doi: 10.1186/s12864-016-3249-2 (PMC5103610; doi:10.1186/s12864-016-3249-2)
Supplement: Additional file 1: — Title: Eleven Upland cotton cultivars that were used for MAGIC population development. Description of data: This table contains names of 11 founder lines used to develop the MAGIC population. This table was taken from DD Fang, JN Jenkins, DD Deng, JC McCarty, P Li and J Wu [4]. (DOCX 13 kb) [file 12864_2016_3249_MOESM1_ESM.docx]

Additional file 1. **Eleven Upland cotton cultivars that were used for MAGIC population development^†^**

| # | Cultivar | Abbreviation | Developer |
| --- | --- | --- | --- |
| 1 | Acala Ultima | AU | California Planting Cotton Seed Distributors (Shafter, CA) |
| 2 | Coker 315 | C315 | Coker Pedigreed Seed Co. (Hartsville, SC) |
| 3 | Deltapine Acala 90 | DP90 | Delta and Pine Land Co. (Scott, MS) |
| 4 | Fibermax 966 | FM966 | Bayer Crop Science (Lubbock, TX) |
| 5 | M240RNR | M240 | USDA-ARS (Mississippi State, MS) |
| 6 | Paymaster HS26 | HS26 | Paymaster Technologies, Inc. (Plainview, TX) |
| 7 | Phytogen PSC 355 | PSC355 | Phytogen Seeds (Indianapolis, IN) |
| 8 | Stoneville 474 | ST474 | Stoneville Pedigreed Seed Co. (Stoneville, MS) |
| 9 | Stoneville 825 | ST825 | Stoneville Pedigreed Seed Co. (Stoneville, MS) |
| 10 | Suregrow 747 | SG747 | Sure-Grow Co. (Centre, AL) |
| 11 | Tamcot Pyramid | TP | Texas A&M University (College Station, TX) |

^†^This table was taken from [Fang et al. (2014](#_ENREF_7))
