# Supplementary material for: A MAGIC population-based genome-wide association study reveals functional association of GhRBB1_A07 gene with superior fiber quality in cotton
Source: BMC Genomics. 2016 Nov 9;17:903. doi: 10.1186/s12864-016-3249-2 (PMC5103610; doi:10.1186/s12864-016-3249-2)

Additional File 3. **A heat map showing the relationships between RILs.** Marker data were used to measure the relationships among 547 RILs of the MAGIC population. The red diagonal represents perfect relationship of each RIL and the symmetric off-diagonal elements represent relationship measured [in this case identity by descent (IBD)] for pairs of lines. No cluster of related lines was found as no block of diagonal warmer color appeared. The dendrogram on the right shows the results of a cluster analysis on the IBD matrix.


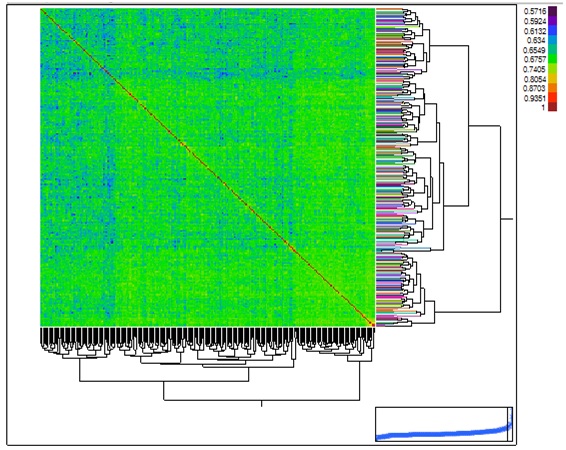

Supplement: Additional file 3: — Title: A heat map showing the relationships between RILs. Marker data were used to measure the relationships among 547 RILs of the MAGIC population. The red diagonal represents perfect relationship of each RIL and the symmetric off-diagonal elements represent relationship measured [in this case identity by descent (IBD)] for pairs of lines. No cluster of related lines was found as no block of diagonal warmer color appeared. The dendrogram on the right shows the results of a cluster analysis on the IBD matrix. Description of data: The relationship between the RILs of MAGIC population derived from JMPGenomics 6.0 is included in this figure. (DOCX 111 kb) [file 12864_2016_3249_MOESM3_ESM.docx]
