# Supplementary material for: A MAGIC population-based genome-wide association study reveals functional association of GhRBB1_A07 gene with superior fiber quality in cotton
Source: BMC Genomics. 2016 Nov 9;17:903. doi: 10.1186/s12864-016-3249-2 (PMC5103610; doi:10.1186/s12864-016-3249-2)
Supplement: Additional file 4: — Title: Kinship matrix among the 547 RILs of Upland cotton MAGIC population using GBS based SNP and SSR marker. Description of data: This figure contains the information of Kinship relationship among the tested RILs of MAGIC population. (DOCX 400 kb) [file 12864_2016_3249_MOESM4_ESM.docx]

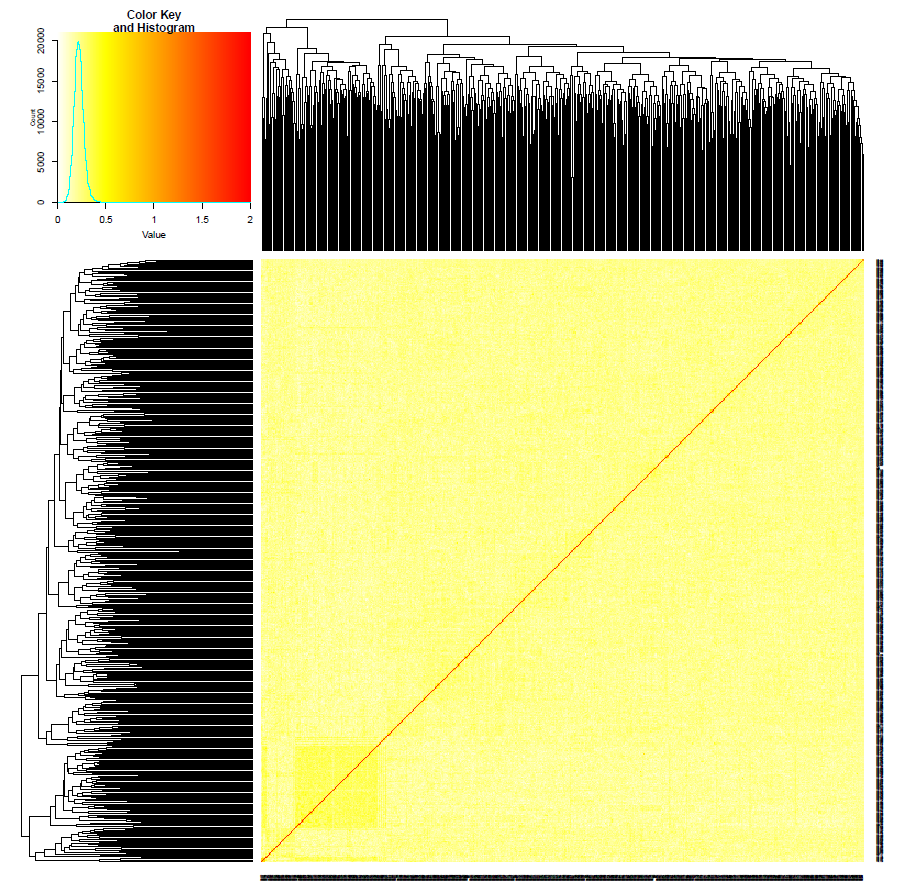
Additional file 4. **Kinship matrix among the 547 RILs of Upland cotton MAGIC population using GBS-based SNP and SSR marker.**
