# Supplementary material for: A MAGIC population-based genome-wide association study reveals functional association of GhRBB1_A07 gene with superior fiber quality in cotton
Source: BMC Genomics. 2016 Nov 9;17:903. doi: 10.1186/s12864-016-3249-2 (PMC5103610; doi:10.1186/s12864-016-3249-2)
Supplement: Additional file 5: — Title: Analyses of variance for fiber quality trait data from recombinant inbred lines (RILs) of the Upland cotton MAGIC population. Description of data: ANOVA analysis of fiber quality trait data in Starkville, MS. (DOCX 13 kb) [file 12864_2016_3249_MOESM5_ESM.docx]

Additional file 5: Analyses of variance for fiber quality trait data from recombinant inbred lines (RILs) of the Upland cotton MAGIC population.

| Source of variation | Degrees of freedom | ELO |  | MIC |  | SFC |  | STR |  | UHM |  | UI |  |
| --- | --- | --- | --- | --- | --- | --- | --- | --- | --- | --- | --- | --- | --- |
|  |  | Mean square | *p*-value | Mean square | *p*-value | Mean square | *p*-value | Mean square | *p*-value | Mean square | *p*-value | Mean square | *p*-value |
| Rep (LY) | 5 | 7.69 | 0.059 | 3.85 | 0.061 | 22.19 | 0.059 | 223.48 | 0.059 | 0.049 | 0.060 | 46.98 | 0.060 |
| LY | 2 | 1001.21 | <0.0001 | 70.02 | 0.005 | 323.86 | 0.008 | 1854.49 | 0.026 | 3.097 | 0.001 | 726.53 | 0.007 |
| RILs | 549 | 2.89 | <0.0001 | 1.08 | <0.0001 | 2.24 | <0.0001 | 29.40 | <0.0001 | 0.019 | <0.0001 | 5.11 | <0.0001 |
| RILs x LY | 1097 | 0.16 | <0.0001 | 0.11 | <0.0001 | 0.34 | <0.0001 | 2.92 | <0.0001 | 0.001 | <0.0001 | 0.85 | <0.0001 |
| Error | 2725 | 0.096 |  | 0.08 |  | 0.27 |  | 1.91 |  | 0.001 |  | 0.74 |  |

LY = Location year

ELO = percent elongation of fibers before breaking

MIC = a measurement of fiber fineness or maturity by an airflow instrument that measures the air permeability of a constant mass of cotton fibers compressed to a fixed volume

SFC = short fiber content, calculated as the content (%) of fiber shorter than 12.7 mm

STR = force required to break a bundle of fibers one tex unit in size

UHM = upper half mean fiber length, the average length of the longer one-half of the fibers sampled

UI = uniformity index, calculated as the (mean length/UHM) x 100
