# Supplementary material for: A MAGIC population-based genome-wide association study reveals functional association of GhRBB1_A07 gene with superior fiber quality in cotton
Source: BMC Genomics. 2016 Nov 9;17:903. doi: 10.1186/s12864-016-3249-2 (PMC5103610; doi:10.1186/s12864-016-3249-2)

Additional file 7. **Polymorphic SNP and SSR marker distribution across the TM-1 genome.** The length of X axis for the chromosomes is based on the highest physical length chromosome for the respective sub-genome.


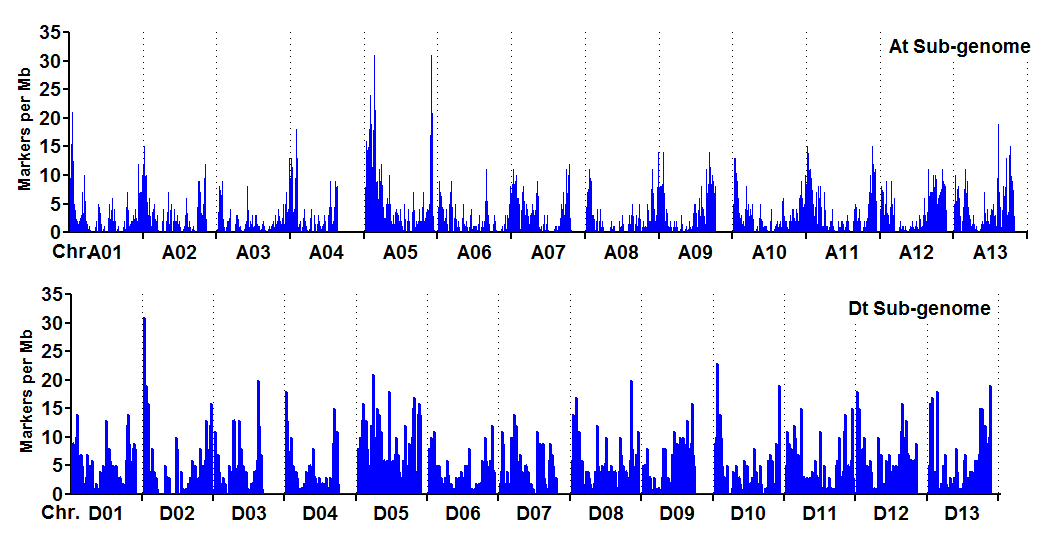

Supplement: Additional file 7: — Title: Polymorphic SNP and SSR marker distribution across the TM-1 genome. The length of X axis for all chromosomes is based on the highest physical length chromosome for the respective sub-genome. Description of data: GBS based SNP and SSR markers distributions per 1 Mb across TM-1 draft genome are shown in this figure. (DOCX 49 kb) [file 12864_2016_3249_MOESM7_ESM.docx]
