# Supplementary material for: A MAGIC population-based genome-wide association study reveals functional association of GhRBB1_A07 gene with superior fiber quality in cotton
Source: BMC Genomics. 2016 Nov 9;17:903. doi: 10.1186/s12864-016-3249-2 (PMC5103610; doi:10.1186/s12864-016-3249-2)

Additional file 8. **Linkage disequilibrium (LD) contour plot by chromosome generated from JMP genomics.**


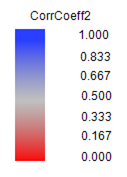
Chromosome A01

Chromosome A02

Chromosome A03

Chromosome A04

Chromosome A05

Chromosome A06

Chromosome A07

Chromosome A08

Chromosome A09

Chromosome A10

Chromosome A11

Chromosome A12

Chromosome A13


Chromosome D01

Chromosome D02

Chromosome D03

Chromosome D04

Chromosome D05

Chromosome D06

Chromosome D07

Chromosome D08

Chromosome D09

Chromosome D10

Chromosome D11

Chromosome D12

Chromosome D13


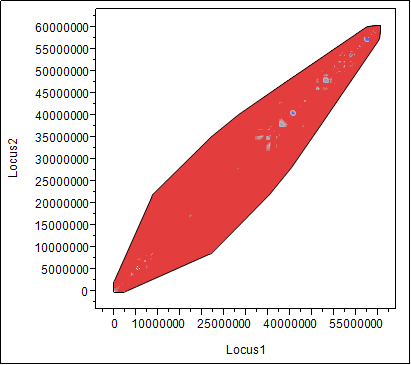

Supplement: Additional file 8: — Title: Linkage disequilibrium (LD) contour plot by chromosome generated from JMP genomics 6.0. Description of data: The LD contour plots for all the 26 Upland cotton chromosomes are included in this figure. The LD contour plot were generated from the square of correlation coefficients (r2) between markers located on each chromosome at different physical distances (bp) using JMP genomics 6.0 software. The X and Y axis have the physical distance of two markers. The square of correlation coefficients (r2) presents as color code (blue to red – 1.000 to 0.000). (DOCX 1734 kb) [file 12864_2016_3249_MOESM8_ESM.docx]
