# Supplementary material for: A MAGIC population-based genome-wide association study reveals functional association of GhRBB1_A07 gene with superior fiber quality in cotton
Source: BMC Genomics. 2016 Nov 9;17:903. doi: 10.1186/s12864-016-3249-2 (PMC5103610; doi:10.1186/s12864-016-3249-2)
Supplement: Additional file 10: — Title: Quantile-quantile (Q-Q) Plot of six fiber traits generated from GWAS analysis following mixed linear model (MLM) using GAPIT software. A) Fiber elongation (ELO), B) Micronaire (MIC), C) Short fiber content (SFC), D) Fiber strength (STR), E) Upper half mean fiber length (UHM), and F) Uniformity index (UI). Description of data: Q-Q plots of six fiber traits generated from GWAS analysis following MLM are included in this figure. The X and Y axis have the expected and observed negative logarithm 10 of p value, respectively generated during GWAS analysis. (DOCX 207 kb) [file 12864_2016_3249_MOESM10_ESM.docx]

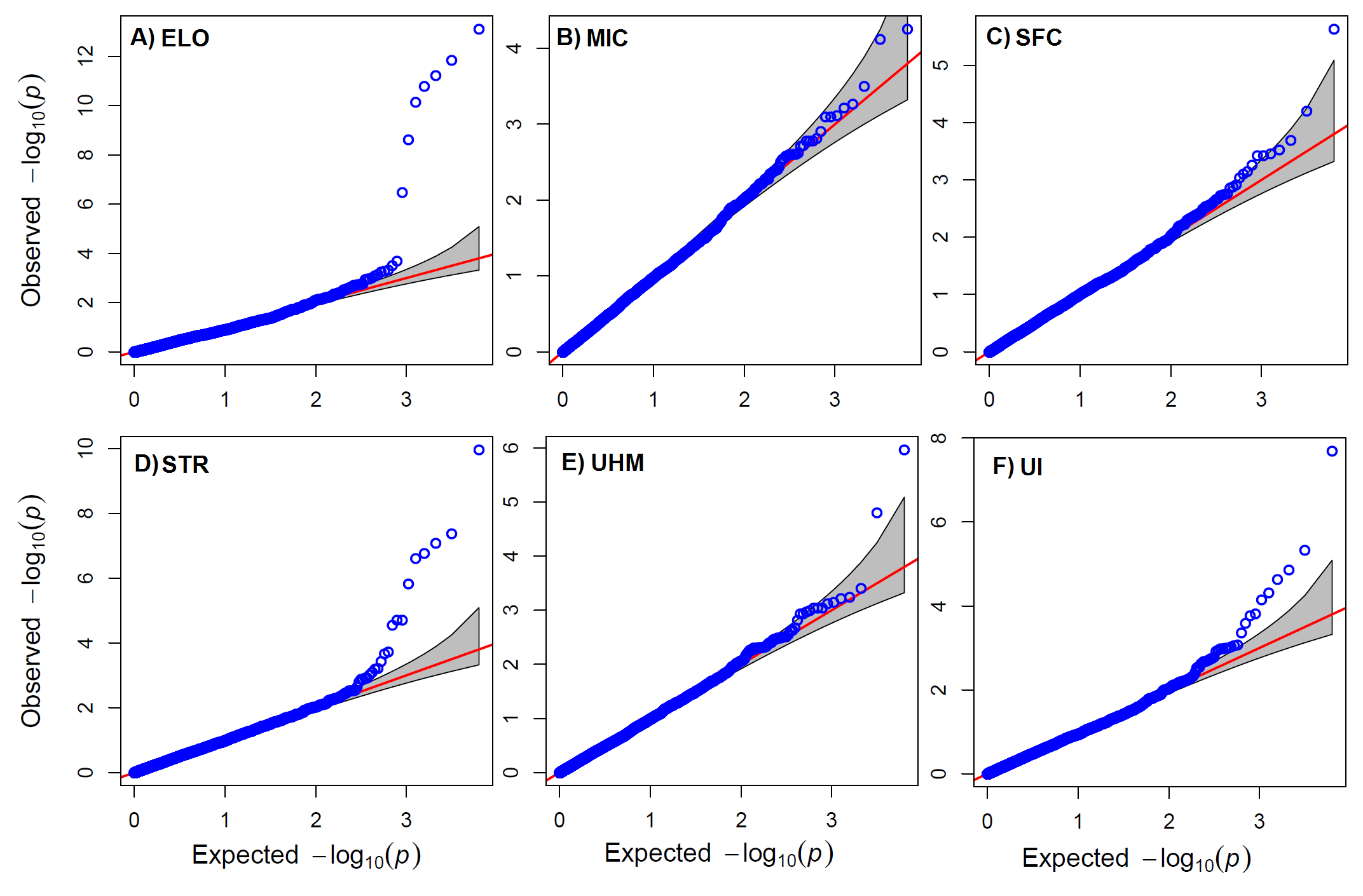
Additional file 10. **Quantile-quantile (Q-Q) Plot of six fiber traits generated from GWAS analysis following mixed linear model (MLM) using GAPIT software.** A) Fiber elongation (ELO), B) Micronaire (MIC), C) Short fiber content (SFC), D) Fiber strength (STR), E) Upper half mean fiber length (UHM), and F) Uniformity index (UI).
