# Supplementary material for: A MAGIC population-based genome-wide association study reveals functional association of GhRBB1_A07 gene with superior fiber quality in cotton
Source: BMC Genomics. 2016 Nov 9;17:903. doi: 10.1186/s12864-016-3249-2 (PMC5103610; doi:10.1186/s12864-016-3249-2)
Supplement: Additional file 11: — Title: Manhattan plots generated from TASSEL 5.0 software for six fiber quality traits, A) Elongation (ELO), B) Micronaire (MIC), C) Short fiber content (SFC), D) Fiber strength (STR), E) Upper half mean (UHM) fiber length, and F) Uniformity (UI). The negative log10 transformed p values were plotted against the marker positions on the physical map of each of the 26 Upland cotton chromosome. The significant thresholds (p = 0.01 and 0.0001) are indicated by the purple and green horizontal dot line, respectively. Description of data: Manhattan plots generated from TASSEL 5.0 software for six fiber quality traits from GWAS analysis following MLM are included in this figure. The X and Y axis have chromosome name and observed negative logarithm 10 of p value, respectively. (DOCX 813 kb) [file 12864_2016_3249_MOESM11_ESM.docx]

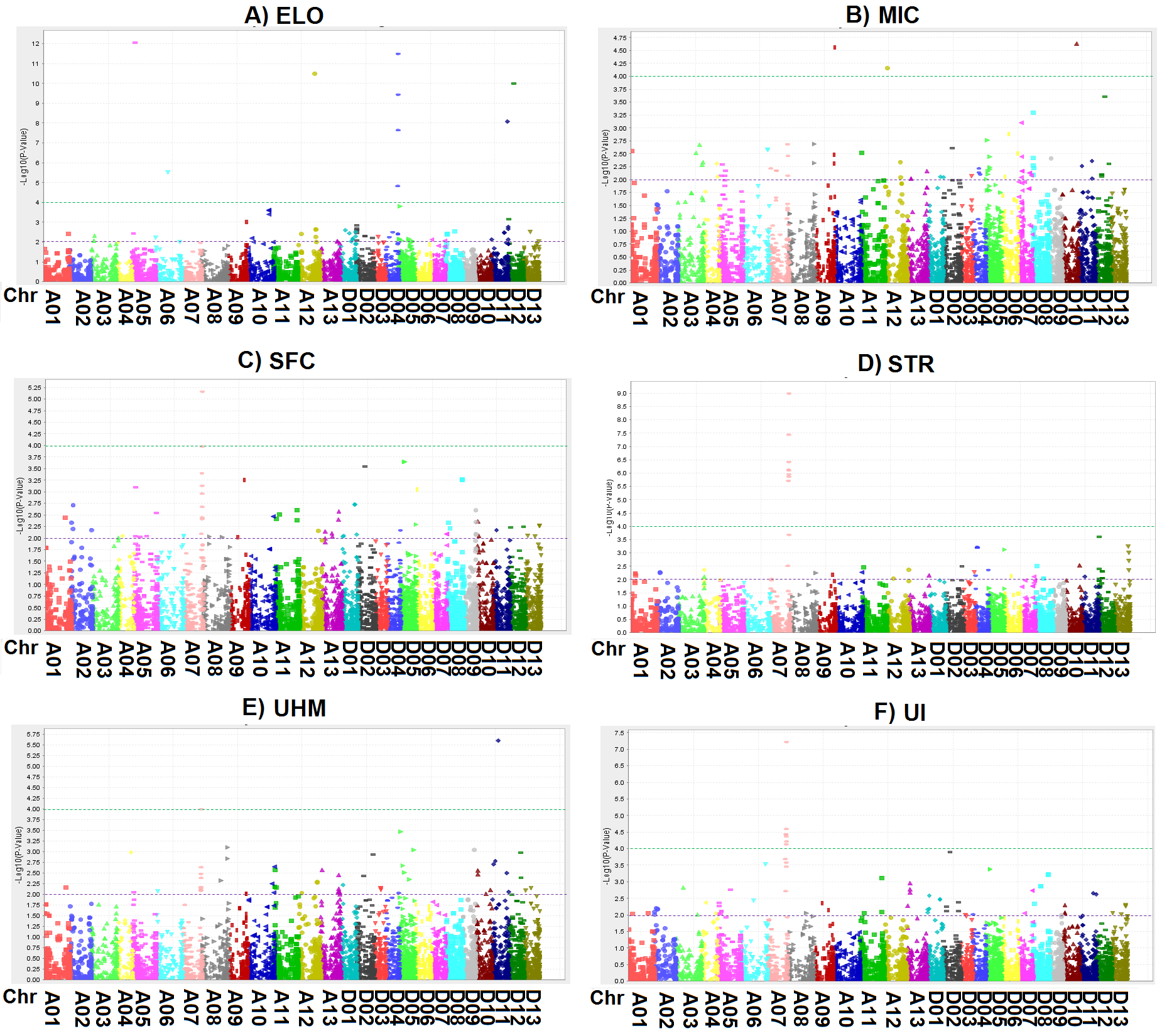
Additional file 11. **Manhattan plots generated from TASSEL 5.0 software for six fiber quality traits,** A) Elongation (ELO), B) Micronaire (MIC), C) Short fiber content (SFC), D) Fiber strength (STR), E) Upper half mean (UHM) fiber length, and F) Uniformity (UI). The negative log_10_ transformed *p* values were plotted against the marker positions on the physical map of each of the 26 Upland cotton chromosome. The significant thresholds (*p* = 0.01 and 0.0001) are indicated by the purple and green horizontal dot line, respectively.
