# Supplementary material for: A MAGIC population-based genome-wide association study reveals functional association of GhRBB1_A07 gene with superior fiber quality in cotton
Source: BMC Genomics. 2016 Nov 9;17:903. doi: 10.1186/s12864-016-3249-2 (PMC5103610; doi:10.1186/s12864-016-3249-2)
Supplement: Additional file 13: — Title: Effect of haplotype grouping near the major QTL on chromosome A07 on fiber bundle strength (g/tex). RILs were divided into two groups (major and minor types) based on genotypes major and minor alleles of respective two SNPs. Description of data: This box plot shows the effect of major and minor allele combination of haplotype group near the QTL on chromosome A07 on fiber strength. The X axis has the fiber strength value (g/tex) and Y axis contain possible major and minor type haplotype groups and their physical locations. (DOCX 101 kb) [file 12864_2016_3249_MOESM13_ESM.docx]

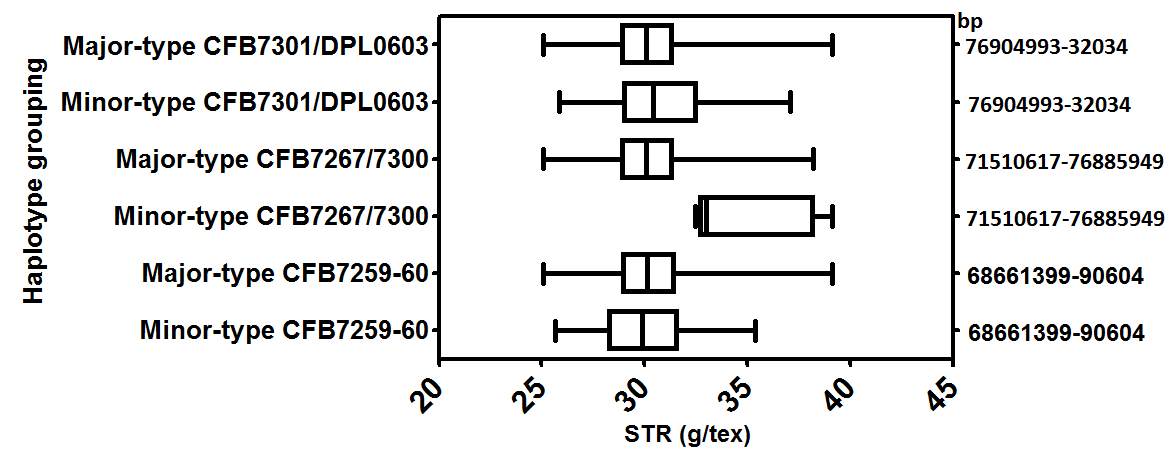
Additional file 13. **Effect of haplotype grouping near the major QTL on chromosome A07 on fiber bundle strength (g/tex)**. RILs were divided into two groups (major and minor types) based on genotypes major and minor alleles of respective two SNPs.
