# Supplementary material for: A MAGIC population-based genome-wide association study reveals functional association of GhRBB1_A07 gene with superior fiber quality in cotton
Source: BMC Genomics. 2016 Nov 9;17:903. doi: 10.1186/s12864-016-3249-2 (PMC5103610; doi:10.1186/s12864-016-3249-2)
Supplement: Additional file 14: — Title: LD contour plot in the Upland cotton genomic region of 71 to 77 Mb on chromosome A07. LD contour was created from genotypic data of 547 RILs of Upland cotton MAGIC population using JMP genomics 6.0 software. X axis is physical distance in Mb and r2 (CorrCoeff2) between marker pair is shown in different color block as per legend. Description of data: LD contour plot of genomic region of 71 to 77 Mb on chromosome A07 is included in this figure. Red color represents the higher r2 value between two markers due to LD block. The square of correlation coefficients (r2) presents as color code (red to blue – 1.000 to 0.000). (DOCX 69 kb) [file 12864_2016_3249_MOESM14_ESM.docx]

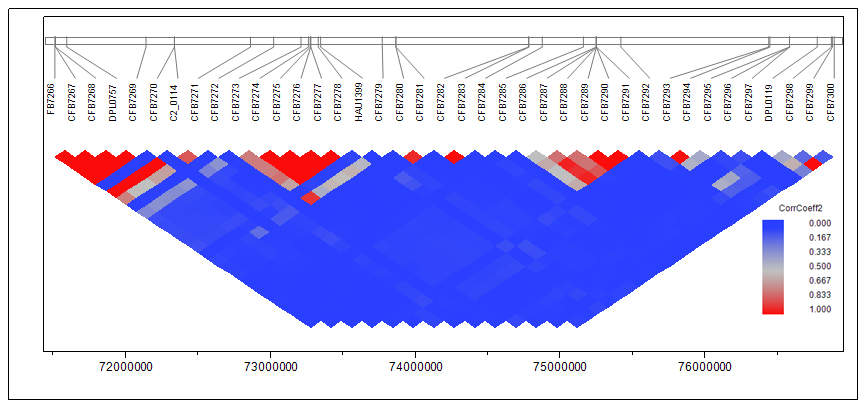
Additional figure 14. **LD contour plot in the Upland cotton genomic region of 71 to 77 Mb on chromosome A07.** LD contour was created from genotypic data of 547 RILs of Upland cotton MAGIC population using JMP genomics 6.0 software. X axis is physical distance in Mb and r^2^ (CorrCoeff2) between marker pair is shown in different color block as per legend.
