# Supplementary material for: A MAGIC population-based genome-wide association study reveals functional association of GhRBB1_A07 gene with superior fiber quality in cotton
Source: BMC Genomics. 2016 Nov 9;17:903. doi: 10.1186/s12864-016-3249-2 (PMC5103610; doi:10.1186/s12864-016-3249-2)
Supplement: Additional file 16: — Title: RT-qPCR gene expression analyses of selected genes related to cell wall activity during fiber development between Acala Ultima (AU) and Tamcot Pyramid (TP). A) CONSTANS-like 9 (Gh_A07G1753); B) RAB GTPase homolog A5E (Gh_A07G1758); C) Unknown protein family, DUF538 (Gh_A07G1784); D) Auxin-responsive protein (Gh_A07G1795); E) Oxidoreductase, zinc-binding dehydrogenase family protein (Gh_A07G1803); F) O-Glycosyl hydrolases family protein (Gh_A07G1802). Description of data: This file contains the results of RT-qPCR analysis data performed on six genes that were used to compare relative expression levels between superior line AU and inferior line TP. Four (8, 14, 28 and 22 DPA) developing fiber samples were used for RT-qPCR analysis. RT-qPCR values were corrected to 18S gene for each sample. For each treatment group two qPCR measurements were taken for each of three biological replicates and then averaged. (DOCX 161 kb) [file 12864_2016_3249_MOESM16_ESM.docx]

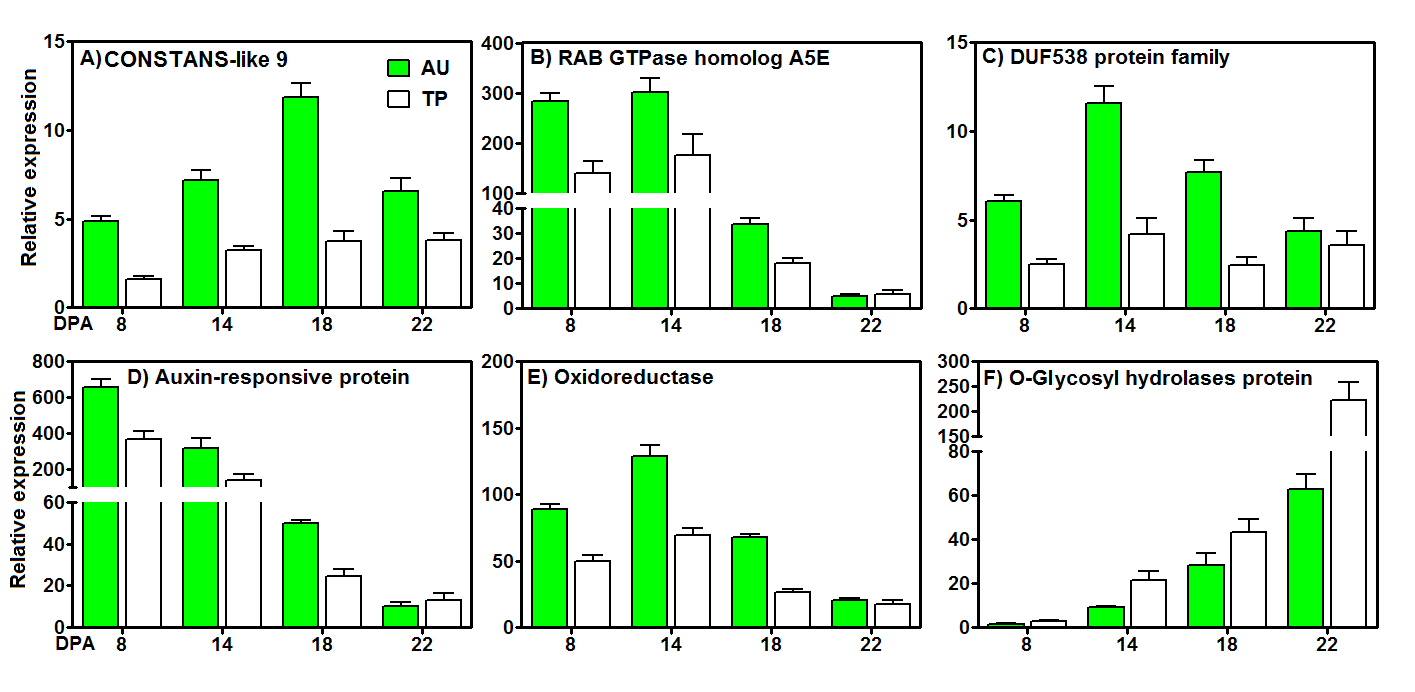
Additional file 16. **RT-qPCR gene expression analyses of selected genes related to cell wall activity during fiber development between Acala Ultima (AU) and Tamcot Pyramid (TP).** A) CONSTANS-like 9 (*Gh_A07G1753*); B) RAB GTPase homolog A5E (*Gh_A07G1758*); C) Unknown protein family, DUF538 (*Gh_A07G1784*); D) Auxin-responsive protein (*Gh_A07G1795*); E) Oxidoreductase, zinc-binding dehydrogenase family protein (*Gh_A07G1803*); F) O-Glycosyl hydrolases family protein (*Gh_A07G1802*).
