# Supplementary material for: A MAGIC population-based genome-wide association study reveals functional association of GhRBB1_A07 gene with superior fiber quality in cotton
Source: BMC Genomics. 2016 Nov 9;17:903. doi: 10.1186/s12864-016-3249-2 (PMC5103610; doi:10.1186/s12864-016-3249-2)

Additional file 17. **Sashimi plot of parental lines showing 18 bp of deletion on parent Acala Ultima at genomic region 76,911,659 to 76,911,676 bp on chromosome A07.**


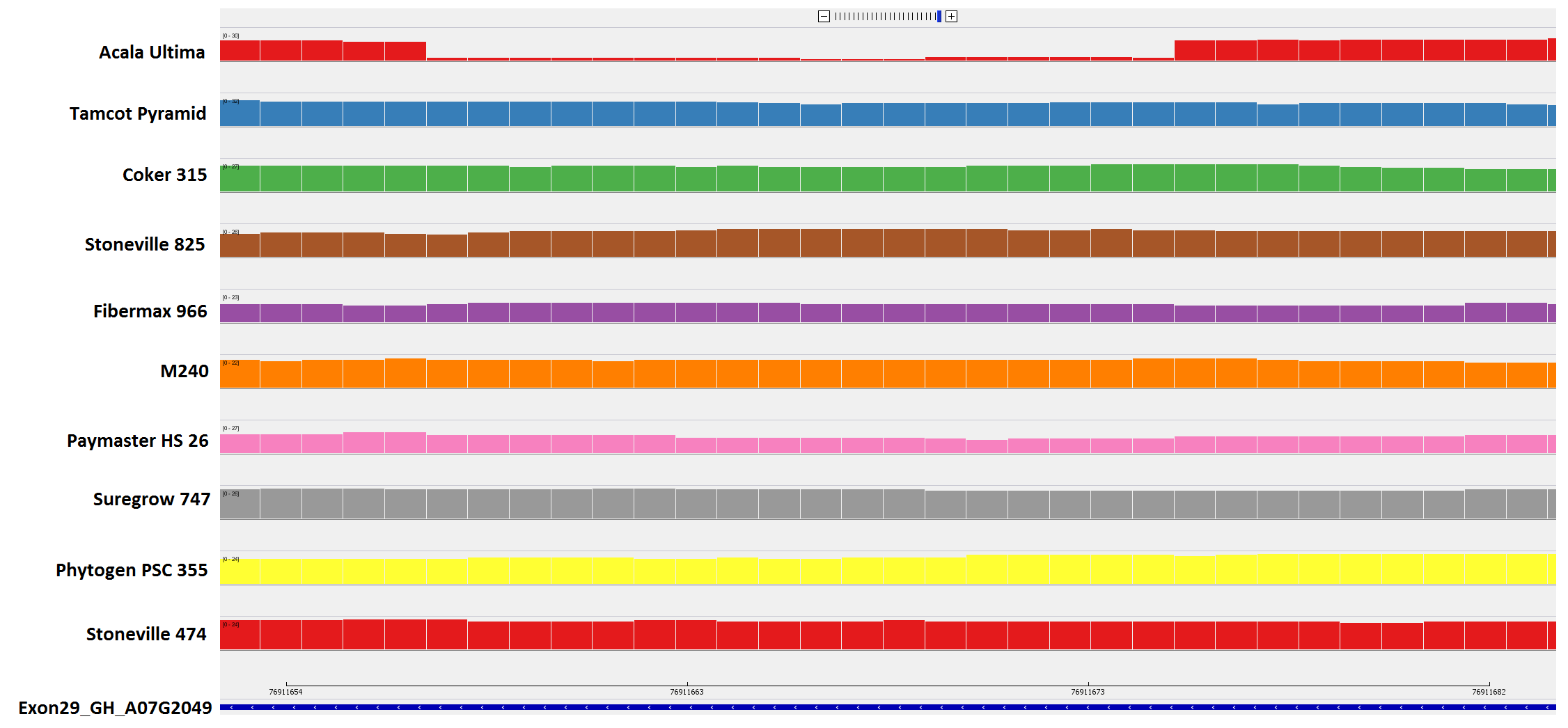

Supplement: Additional file 17: — Title: Sashimi plot of parental lines showing 18 bp of deletion on parent Acala Ultima (AU) at genomic region 76,911,659 to 76,911,676 bp on chromosome A07. Description of data: Sashimi plot of parental lines generated from Integrated Genome Viewer (IGV) software is included in this file. The parent AU (row number 1) has an 18 bp deletion at genomic region 76,911,659 to 76,911,676 bp on exon 29 of gene Gh_A07G2049 on chromosome A07 while other parental lines don’t have that deletion. (DOCX 50 kb) [file 12864_2016_3249_MOESM17_ESM.docx]
