# Supplementary material for: A MAGIC population-based genome-wide association study reveals functional association of GhRBB1_A07 gene with superior fiber quality in cotton
Source: BMC Genomics. 2016 Nov 9;17:903. doi: 10.1186/s12864-016-3249-2 (PMC5103610; doi:10.1186/s12864-016-3249-2)
Supplement: Additional file 18: — Title: Alignment of Acala Ultima (AU) and TM-1 (Ref) alleles of Gh_A07G2049. Primer locations for CFBid0004 are highlighted yellow. Description of data: This file contains alignment of parental line AU (row number 1) and TM-1 (row number 2) alleles of gene Gh_A07G2049. The 18 bp deletion in the coding sequence in AU at 17,875 to 17,893 bp on gene has also been presented. The forward and reverse primer sequences for indel marker CFBid0004 has been highlighted as yellow at either side of the 18 bp deletion. (PDF 4439 kb) [file 12864_2016_3249_MOESM18_ESM.pdf]

Additional file 18. Alignment of Acala Ultima (AU) and TM-1 (Ref) alleles of Gh_A07G2049. Primer locations for *CFBid0004* are highlighted yellow.

```
                      20                  40                  60                  80
AU  AATCTTCATT TCTTGCTCTC CTTTGGTGAT ATTGGTTTCG ATTCTTTTGA ATGAGATGAT TTACCAGTAA TTGCCTTCTT CATCTTGGAA 90
Ref AATCTTCATT TCTTGCTCTC CTTTGGTGAT ATTGGTTTCG ATTCTTTTGA ATGAGATGAT TTACCAGTAA TTGCCTTCTT CATCTTGGAA 90
            100                 120                 140                 160                 180
AU  ATCGAATGCT TCACTTTCGA TCCAACACCT GACAGTATGT TATGAGATTT TTTATGTGGT TTAATACCGG GTTCTGTCTT ACTGTTTTCA 180
Ref ATCGAATGCT TCACTTTCGA TCCAACACCT GACAGTATGT TATGAGATTT TTTATGTGGT TTAATACCGG GTTCTGTCTT ACTGTTTTCA 180
                      200                 220                 240                 260
AU  TCACCTTCAT CTTCATCTGT TTTTGCTTCT TCCTCTGGTG GTTCTTCCTT GCTTACCACT AGTTCCTTTT CTTCAATCAC ATTTTTAGCT 270
Ref TCACCTTCAT CTTCATCTGT TTTTGCTTCT TCCTCTGGTG GTTCTTCCTT GCTTACCACT AGTTCCTTTT CTTCAATCAC ATTTTTAGCT 270
            280                 300                 320                 340                 360
AU  CCTTGTAATC CTCTTGTGGA TCTTTGGAGT AGGTCATACA ATGATAGCTT AGCTGAATCC TCTGATTCAG GTTCCTCAGA TTTTTCAGCA 360
Ref CCTTGTAATC CTCTTGTGGA TCTTTGGAGT AGGTCATACA ATGATAGCTT AGCTGAATCC TCTGATTCAG GTTCCTCAGA TTTTTCAGCA 360
                      380                 400                 420                 440
AU  CCTTCATTCT CTGGTCTTTT CTCTTTTCCA TTATCGGGTC CTGCTTCTGA ATATATCTCT TGAGCATTTC TAGGTTCCAA ATCTCCAACT 450
Ref CCTTCATTCT CTGGTCTTTT CTCTTTTCCA TTATCGGGTC CTGCTTCTGA ATATATCTCT TGAGCATTTC TAGGTTCCAA ATCTCCAACT 450
            460                 480                 500                 520                 540
AU  ACAATTTTGT CTGAGGTTTC AGAAGCTTCA TCCTGCTGTT CTGTTGGAAA CTGTTGCTTG CCAATTGAAT TTGTTGTCTT TTTGCACTCA 540
Ref ACAATTTTGT CTGAGGTTTC AGAAGCTTCA TCCTGCTGTT CTGTTGGAAA CTGTTGCTTG CCAATTGAAT TTGTTGTCTT TTTGCACTCA 540
                      560                 580                 600                 620
AU  ATAGAATCTT TCTCTCCTGG AATCTGTTCG CCAAATGTTA CATTAGTGAT ATATCTATGC TGGACATAGT ATGTTATGTA ATGACTTATG 630
Ref ATAGAATCTT TCTCTCCTGG AATCTGTTCG CCAAATGTTA CATTAGTGAT ATATCTATGC TGGACATAGT ATGTTATGTA ATGACTTATG 630
            640                 660                 680                 700                 720
AU  TTTAGACTCC ATGGATGGAC CTTGAAAAGG ATTCTTTTAG AGCCCTCTTA AACTACATTA AGCTTGAAGT TGTTTCATTG CCTTACTCAT 720
Ref TTTAGACTCC ATGGATGGAC CTTGAAAAGG ATTCTTTTAG AGCCCTCTTA AACTACATTA AGCTTGAAGT TGTTTCATTG CCTTACTCAT 720
                      740                 760                 780                 800
AU  AATGTGATAT CATTTTTCTT ACTAGATTCT TACCTCCTCT ACTATAGTGG CTTCTTTCAT GTCATCCTTC ACAGTTTCAT CTTTAGCAAC 810
Ref AATGTGATAT CATTTTTCTT ACTAGATTCT TACCTCCTCT ACTATAGTGG CTTCTTTCAT GTCATCCTTC ACAGTTTCAT CTTTAGCAAC 810
            820                 840                 860                 880                 900
AU  GGTCTCTTTC TTGATATTGT CTCTTGCAGC ATCCTTTGGA GTTTCCAAGG TGGGCTGTTG TGAATTTAAG ATTCAAGATC AAAAGAGAAG 900
Ref GGTCTCTTTC TTGATATTGT CTCTTGCAGC ATCCTTTGGA GTTTCCAAGG TGGGCTGTTG TGAATTTAAG ATTCAAGATC AAAAGAGAAG 900
                      920                 940                 960                 980
AU  AGGCAAGAAA AGAACCAAAA GATTATAGAT GAGCATTTCG AGAACTGACC TTTTCATTTT GAATTTCATA TGTTGGGACT TCGAAGGTTT 990
Ref AGGCAAGAAA AGAACCAAAA GATTATAGAT GAGCATTTCG AGAACTGACC TTTTCATTTT GAATTTCATA TGTTGGGACT TCGAAGGTTT 990
            1,000               1,020               1,040               1,060               1,080
AU  CTTTTGTTTT GTCTCCAGTA TGTTCATCTT CTTTGCTGCC ATCTTCAAAA TGAGAAATCA GGTCCTCATT GATCTGTCAA GATTGCTCCC 1080
Ref CTTTTGTTTT GTCTCCAGTA TGTTCATCTT CTTTGCTGCC ATCTTCAAAA TGAGAAATCA GGTCCTCATT GATCTGTCAA GATTGCTCCC 1080
                      1,100               1,120               1,140               1,160
AU  ATTGTTAGCA GTTGTTGAGT TCATATATAA AGTGTAAGAG CAAAACAAAA AGAAGAGAGA AAATGGAACC AGTGAAGAGT GCATGAAATT 1170
Ref ATTGTTAGCA GTTGTTGAGT TCATATATAA AGTGTAAGAG CAAAACAAAA AGAAGAGAGA AAATGGAACC AGTGAAGAGT GCATGAAATT 1170
            1,180               1,200               1,220               1,240               1,260
AU  ATTAAGGTTT AAATTGGATC TAACCTTTGT TTGTGGAGGT TGGATAACTA TAGATTTCTC CTCCACAACA GATTTTAGCT CCTTGTTTTC 1260
Ref ATTAAGGTTT AAATTGGATC TAACCTTTGT TTGTGGAGGT TGGATAACTA TAGATTTCTC CTCCACAACA GATTTTAGCT CCTTGTTTTC 1260
                      1,280               1,300               1,320               1,340
AU  TTCCTGCTCC GAGCATATAT TGTCTCCAAT CTTCATATCT GAAGAATCTT TATCTCGAGT TTCCACTTCC ATCTGTTCTT TTTCTTCTCC 1350
Ref TTCCTGCTCC GAGCATATAT TGTCTCCAAT CTTCATATCT GAAGAATCTT TATCTCGAGT TTCCACTTCC ATCTGTTCTT TTTCTTCTCC 1350
            1,360               1,380               1,400               1,420               1,440
AU  TGCATCTACT GGAGCTGGAT ATTTATTCTC TTGTTTGTCC ACAAGAAGAA ATCGGGAAGC ATCAGTTTCA TCTTTTGCTT CTTTCTCAAT 1440
Ref TGCATCTACT GGAGCTGGAT ATTTATTCTC TTGTTTGTCC ACAAGAAGAA ATCGGGAAGC ATCAGTTTCA TCTTTTGCTT CTTTCTCAAT 1440
                      1,460               1,480               1,500               1,520
AU  CGTGTCTCGA GTCAATTGTG AAGTTCCTTT CGTGAAGTTT TCGACATGGT CAGATGATGA TATCTTAGTC AAATCTTCCA TGGATACAGA 1530
Ref CGTGTCTCGA GTCAATTGTG AAGTTCCTTT CGTGAAGTTT TCGACATGGT CAGATGATGA TATCTTAGTC AAATCTTCCA TGGATACAGA 1530
            1,540               1,560               1,580               1,600               1,620
AU  CTCCTTGATT ATTTCTTCAC TTGAATTATT TACTGTTTTT TCTCCAGCAT GTTCTATTCC TCCTGAATAG ATCTCTTTGA CATCTCCGAT 1620
Ref CTCCTTGATT ATTTCTTCAC TTGAATTATT TACTGTTTTT TCTCCAGCAT GTTCTATTCC TCCTGAATAG ATCTCTTTGA CATCTCCGAT 1620
                      1,640               1,660               1,680               1,700
AU  TTCAGCCTTT TGCGGATCAA CTCTGTCCTG CAAATCTATC CGAACATATT CCCTGACGAC CAAACTTGTT ATCTCCATGT TCTCAATATG 1710
Ref TTCAGCCTTT TGCGGATCAA CTCTGTCCTG CAAATCTATC CGAACATATT CCCTGACGAC CAAACTTGTT ATCTCCATGT TCTCAATATG 1710
            1,720               1,740               1,760               1,780               1,800
AU  ATCAAATTCC CTTGGAATCT GCTTATCAGA TGCTGTCTTT GTTGTTTCAT CAGTCTGCTG AATATCATTG AGTATTTGAT TGCCTTCTGC 1800
Ref ATCAAATTCC CTTGGAATCT GCTTATCAGA TGCTGTCTTT GTTGTTTCAT CAGTCTGCTG AATATCATTG AGTATTTGAT TGCCTTCTGC 1800
                      1,820               1,840               1,860               1,880
AU  AATGGATATT TTGGAGCTTG ATTCCTGATA AAAGATGAAT GAAGAGCTGC TTAGATTTAT GAAGTCAAAC AAAACTGTTC TGTTTCCAAC 1890
Ref AATGGATATT TTGGAGCTTG ATTCCTGATA AAAGATGAAT GAAGAGCTGC TTAGATTTAT GAAGTCAAAC AAAACTGTTC TGTTTCCAAC 1890
            1,900               1,920               1,940               1,960               1,980
AU  TAGAGTTTCA TTTGCATAAA ATATTAGGCA AAGTTCGAAA GGAGGGTTCA TACTATTGTG GTTTCAATCC TGACAGCTGA AGCTTGGCCT 1980
Ref TAGAGTTTCA TTTGCATAAA ATATTAGGCA AAGTTCGAAA GGAGGGTTCA TACTATTGTG GTTTCAATCC TGACAGCTGA AGCTTGGCCT 1980
                      2,000               2,020               2,040               2,060
AU  CGATCCTCAA CAACTGCATT TTTGATCTGC TTTTCGATTA TCTCATTCTC ACTGGTCTTT TGAGCTTCCA CAATGAACTG TTATAGATCA 2070
Ref CGATCCTCAA CAACTGCATT TTTGATCTGC TTTTCGATTA TCTCATTCTC ACTGGTCTTT TGAGCTTCCA CAATGAACTG TTATAGATCA 2070
            2,080               2,100               2,120               2,140               2,160
AU  AAGGTTCAAT TTAGATATTA AAAAACATGA GCGAATACGG TGAACAAAGT TTAGTTTCAG TTTCCTGACC TCTTCAAATG TATTGTCTTC 2160
Ref AAGGTTCAAT TTAGATATTA AAAAACATGA GCGAATACGG TGAACAAAGT TTAGTTTCAG TTTCCTGACC TCTTCAAATG TATTGTCTTC 2160
                      2,180               2,200               2,220               2,240
AU  AATCGGTTCA TCCTTACCCA CATTTTCTCT TGCCTCATCT TTGATCGGCT CTTCTGAATG GGAAGTCGAA TAAGATGCAA CCTGTTGATT 2250
Ref AATCGGTTCA TCCTTACCCA CATTTTCTCT TGCCTCATCT TTGATCGGCT CTTCTGAATG GGAAGTCGAA TAAGATGCAA CCTGTTGATT 2250
```

```
                          2,260                2,280                2,300                2,320                2,340
AU  TTGGCCACTC AGTTAGTGGT TATCGAAGAC AATAATAGAA AGGAGAATGA ATAAGAAATA CATGCAATTA TTAAGGTTTT GAGCTAGTAT 2340
Ref TTGGCCACTC AGTTAGTGGT TATCGAAGAC AATAATAGAA AGGAGAATGA ATAAGAAATA CATGCAATTA TTAAGGTTTT GAGCTAGTAT 2340
                                     2,360                2,380                2,400                2,420
AU  TGCTTACCTC TTCTTTTGAA ACTTCATTCA GTATGATTTC ATTTCCTTCA TTTGTTTGCT CACACTGATT TTCTTCACCG TCTGTCCCAT 2430
Ref TGCTTACCTC TTCTTTTGAA ACTTCATTCA GTATGATTTC ATTTCCTTCA TTTGTTTGCT CACACTGATT TTCTTCACCG TCTGTCCCAT 2430
                          2,440                2,460                2,480                2,500                2,520
AU  CTCTTGCTTT GACAATATTC TCTTTGATTG CAGCATCTTT TGTTTCTTCG TTGAGTAGTT CATTGTCAAA AGCCTTTTCA TGTATCTCCC 2520
Ref CTCTTGCTTT GACAATATTC TCTTTGATTG CAGCATCTTT TGTTTCTTCG TTGAGTAGTT CATTGTCAAA AGCCTTTTCA TGTATCTCCC 2520
                                     2,540                2,560                2,580                2,600
AU  TTTTGAAATT CGAAGCTTCT TCAACCTCTC CTTTTAGTTC CGGAATCTTC TCGTGACCCT CATCTTCTTT AAATTCTGTT TTATTTGTTT 2610
Ref TTTTGAAATT CGAAGCTTCT TCAACCTCTC CTTTTAGTTC CGGAATCTTC TCGTGACCCT CATCTTCTTT AAATTCTGTT TTATTTGTTT 2610
                          2,620                2,640                2,660                2,680                2,700
AU  CATCTCGTTG GACCTCAGAA GGCAATACAG AGGTTGTGAT ATGATGGTGG TTAGTTTCAC CTCTCAAAAT GGCTTCGGCT TCCTTTATCT 2700
Ref CATCTCGTTG GACCTCAGAA GGCAATACAG AGGTTGTGAT ATGATGGTGG TTAGTTTCAC CTCTCAAAAT GGCTTCGGCT TCCTTTATCT 2700
                                     2,720                2,740                2,760                2,780
AU  TGTCCTTTTC CTGGCTTATC TTTTCCTCTT TCTTTACTCC ATCAATGTGT CCATTTTCAG TTTCTTTTTC CTTCATATCC TCCTCCTCGG 2790
Ref TGTCCTTTTC CTGGCTTATC TTTTCCTCTT TCTTTACTCC ATCAATGTGT CCATTTTCAG TTTCTTTTTC CTTCATATCC TCCTCCTCGG 2790
                          2,800                2,820                2,840                2,860                2,880
AU  TATTGCCAAC TTCATCAACA GTTTTGTGCT TATACTCCTC AAATTGCAAT GTGGCAACCA AATTTGAAAT TTGCTCCTTG GGTTTCTCTG 2880
Ref TATTGCCAAC TTCATCAACA GTTTTGTGCT TATACTCCTC AAATTGCAAT GTGGCAACCA AATTTGAAAT TTGCTCCTTG GGTTTCTCTG 2880
                                     2,900                2,920                2,940                2,960
AU  TTGCGGTAGT CTGAGAAGTA GCATTATCAT CTACTGCAAG AGCTTCATCC TCCTTAAGCT GTTCCTTTTG CAAGGTCACA TCATCTCTAA 2970
Ref TTGCGGTAGT CTGAGAAGTA GCATTATCAT CTACTGCAAG AGCTTCATCC TCCTTAAGCT GTTCCTTTTG CAAGGTCACA TCATCTCTAA 2970
                          2,980                3,000                3,020                3,040                3,060
AU  TATCACTTCG TAAGAATTTT GCTTCCTCCA TTTTCTTACA TTCTATCTTC CCGGCTGTAC CAGCAGTCAT TTCATCTTCT TTGACAGCTT 3060
Ref TATCACTTCG TAAGAATTTT GCTTCCTCCA TTTTCTTACA TTCTATCTTC CCGGCTGTAC CAGCAGTCAT TTCATCTTCT TTGACAGCTT 3060
                                     3,080                3,100                3,120                3,140
AU  CTAGCCCAAT GGAACTTCGC TTGTGTGAGC TTTTCTCTAC TGTAATCTGT GCATCAGAAG CATCTTCTTC TGTCTTGTCT TTTATGATTT 3150
Ref CTAGCCCAAT GGAACTTCGC TTGTGTGAGC TTTTCTCTAC TGTAATCTGT GCATCAGAAG CATCTTCTTC TGTCTTGTCT TTTATGATTT 3150
                          3,160                3,180                3,200                3,220                3,240
AU  CCCCTTCGCT TGTTTTCTCA TCTTCTGTCT CCTTAGTTTC AGGCTCACCA GGTCCTTCCT TTTCAAGCAT TTCAGGTTCC TTCACCTTTT 3240
Ref CCCCTTCGCT TGTTTTCTCA TCTTCTGTCT CCTTAGTTTC AGGCTCACCA GGTCCTTCCT TTTCAAGCAT TTCAGGTTCC TTCACCTTTT 3240
                                     3,260                3,280                3,300                3,320
AU  CTTCTTCTTT GCTGGCATCA GCTTCATGCT TTGGGTTCTG AACAGGGAAT TCTAAATACA AAGATTGTTC ATTTGATTCC TCCATTTGAA 3330
Ref CTTCTTCTTT GCTGGCATCA GCTTCATGCT TTGGGTTCTG AACAGGGAAT TCTAAATACA AAGATTGTTC ATTTGATTCC TCCATTTGAA 3330
                          3,340                3,360                3,380                3,400                3,420
AU  GAGCTTGAGC AGCAGCAGTT TCATCGTTTG TTGCATCATT AAGCTCCTGA AACTCTTGTT TTTCTAAGCA TGTCTCAGCT TTTTTCTTTG 3420
Ref GAGCTTGAGC AGCAGCAGTT TCATCGTTTG TTGCATCATT AAGCTCCTGA AACTCTTGTT TTTCTAAGCA TGTCTCAGCT TTTTTCTTTG 3420
                                     3,440                3,460                3,480                3,500
AU  CATCAGAAGG ATCTTCTGAA ATCTTGTCCG GACTCATGTC TGCTTCATTT TTTTTCTCTT CTTCCATCTT TTCAGCTTTG CTCACATTTT 3510
Ref CATCAGAAGG ATCTTCTGAA ATCTTGTCCG GACTCATGTC TGCTTCATTT TTTTTCTCTT CTTCCATCTT TTCAGCTTTG CTCACATTTT 3510
                          3,520                3,540                3,560                3,580                3,600
AU  CTTCTTCGTC CGAAACTAAT GGAGAGCTCG TAGTCTTCTC CTCGGGTTTA TTAGTCTCAA GAGTCTGCTC ACCAGTTATT CCATCTCCTG 3600
Ref CTTCTTCGTC CGAAACTAAT GGAGAGCTCG TAGTCTTCTC CTCGGGTTTA TTAGTCTCAA GAGTCTGCTC ACCAGTTATT CCATCTCCTG 3600
                                     3,620                3,640                3,660                3,680
AU  CAACAGCATC TAGCTTTGTG GGTATTTCTT CCTTTGTTTT GCCATATTCT ATCTCCTCAT TTATGGCTCC AGTTCCATGT TCGCTTTCCT 3690
Ref CAACAGCATC TAGCTTTGTG GGTATTTCTT CCTTTGTTTT GCCATATTCT ATCTCCTCAT TTATGGCTCC AGTTCCATGT TCGCTTTCCT 3690
                          3,700                3,720                3,740                3,760                3,780
AU  TGGAAGCCAA TGTTGAAAAT GTAGTTTGAA TTTTCTCCTC CGGTTCCACA TTAGGAAGAC CCTCATCAGC AATTGTCTCT ACTGCTTTAA 3780
Ref TGGAAGCCAA TGTTGAAAAT GTAGTTTGAA TTTTCTCCTC CGGTTCCACA TTAGGAAGAC CCTCATCAGC AATTGTCTCT ACTGCTTTAA 3780
                                     3,800                3,820                3,840                3,860
AU  GCTCTCCTTG TTCTTCATTT GGCAAGCGTA CCTCTTCTAT TCTCTTTGTG TCAGAAGAAT CTTCTCGGCT CTCGTGTTTT ATTTCAGCAT 3870
Ref GCTCTCCTTG TTCTTCATTT GGCAAGCGTA CCTCTTCTAT TCTCTTTGTG TCAGAAGAAT CTTCTCGGCT CTCGTGTTTT ATTTCAGCAT 3870
                          3,880                3,900                3,920                3,940                3,960
AU  CCTTCTGGAT CTCTTCTTCA ATATTCTCAA TCGGGTTTGC AATTCTGATA TTTTCACCCT CAGAAAGCAA TGTGCAAGCA GAACTTTGAA 3960
Ref CCTTCTGGAT CTCTTCTTCA ATATTCTCAA TCGGGTTTGC AATTCTGATA TTTTCACCCT CAGAAAGCAA TGTGCAAGCA GAACTTTGAA 3960
                                     3,980                4,000                4,020                4,040
AU  GTTGCTCATC TGGTTTCTCT TCTGGAAGAG TATTGGTTGA ACCAATATCA TCGATGAGAA TAGTTTCAAC TCTTTGAGAG ATGTAATCAG 4050
Ref GTTGCTCATC TGGTTTCTCT TCTGGAAGAG TATTGGTTGA ACCAATATCA TCGATGAGAA TAGTTTCAAC TCTTTGAGAG ATGTAATCAG 4050
                          4,060                4,080                4,100                4,120                4,140
AU  ATGATGTTTC AGGTACTCCT TCAATGTCTT CAGTTTTATT GTCTTCCAGT TCTGCTGTTG GGCCTTTCTC AGGTTCAGCA ACCTGATCTT 4140
Ref ATGATGTTTC AGGTACTCCT TCAATGTCTT CAGTTTTATT GTCTTCCAGT TCTGCTGTTG GGCCTTTCTC AGGTTCAGCA ACCTGATCTT 4140
                                     4,160                4,180                4,200                4,220
AU  TGTGCATGTA AAGTGCATCT GCATCTCTCT TGATTACCTC CACTTCATTG TTGTCATTAA GTGGGACTTC AAGTGCCAAG TCGGAGGATA 4230
Ref TGTGCATGTA AAGTGCATCT GCATCTCTCT TGATTACCTC CACTTCATTG TTGTCATTAA GTGGGACTTC AAGTGCCAAG TCGGAGGATA 4230
                          4,240                4,260                4,280                4,300                4,320
AU  TATCTTCAAG CTGCCCCATC TCTACTTCTT TAGTCTCTTG ATTGGCATCA TCTTTAATGG TTTCTAGACC CATCTTGACT GAGCTGATAT 4320
Ref TATCTTCAAG CTGCCCCATC TCTACTTCTT TAGTCTCTTG ATTGGCATCA TCTTTAATGG TTTCTAGACC CATCTTGACT GAGCTGATAT 4320
                                     4,340                4,360                4,380                4,400
AU  TCAATGTATT TATGAGCTCC TCATTCACAG CATCGTTTAA GCTCATCTGT GCTCTTAAGT CTGTCGCAAC TTCAATATGA CTCTCTTCAC 4410
Ref TCAATGTATT TATGAGCTCC TCATTCACAG CATCGTTTAA GCTCATCTGT GCTCTTAAGT CTGTCGCAAC TTCAATATGA CTCTCTTCAC 4410
                          4,420                4,440                4,460                4,480                4,500
AU  GGCAGAGTTT CACGTCATCG ATAATCTCAG CTTTTTCTTC TGCTTCTCTA TCTTCACTGG ACACTTTTGC TGGATCTGTG GACTCTTCAC 4500
Ref GGCAGAGTTT CACGTCATCG ATAATCTCAG CTTTTTCTTC TGCTTCTCTA TCTTCACTGG ACACTTTTGC TGGATCTGTG GACTCTTCAC 4500
```

```
                   4,520                4,540                4,560                4,580
AU  CCTGTCTCCT TTCTTCTAAT TCTTCTATAA TGTGGGATCC TTCTTTGTCA TGGGTTGATT TTCTTTCAAC CTCTGTTTGT TCTGCTTCTA 4590
Ref CCTGTCTCCT TTCTTCTAAT TCTTCTATAA TGTGGGATCC TTCTTTGTCA TGGGTTGATT TTCTTTCAAC CTCTGTTTGT TCTGCTTCTA 4590
        4,600                4,620                4,640                4,660                4,680
AU  TGTGCTCTCT GGACTCAGCT TCCTTGATAC ATTCAGTTGA GACTGAATTG TAGTCTTTTG CCTGTTAACA CATAGTTAAA ATGTTTGAGA 4680
Ref TGTGCTCTCT GGACTCAGCT TCCTTGATAC ATTCAGTTGA GACTGAATTG TAGTCTTTTG CCTGTTAACA CATAGTTAAA ATGTTTGAGA 4680
                   4,700                4,720                4,740                4,760
AU  TTTTGACACA TAAAAAGAAC TATTTTAATA GAAGTATATA ACTATTTATT AAGAATTACT GAGATGACCT TATTTGCTTC ATCTCCTCTT 4770
Ref TTTTGACACA TAAAAAGAAC TATTTTAATA GAAGTATATA ACTATTTATT AAGAATTACT GAGATGACCT TATTTGCTTC ATCTCCTCTT 4770
        4,780                4,800                4,820                4,840                4,860
AU  GATACTGGAG GAATTTCATT TGTACTACTT TCTTCCTCCA TGACATGTTC TTTGATGGCT TCACTAACAT CAGCTTTCTC AACTTCCTTA 4860
Ref GATACTGGAG GAATTTCATT TGTACTACTT TCTTCCTCCA TGACATGTTC TTTGATGGCT TCACTAACAT CAGCTTTCTC AACTTCCTTA 4860
                   4,880                4,900                4,920                4,940
AU  TCAACCTGCC AACAATGATA ATGTAACTGA AATTCACCTT GAATAAACAT GAAGACAAAA TATCTTACAA GCTCTTTACC TTTTCAGACA 4950
Ref TCAACCTGCC AACAATGATA ATGTAACTGA AATTCACCTT GAATAAACAT GAAGACAAAA TATCTTACAA GCTCTTTACC TTTTCAGACA 4950
        4,960                4,980                5,000                5,020                5,040
AU  AAACGACAAC GTTGGTAACC TTGTTTTCAT TTTCTTCTCT GTTAATATAG TCTTCATCCT TCAGTTTGTC TTTCAGTTCC TCTGTAATAT 5040
Ref AAACGACAAC GTTGGTAACC TTGTTTTCAT TTTCTTCTCT GTTAATATAG TCTTCATCCT TCAGTTTGTC TTTCAGTTCC TCTGTAATAT 5040
                   5,060                5,080                5,100                5,120
AU  GTTCCTTGAT TTCTTGACTG GTTTCATCAT CTTGGCTCTT GGTGTCTTCA GGGATTGTTT CATTACTTTG GTCTTCAGCC ACGGGTTCCG 5130
Ref GTTCCTTGAT TTCTTGACTG GTTTCATCAT CTTGGCTCTT GGTGTCTTCA GGGATTGTTT CATTACTTTG GTCTTCAGCC ACGGGTTCCG 5130
        5,140                5,160                5,180                5,200                5,220
AU  AGTCGGATTT GGGCTTTTCT TCAAAACCTG CTAGTGCTGG CTCAACATTC TGTTCGAAAT TAATACATTA GTCGAGGGCT TGAGAAATCT 5220
Ref AGTCGGATTT GGGCTTTTCT TCAAAACCTG CTAGTGCTGG CTCAACATTC TGTTCGAAAT TAATACATTA GTCGAGGGCT TGAGAAATCT 5220
                   5,240                5,260                5,280                5,300
AU  GCTACTTAAA TGACGAGAAG CAATTGAATG GTGATAACTG ATATGACAAA CGAACAAGAT TAGACATACT ATACAACTAT GTTTTTCCAT 5310
Ref GCTACTTAAA TGACGAGAAG CAATTGAATG GTGATAACTG ATATGACAAA CGAACAAGAT TAGACATACT ATACAACTAT GTTTTTCCAT 5310
        5,320                5,340                5,360                5,380                5,400
AU  TATCGTATAC ATATGAAGTT TTGGTTTTTG TTTTCTATTG ATAATTTGAT TTGACGAGGT TCTTCATTGA GTTAACCATT TTCTATACTA 5400
Ref TATCGTATAC ATATGAAGTT TTGGTTTTTG TTTTCTATTG ATAATTTGAT TTGACGAGGT TCTTCATTGA GTTAACCATT TTCTATACTA 5400
                   5,420                5,440                5,460                5,480
AU  AAACCTGAAT CCAGTTTGTC ATTGGAACGT GAATTCAACA AGTCTGTTGC AGTTCCTATT ACATTTACAT TTGCATAAAA TATTATGCTA 5490
Ref AAACCTGAAT CCAGTTTGTC ATTGGAACGT GAATTCAACA AGTCTGTTGC AGTTCCTATT ACATTTACAT TTGCATAAAA TATTATGCTA 5490
        5,500                5,520                5,540                5,560                5,580
AU  ACTTTGAAAG GAGGGTTGAA AGTAGTCTGA CCTCTTCTGT TACTGTTATA GTTTCAATCC TGGAAACTGA AGCATGGCTT GGATCCTCAA 5580
Ref ACTTTGAAAG GAGGGTTGAA AGTAGTCTGA CCTCTTCTGT TACTGTTATA GTTTCAATCC TGGAAACTGA AGCATGGCTT GGATCCTCAA 5580
                   5,600                5,620                5,640                5,660
AU  AAGTTTTATC TTCAATCTGG TTTTCAATTA TCTCAATTTC ACTGGTCTTT TGAGCTTCCT GAACGAGCTG TTATAGATTT AATATTCAAT 5670
Ref AAGTTTTATC TTCAATCTGG TTTTCAATTA TCTCAATTTC ACTGGTCTTT TGAGCTTCCT GAACGAGCTG TTATAGATTT AATATTCAAT 5670
        5,680                5,700                5,720                5,740                5,760
AU  ATGTATTTAT ATAAGAGAAA AAAATGGAAG ATGAACAAAG TTATAGTTTC TGTTTCCTTA CCTCTCCATA TGTCTTGCCT TCAGTCGGGT 5760
Ref ATGTATTTAT ATAAGAGAAA AAAATGGAAG ATGAACAAAG TTATAGTTTC TGTTTCCTTA CCTCTCCATA TGTCTTGCCT TCAGTCGGGT 5760
                   5,780                5,800                5,820                5,840
AU  TTTCCTTTAC CTCATCTTCC CCAGAGCCAT CTTTGATCAG CTCTTCCGAA CAGGAAGTTG AATAAGTTGT TTCAGTAACT TTCTTGTTCT 5850
Ref TTTCCTTTAC CTCATCTTCC CCAGAGCCAT CTTTGATCAG CTCTTCCGAA CAGGAAGTTG AATAAGTTGT TTCAGTAACT TTCTTGTTCT 5850
        5,860                5,880                5,900                5,920                5,940
AU  TTTTGACCTG TCAAGTTTGA CCCTTAGTTA ATAGTCATCT AGATAACAAT AGAAACGAGA ATACATAGAA AATAGATGAA AAATTTATGA 5940
Ref TTTTGACCTG TCAAGTTTGA CCCTTAGTTA ATAGTCATCT AGATAACAAT AGAAACGAGA ATACATAGAA AATAGATGAA AAATTTATGA 5940
                   5,960                5,980                6,000                6,020
AU  TTTTTGAGCT AGTATTGGTT ACCTGTTCTA TCGAAACTTC ATTCATTGGG ATTTCATTTC CTTCATTTGT TTTCTCACAC TGGTTTACTT 6030
Ref TTTTTGAGCT AGTATTGGTT ACCTGTTCTA TCGAAACTTC ATTCATTGGG ATTTCATTTC CTTCATTTGT TTTCTCACAC TGGTTTACTT 6030
        6,040                6,060                6,080                6,100                6,120
AU  CACTCCCTGA CCCATCCCTT GCTTTGACAA GATTCTCTTT GATTGCAGCA TCTTCTATTT CAGTGAGTAA TTCATTGTCT AAAGCCTTTT 6120
Ref CACTCCCTGA CCCATCCCTT GCTTTGACAA GATTCTCTTT GATTGCAGCA TCTTCTATTT CAGTGAGTAA TTCATTGTCT AAAGCCTTTT 6120
                   6,140                6,160                6,180                6,200
AU  CGGATGTATT CGCTTTGAAA TTTGAGGCTT CCTCAACCTC ACCCGGTTTT TCTTGAATCT TCTCGGGACT CTCATCTTTC GATTCCATGT 6210
Ref CGGATGTATT CGCTTTGAAA TTTGAGGCTT CCTCAACCTC ACCCGGTTTT TCTTGAATCT TCTCGGGACT CTCATCTTTC GATTCCATGT 6210
        6,220                6,240                6,260                6,280                6,300
AU  CATTCATTTC ATCTCCTTGG ACCTCAGAAG GCAGTGCAGA TGTTGTGATA TGAAGGTGAT CAGTTCCAAC TTCCAACACA GGTTTGGCCT 6300
Ref CATTCATTTC ATCTCCTTGG ACCTCAGAAG GCAGTGCAGA TGTTGTGATA TGAAGGTGAT CAGTTCCAAC TTCCAACACA GGTTTGGCCT 6300
                   6,320                6,340                6,360                6,380
AU  CCTTTATCTC ATCCTTTTCT GTGCTTATCT CTTCCACTGT CTTTACTCCA TCAATTTCTC CAATTTTCGT TTCTTCTTCC TTCATATTCT 6390
Ref CCTTTATCTC ATCCTTTTCT GTGCTTATCT CTTCCACTGT CTTTACTCCA TCAATTTCTC CAATTTTCGT TTCTTCTTCC TTCATATTCT 6390
        6,400                6,420                6,440                6,460                6,480
AU  CTTCTTCAGG ATTGAGAATC TTCTCCTCAG GTTTCTCTGT TGGAATAGTC TGGAACGAAG TATTATTTTC TGCTGCAAGA GCTTCATCCT 6480
Ref CTTCTTCAGG ATTGAGAATC TTCTCCTCAG GTTTCTCTGT TGGAATAGTC TGGAACGAAG TATTATTTTC TGCTGCAAGA GCTTCATCCT 6480
                   6,500                6,520                6,540                6,560
AU  CCTGAAGCAG TTCCTTCTGT AAGATCACAT CTTCTCCATT ATCTTGTGAG AATTCTGCTT CGCCTTTTTT CTCACTTTCT ATCTTCTCAG 6570
Ref CCTGAAGCAG TTCCTTCTGT AAGATCACAT CTTCTCCATT ATCTTGTGAG AATTCTGCTT CGCCTTTTTT CTCACTTTCT ATCTTCTCAG 6570
        6,580                6,600                6,620                6,640                6,660
AU  CTGTACCAGC AGTTGCTTCG TCTTCTGCAA AAGCTTCTAG CTTGACAGAG CTGTCCTTTT GTGAGCATAT CTCTGCTGTT GTCCTTGCAT 6660
Ref CTGTACCAGC AGTTGCTTCG TCTTCTGCAA AAGCTTCTAG CTTGACAGAG CTGTCCTTTT GTGAGCATAT CTCTGCTGTT GTCCTTGCAT 6660
                   6,680                6,700                6,720                6,740
AU  CTGAAGCATC TTCTTCAGTC CCATCTTTTA TGATTTGCCT ATCGTTTGTT TTCTCATCTT CTTTCTCCTT TTTTTCATGC TCTAGCATTT 6750
Ref CTGAAGCATC TTCTTCAGTC CCATCTTTTA TGATTTGCCT ATCGTTTGTT TTCTCATCTT CTTTCTCCTT TTTTTCATGC TCTAGCATTT 6750
```

```
6,760 6,780 6,800 6,820 6,840
AU  CACTAGTTAT AGCAGTGGCT GAAATATAAA GTTGCTTGTC TGAGGAAGAC TGATCAGAAG CAATTTCTTC TTTTTTAACT AACTGAAGCT 6840
Ref CACTAGTTAT AGCAGTGGCT GAAATATAAA GTTGCTTGTC TGAGGAAGAC TGATCAGAAG CAATTTCTTC TTTTTTAACT AACTGAAGCT 6840
6,860 6,880 6,900 6,920
AU  CATTTAGCTC TTCTGTTACT CCAGCTGATG AGTCTTCATG GTTCCCATTG TGAAGCTTTT CAGTTTCTTT CCCTTTTTCT TTGTTTGCTT 6930
Ref CATTTAGCTC TTCTGTTACT CCAGCTGATG AGTCTTCATG GTTCCCATTG TGAAGCTTTT CAGTTTCTTT CCCTTTTTCT TTGTTTGCTT 6930
6,940 6,960 6,980 7,000 7,020
AU  CAATCTCTTG TGATTCCTCA GTTAAGGAAG CCTGAGCAAT AATGGTTTCT TGATCTATAA CAGCTTTAGG CTCCTGAGGT CCTTCCTTTT 7020
Ref CAATCTCTTG TGATTCCTCA GTTAAGGAAG CCTGAGCAAT AATGGTTTCT TGATCTATAA CAGCTTTAGG CTCCTGAGGT CCTTCCTTTT 7020
7,040 7,060 7,080 7,100
AU  CGAGAAGTTC CACTTCCTTC ACCTTTTCCC CCTCTTCTTC ATTGGCCTCA GCTCCATGCT TTAGGTTCTC TTCAGTCAAT TCTGAGGAAG 7110
Ref CGAGAAGTTC CACTTCCTTC ACCTTTTCCC CCTCTTCTTC ATTGGCCTCA GCTCCATGCT TTAGGTTCTC TTCAGTCAAT TCTGAGGAAG 7110
7,120 7,140 7,160 7,180 7,200
AU  ACTGACCAGA AGCAATTTCT TCTCTAGTAA CTAATTGAAG CTCATTTAGC TTTTCTGTTT CTCCAGCTGA CGAGTCTTCA AATTTCCCAT 7200
Ref ACTGACCAGA AGCAATTTCT TCTCTAGTAA CTAATTGAAG CTCATTTAGC TTTTCTGTTT CTCCAGCTGA CGAGTCTTCA AATTTCCCAT 7200
7,220 7,240 7,260 7,280
AU  CGTCAAGCTT TTTGGCTTCT TTCCTTTTCT CTTCGTTTGC TTCAATCTTT TGTGATTCCT CTGTTATTGA AGCCTGAACT ATAATGGTTT 7290
Ref CGTCAAGCTT TTTGGCTTCT TTCCTTTTCT CTTCGTTTGC TTCAATCTTT TGTGATTCCT CTGTTATTGA AGCCTGAACT ATAATGGTTT 7290
7,300 7,320 7,340 7,360 7,380
AU  CTTGATCCAT AACAGCTTCA GGCTCCTGAG GTCTTTCCTT TTCGAGAATT TCCACTTCCT TCACCTTTTC TTCCTCTTCT TCACTGGCCT 7380
Ref CTTGATCCAT AACAGCTTCA GGCTCCTGAG GTCTTTCCTT TTCGAGAATT TCCACTTCCT TCACCTTTTC TTCCTCTTCT TCACTGGCCT 7380
7,400 7,420 7,440 7,460
AU  CAGCTCCACG CTTTAGGTTC TCAACAGTCA ATTCTGAAGG CAAAGATTGC TCACTTGATT CCTCCACTTG GGGACATTGA GCAGTGGCAG 7470
Ref CAGCTCCACG CTTTAGGTTC TCAACAGTCA ATTCTGAAGG CAAAGATTGC TCACTTGATT CCTCCACTTG GGGACATTGA GCAGTGGCAG 7470
7,480 7,500 7,520 7,540 7,560
AU  CTTCATCTTT CTTGGCATCG TCAAGCTCCT GGATTGCTTC TTTTTCTGAA CATGTCTCTG CTACTTCCTT TGCATTAAAA GAATCTTCTG 7560
Ref CTTCATCTTT CTTGGCATCG TCAAGCTCCT GGATTGCTTC TTTTTCTGAA CATGTCTCTG CTACTTCCTT TGCATTAAAA GAATCTTCTG 7560
7,580 7,600 7,620 7,640
AU  AGATATTCTC TGAAGTGTCT GCTTCACTTA ATTTATCTTC TTCTATCTTC TTAACCGAGC TTACATTTTC TTTCTCCTCC TTAGAAACCA 7650
Ref AGATATTCTC TGAAGTGTCT GCTTCACTTA ATTTATCTTC TTCTATCTTC TTAACCGAGC TTACATTTTC TTTCTCCTCC TTAGAAACCA 7650
7,660 7,680 7,700 7,720 7,740
AU  ATGGAAAGCT TGTGGTCCCC TCCTTTGGTA TTTTAGTCTC AAGAATCTGC TCACCAGTTA CTTCTTCCAA CTTTTCCACA CTTAGGCCAT 7740
Ref ATGGAAAGCT TGTGGTCCCC TCCTTTGGTA TTTTAGTCTC AAGAATCTGC TCACCAGTTA CTTCTTCCAA CTTTTCCACA CTTAGGCCAT 7740
7,760 7,780 7,800 7,820
AU  TCTCGGCTTC AACAATCTGA TCTTTGGGCA TGTAAAATGC CTTGAGATCT CTCTCGGTAA TCTCTGCTTC ATCATTGTTG TTAGTTGGGG 7830
Ref TCTCGGCTTC AACAATCTGA TCTTTGGGCA TGTAAAATGC CTTGAGATCT CTCTCGGTAA TCTCTGCTTC ATCATTGTTG TTAGTTGGGG 7830
7,840 7,860 7,880 7,900 7,920
AU  CTTCTGGTGC CAAGTTGGAG GATCTTTCTT CAAGCTGCAC CTTCTCTATT TCTTTGGTCT CTTGATTAGA ATCACTTTCA AGGGTTTCTA 7920
Ref CTTCTGGTGC CAAGTTGGAG GATCTTTCTT CAAGCTGCAC CTTCTCTATT TCTTTGGTCT CTTGATTAGA ATCACTTTCA AGGGTTTCTA 7920
7,940 7,960 7,980 8,000
AU  AACTCATTTT GACTGAACTG ATATTTGCTG TATTTTCAAG CTCCTCATTC ACCTCAGTGT TGTTTAAGCT TGTCCCTGCT ATTACTTCTG 8010
Ref AACTCATTTT GACTGAACTG ATATTTGCTG TATTTTCAAG CTCCTCATTC ACCTCAGTGT TGTTTAAGCT TGTCCCTGCT ATTACTTCTG 8010
8,020 8,040 8,060 8,080 8,100
AU  TCACAGCTTC AATAGGACTC TCTTCATGAC AAAATTTTGA GTCATCAATA ATCTCCTCGA TTTTTTCTTC TTCTCTGTCT TCACCGTGCT 8100
Ref TCACAGCTTC AATAGGACTC TCTTCATGAC AAAATTTTGA GTCATCAATA ATCTCCTCGA TTTTTTCTTC TTCTCTGTCT TCACCGTGCT 8100
8,120 8,140 8,160 8,180
AU  CCTTTATAGG GTCTCTGGAA TCTTCGTCCT GTCTCCTTTC TTCTATTTCT TTCGTAACGC CGGATCCTTC TTTGTCGTTG GTCGAATTTT 8190
Ref CCTTTATAGG GTCTCTGGAA TCTTCGTCCT GTCTCCTTTC TTCTATTTCT TTCGTAACGC CGGATCCTTC TTTGTCGTTG GTCGAATTTT 8190
8,200 8,220 8,240 8,260 8,280
AU  CTTCAACCTC TGCTTTTATC TGCTCTTTGG ACGCAGCTAC CTTAATACAT TCAGTTGAGA CTGCACTGTA GTCTTTGACC TGTCAATACG 8280
Ref CTTCAACCTC TGCTTTTATC TGCTCTTTGG ACGCAGCTAC CTTAATACAT TCAGTTGAGA CTGCACTGTA GTCTTTGACC TGTCAATACG 8280
8,300 8,320 8,340 8,360
AU  TAGTTAAGAT GTTTGAGAAT TTGACATATC AAAAGAGCTA TTTGAGTATA AGTATTAATT GTGAATCACT TATCTAACCT CATTAGTTTC 8370
Ref TAGTTAAGAT GTTTGAGAAT TTGACATATC AAAAGAGCTA TTTGAGTATA AGTATTAATT GTGAATCACT TATCTAACCT CATTAGTTTC 8370
8,380 8,400 8,420 8,440 8,460
AU  TTCATCTCCT TTTGATACCG GATGAAGTTC ATTTGTGCTA CTTTCTTCCT CCATGACATG TTCTTTAATC TCTTTAATAC CATCGACTTT 8460
Ref TTCATCTCCT TTTGATACCG GATGAAGTTC ATTTGTGCTA CTTTCTTCCT CCATGACATG TTCTTTAATC TCTTTAATAC CATCGACTTT 8460
8,480 8,500 8,520 8,540
AU  CTCAACTTCC TTATCAACCT GCCAAAAATG AATATTTTTC AGTATAACTA AGAAATCTCC TTAAATAATC ATGTAGGCAG AATATTCAGA 8550
Ref CTCAACTTCC TTATCAACCT GCCAAAAATG AATATTTTTC AGTATAACTA AGAAATCTCC TTAAATAATC ATGTAGGCAG AATATTCAGA 8550
8,560 8,580 8,600 8,620 8,640
AU  AGTGTTCTTT ACCTCTTCAG ACAAAATTAC AGCCTTCGTA ACTACGTTTG TGTTTTGTTC ACTGCTGATG CTGTCTTCAT CTTCCAGTTT 8640
Ref AGTGTTCTTT ACCTCTTCAG ACAAAATTAC AGCCTTCGTA ACTACGTTTG TGTTTTGTTC ACTGCTGATG CTGTCTTCAT CTTCCAGTTT 8640
8,660 8,680 8,700 8,720
AU  GTCTTTCAGT TCCTCTTTTA TCTGTTCTTC AGTTTCTTGA TTCTGAACGC CAATGCTGAT TTCATCATCT AGGTTTTTGG TGTGTTCAGG 8730
Ref GTCTTTCAGT TCCTCTTTTA TCTGTTCTTC AGTTTCTTGA TTCTGAACGC CAATGCTGAT TTCATCATCT AGGTTTTTGG TGTGTTCAGG 8730
8,740 8,760 8,780 8,800 8,820
AU  GATTGTTTCC TTACTTTGAT CTTCAGCCAC TGCTTCTGTT TCAGATTTTG ATTTTTCTTC AAAACCTGCT ATTGCTACAT TCTGTTTGAA 8820
Ref GATTGTTTCC TTACTTTGAT CTTCAGCCAC TGCTTCTGTT TCAGATTTTG ATTTTTCTTC AAAACCTGCT ATTGCTACAT TCTGTTTGAA 8820
8,840 8,860 8,880 8,900
AU  ATTGATACAT TAGTTGAGGG CTGAACAAAC TCAGCTACTT AACTAAAGAG AAACAGTTGA GGTGATATGA ACATTTGGAC AAGGTTAGAC 8910
Ref ATTGATACAT TAGTTGAGGG CTGAACAAAC TCAGCTACTT AACTAAAGAG AAACAGTTGA GGTGATATGA ACATTTGGAC AAGGTTAGAC 8910
8,920 8,940 8,960 8,980 9,000
AU  ATTATATACA ACTTTGTTTT CCATTATGGT ATATATATAT AATTTTGGTT TTTGTCTTCT TCAATGATTT GATTTCATGA CGTCCTTCAA 9000
Ref ATTATATACA ACTTTGTTTT CCATTATGGT ATATATATAT AATTTTGGTT TTTGTCTTCT TCAATGATTT GATTTCATGA CGTCCTTCAA 9000
```

```
                                9,020                          9,040                          9,060                          9,080
AU  CGAGTTATCA CATTTTTGTG CTAAAACCTG GAATCTCTTT TGTAATATGA ACCGATTCAT GGAATCGACT AGAAAGTTGC ACACTTATAC 9090
Ref CGAGTTATCA CATTTTTGTG CTAAAACCTG GAATCTCTTT TGTAATATGA ACCGATTCAT GGAATCGACT AGAAAGTTGC ACACTTATAC 9090
                     9,100                          9,120                          9,140                          9,160                          9,180
AU  TTCCTATCTG CGTAAAATAT TATGAAAGGT TAAAGGAGGA TTCAATGGTA GGCTAACCTC TTCTGTTACT GATGTGGTTT CAATCTTGAC 9180
Ref TTCCTATCTG CGTAAAATAT TATGAAAGGT TAAAGGAGGA TTCAATGGTA GGCTAACCTC TTCTGTTACT GATGTGGTTT CAATCTTGAC 9180
                                9,200                          9,220                          9,240                          9,260
AU  AACTGAAGCT TGGCCTGGAT CCTCAACAGT TTTATCTTCA ATCTGGTTTT CAATTATCTC GTTTCCACTG GTCTTTTGAG TTTCCGCAAT 9270
Ref AACTGAAGCT TGGCCTGGAT CCTCAACAGT TTTATCTTCA ATCTGGTTTT CAATTATCTC GTTTCCACTG GTCTTTTGAG TTTCCGCAAT 9270
                     9,280                          9,300                          9,320                          9,340                          9,360
AU  GAGCTGTTAA GGACAAAGCT TCAGTACAGA TATTGAAAAG AAAGAATATG TAAGATGAAC GAAGTTTAGT TTTCATTTCC TGACCTCTTC 9360
Ref GAGCTGTTAA GGACAAAGCT TCAGTACAGA TATTGAAAAG AAAGAATATG TAAGATGAAC GAAGTTTAGT TTTCATTTCC TGACCTCTTC 9360
                                9,380                          9,400                          9,420                          9,440
AU  ATGTTTCTTG TCTTCAACCA GTTTATCCTT CAGCTCTTCT CCAGAGCCAT CTTTGATCAT CTCTTCCAAA TGGGAAGTTG AATAAGATAT 9450
Ref ATGTTTCTTG TCTTCAACCA GTTTATCCTT CAGCTCTTCT CCAGAGCCAT CTTTGATCAT CTCTTCCAAA TGGGAAGTTG AATAAGATAT 9450
                     9,460                          9,480                          9,500                          9,520                          9,540
AU  TTCAGTAACT TCCTTGTTCC CCATAACCTG TCAATTTTCT CTCTCATTTA ATAGTTTTCA CAATAATAAA TTTTAATTGC AAGGAAGTAC 9540
Ref TTCAGTAACT TCCTTGTTCC CCATAACCTG TCAATTTTCT CTCTCATTTA ATAGTTTTCA CAATAATAAA TTTTAATTGC AAGGAAGTAC 9540
                                9,560                          9,580                          9,600                          9,620
AU  GTGAAATTGT TAAGGTTTTG AGCTAGTTTT GCCTACCTCT TTGTCCAAAT CTTTATCCAC CGTGATTTCA TTTCCTTCAA TTGTTTTCTT 9630
Ref GTGAAATTGT TAAGGTTTTG AGCTAGTTTT GCCTACCTCT TTGTCCAAAT CTTTATCCAC CGTGATTTCA TTTCCTTCAA TTGTTTTCTT 9630
                     9,640                          9,660                          9,680                          9,700                          9,720
AU  GCATTGATGT TCTTCACCCT CTGTCTCATC TCTTGCTATG ACAATTTTCT TTTCGTTGGC AGCATCTTCT GTTTCAGTGA GTAATTCATT 9720
Ref GCATTGATGT TCTTCACCCT CTGTCTCATC TCTTGCTATG ACAATTTTCT TTTCGTTGGC AGCATCTTCT GTTTCAGTGA GTAATTCATT 9720
                                9,740                          9,760                          9,780                          9,800
AU  GTCAGAAGCC CTTTTACATG TCTCCTTGTT GAAATTTAAG ACTTCCTCTA TCTCTCTTGT TTGTTCTTGA ATATTCTCTG TACTTTCTTC 9810
Ref GTCAGAAGCC CTTTTACATG TCTCCTTGTT GAAATTTAAG ACTTCCTCTA TCTCTCTTGT TTGTTCTTGA ATATTCTCTG TACTTTCTTC 9810
                     9,820                          9,840                          9,860                          9,880                          9,900
AU  TTCTTTCAGT TCTGTTTCCA TTGTTTCATT TTCTTTGACC TCAAAAGGCA ATGTAGAGGT TGTAGAATGA AGGTTATCTG TTGATGTCTC 9900
Ref TTCTTTCAGT TCTGTTTCCA TTGTTTCATT TTCTTTGACC TCAAAAGGCA ATGTAGAGGT TGTAGAATGA AGGTTATCTG TTGATGTCTC 9900
                                9,920                          9,940                          9,960                          9,980
AU  ACCTGGAACA ATATGAACTG CAGCTGCTCT ATCTTCCAAG GCATCTTGAA GCTCTTTTGT GCTCTTTGAT TGTGAGCTTT CTTCATCTTC 9990
Ref ACCTGGAACA ATATGAACTG CAGCTGCTCT ATCTTCCAAG GCATCTTGAA GCTCTTTTGT GCTCTTTGAT TGTGAGCTTT CTTCATCTTC 9990
                     10,000                         10,020                         10,040                         10,060                         10,080
AU  AAATGATTCT GCTTTTATTT TCTGTTCTTC TATAACAGCT CCATGCTCCT CCGATTCCTT GGATATAACC TCACTAATAA TTGTTTCACC 10080
Ref AAATGATTCT GCTTTTATTT TCTGTTCTTC TATAACAGCT CCATGCTCCT CCGATTCCTT GGATATAACC TCACTAATAA TTGTTTCACC 10080
                                10,100                         10,120                         10,140                         10,160
AU  TTCCAACATG GCTTCAGGTT CATTTGTGCT ACTTTCTTCC TCCATGACCT GTTCTTTAGT ATCTTCACCA CCATCAGCCT GCTAAAGTTA 10170
Ref TTCCAACATG GCTTCAGGTT CATTTGTGCT ACTTTCTTCC TCCATGACCT GTTCTTTAGT ATCTTCACCA CCATCAGCCT GCTAAAGTTA 10170
                     10,180                         10,200                         10,220                         10,240                         10,260
AU  ACTTTTGTCA ATCAACAGAA ATAAATCTTC TTAAAAAGTC ATATAGGCAA AGTATTTATA AGTGTTCTTT ACCTCTCTAG ACACAACCTT 10260
Ref ACTTTTGTCA ATCAACAGAA ATAAATCTTC TTAAAAAGTC ATATAGGCAA AGTATTTATA AGTGTTCTTT ACCTCTCTAG ACACAACCTT 10260
                                10,280                         10,300                         10,320                         10,340
AU  ATTTGTGTTT TTGTCTTTGT TAACACTGTC TTCATCCTTA AGTTTGTCTT TCTGTTCCTC TTTTATTTGA TTTTCAGTTT CTTTATTTTG 10350
Ref ATTTGTGTTT TTGTCTTTGT TAACACTGTC TTCATCCTTA AGTTTGTCTT TCTGTTCCTC TTTTATTTGA TTTTCAGTTT CTTTATTTTG 10350
                     10,360                         10,380                         10,400                         10,420                         10,440
AU  GGCATCGGTG TTGGTTTCGT CATCTTGGTT CTTGGTGTCT TCAGGGATTG GTTCCTTGCT TTGGTCTTCA GCCACTGGTT CTGAGTCAGA 10440
Ref GGCATCGGTG TTGGTTTCGT CATCTTGGTT CTTGGTGTCT TCAGGGATTG GTTCCTTGCT TTGGTCTTCA GCCACTGGTT CTGAGTCAGA 10440
                                10,460                         10,480                         10,500                         10,520
AU  TTCGGGCTTT TCTTCAGAAC ATGCTAGTGC TAGCTCAACA TTCTGTATGA AATTAATAAG TTAGCCGAGG GCCCCAGAAA TCCACTAAAA 10530
Ref TTCGGGCTTT TCTTCAGAAC ATGCTAGTGC TAGCTCAACA TTCTGTATGA AATTAATAAG TTAGCCGAGG GCCCCAGAAA TCCACTAAAA 10530
                     10,540                         10,560                         10,580                         10,600                         10,620
AU  TGAAGAGAAA CAATTGAATG GTGATATGAA CATCTGGACA AGGTTAGACA CTTATAAAAT TTTGGTTTTT TTCTTCTTTC AATAATTTGA 10620
Ref TGAAGAGAAA CAATTGAATG GTGATATGAA CATCTGGACA AGGTTAGACA CTTATAAAAT TTTGGTTTTT TTCTTCTTTC AATAATTTGA 10620
                                10,640                         10,660                         10,680                         10,700
AU  TTTTATGAGG TTCTTCAATG GGTTAACTGT GTTCTATACT AAAACCTGAA ATTCCTTTTG TCACTCACAC AGATTCATAA AATTAACTAG 10710
Ref TTTTATGAGG TTCTTCAATG GGTTAACTGT GTTCTATACT AAAACCTGAA ATTCCTTTTG TCACTCACAC AGATTCATAA AATTAACTAG 10710
                     10,720                         10,740                         10,760                         10,780                         10,800
AU  ACTGTTGCAC AATCTGTTAC GGTTACATTT GCATAAAATA TTATGAAAAG CATGGAAGGA GGATTCAATG CTGTCTAACC TCTTCTGTTG 10800
Ref ACTGTTGCAC AATCTGTTAC GGTTACATTT GCATAAAATA TTATGAAAAG CATGGAAGGA GGATTCAATG CTGTCTAACC TCTTCTGTTG 10800
                                10,820                         10,840                         10,860                         10,880
AU  CTGTTGCGGT TTCAATCCTG GCATCTGAAG CTTGGACTGG ATCCTCAACA GTTTTATCTT CTATTGGATT CTCATTTTCA CTGGTCTTTT 10890
Ref CTGTTGCGGT TTCAATCCTG GCATCTGAAG CTTGGACTGG ATCCTCAACA GTTTTATCTT CTATTGGATT CTCATTTTCA CTGGTCTTTT 10890
                     10,900                         10,920                         10,940                         10,960                         10,980
AU  GAGCTTCCAA GATGACCTGT TATAGATTAA AGCTTAACTA CAGATATCAA AAGGCAAAAA ATGAGTAAGA TGAAGAAAAG TTTAGTATTG 10980
Ref GAGCTTCCAA GATGACCTGT TATAGATTAA AGCTTAACTA CAGATATCAA AAGGCAAAAA ATGAGTAAGA TGAAGAAAAG TTTAGTATTG 10980
                                11,000                         11,020                         11,040                         11,060
AU  CTTACCTTTT CTGTTGAAAC TTCATTCAAT ATGATTTCCC CTGTCTCCGT TCTTGCTTTG ACAAGTTTCT TGTGGATTGC AGCATCCTCT 11070
Ref CTTACCTTTT CTGTTGAAAC TTCATTCAAT ATGATTTCCC CTGTCTCCGT TCTTGCTTTG ACAAGTTTCT TGTGGATTGC AGCATCCTCT 11070
                     11,080                         11,100                         11,120                         11,140                         11,160
AU  GTTTCATTTA GTAACTCATT TTCGGAAGCC TTTTCATGTA TCTCCTTATT GAAATCGGAA GCTTCCTCAA TCTCCCTTGT TTGTTCTGGA 11160
Ref GTTTCATTTA GTAACTCATT TTCGGAAGCC TTTTCATGTA TCTCCTTATT GAAATCGGAA GCTTCCTCAA TCTCCCTTGT TTGTTCTGGA 11160
                                11,180                         11,200                         11,220                         11,240
AU  ATCTTGTCGG TATTCTCATC TTCTTCCAGT TCTGTTTCCT TCCTTTCATC TCCTTGGACC TCAGATGGCA GCATAGCGGT TGTAAGATGA 11250
Ref ATCTTGTCGG TATTCTCATC TTCTTCCAGT TCTGTTTCCT TCCTTTCATC TCCTTGGACC TCAGATGGCA GCATAGCGGT TGTAAGATGA 11250
```

11,260 11,280 11,300 11,320 11,340

AU ATGTGATCTG TTGGCAATGT GTCAGCAGGA TTTGGAAGAT CTTTGTGTTT TTCTGTCTGG ATCACTTCTG AAGAAGCATT GTCATCTACT 11340

Ref ATGTGATCTG TTGGCAATGT GTCAGCAGGA TTTGGAAGAT CTTTGTGTTT TTCTGTCTGG ATCACTTCTG AAGAAGCATT GTCATCTACT 11340

11,360 11,380 11,400 11,420

AU GCAAGAGCTT CATCCTCTTG AAGCCGGTCC TTCTGCAAGG ATGCATCTTC TTCATTATAA TCTTGTGTTT TCACACTTTC TATCATCTCA 11430

Ref GCAAGAGCTT CATCCTCTTG AAGCCGGTCC TTCTGCAAGG ATGCATCTTC TTCATTATAA TCTTGTGTTT TCACACTTTC TATCATCTCA 11430

11,440 11,460 11,480 11,500 11,520

AU TCTGTTATAG TTCTTTCACA TTCCTCTTCC TTTGAAGGCA GTGCAGAGCT TGGATTCTGA ATCTGTTCAT CTGATGTCTT CTCAGTGAGA 11520

Ref TCTGTTATAG TTCTTTCACA TTCCTCTTCC TTTGAAGGCA GTGCAGAGCT TGGATTCTGA ATCTGTTCAT CTGATGTCTT CTCAGTGAGA 11520

11,540 11,560 11,580 11,600

AU GTCTCACTGA CTGTCATTTC ATCTTCTGCA ACAGCTTCTG GTTTGAAAGA CCTTTCGGTT TGTGAGCATA TCTTTAATGC TGTCTTTTCC 11610

Ref GTCTCACTGA CTGTCATTTC ATCTTCTGCA ACAGCTTCTG GTTTGAAAGA CCTTTCGGTT TGTGAGCATA TCTTTAATGC TGTCTTTTCC 11610

11,620 11,640 11,660 11,680 11,700

AU TCCTTAGCAT CTTCTTGAAT CTCGTTTCTT ATGAGCTCCC CTTCGTTTGT TTTCTCATCT TCTTTTTCGG TGTCATCATT TTTACCATCA 11700

Ref TCCTTAGCAT CTTCTTGAAT CTCGTTTCTT ATGAGCTCCC CTTCGTTTGT TTTCTCATCT TCTTTTTCGG TGTCATCATT TTTACCATCA 11700

11,720 11,740 11,760 11,780

AU TCAAGCTCGA GAAATTCTTC TTTTTCCAGG CATATCTCTG TCGTTGTATC TGCAACTGAA AGATCTTCTG AAATCTTGTC TGGAATCATG 11790

Ref TCAAGCTCGA GAAATTCTTC TTTTTCCAGG CATATCTCTG TCGTTGTATC TGCAACTGAA AGATCTTCTG AAATCTTGTC TGGAATCATG 11790

11,800 11,820 11,840 11,860 11,880

AU TCTGCTTCAT ATATTTTCTC TTCTTCTATG CGTATATTTT CTTCATTGTC TTCCTCAAAA ACCAATGGAG TGCTTATAGG GCCCTCCTTT 11880

Ref TCTGCTTCAT ATATTTTCTC TTCTTCTATG CGTATATTTT CTTCATTGTC TTCCTCAAAA ACCAATGGAG TGCTTATAGG GCCCTCCTTT 11880

11,900 11,920 11,940 11,960

AU GGTTTATTAC CTGAGAGAGT CTGCTCACCA GTTACTACAC CTCCTGTAAG AACATCTGGT TTAGTGGGTA CTTCTCCCTT TGTTTTTCCA 11970

Ref GGTTTATTAC CTGAGAGAGT CTGCTCACCA GTTACTACAC CTCCTGTAAG AACATCTGGT TTAGTGGGTA CTTCTCCCTT TGTTTTTCCA 11970

11,980 12,000 12,020 12,040 12,060

AU TATTCTATCG CCTCACTGAT GTCTCTGGTT CCATGCTCAC TTTCCTCAGA AGGTAATGTT GAAGATGTAG TTTGAATTTG CTCCACTGGT 12060

Ref TATTCTATCG CCTCACTGAT GTCTCTGGTT CCATGCTCAC TTTCCTCAGA AGGTAATGTT GAAGATGTAG TTTGAATTTG CTCCACTGGT 12060

12,080 12,100 12,120 12,140

AU CCATCAGTTA GAGAAGCCTG ATCAGCAATA GTTTCTTCTT CTGAGACTGC TTTAAGTTCT GTTTGTTCAT TTGACAAGCA TACCTCTTCT 12150

Ref CCATCAGTTA GAGAAGCCTG ATCAGCAATA GTTTCTTCTT CTGAGACTGC TTTAAGTTCT GTTTGTTCAT TTGACAAGCA TACCTCTTCT 12150

12,160 12,180 12,200 12,220 12,240

AU ATTGTCTTTG TTTCAGATGA CTCTCCTCGA CACTCTTGTT TTATTTCATC ATCCTTCTGC ATCTCTTCTT CGATCTTTTC AACAGGACTT 12240

Ref ATTGTCTTTG TTTCAGATGA CTCTCCTCGA CACTCTTGTT TTATTTCATC ATCCTTCTGC ATCTCTTCTT CGATCTTTTC AACAGGACTT 12240

12,260 12,280 12,300 12,320

AU GCTTCAGTCT CAGAAAGCAA TGAGCAATAA GAAGTTTGAA TTTGCTCCTC AAGTTTTACT TCTAAAACAG TTTTAGGGGA ACCCATATGA 12330

Ref GCTTCAGTCT CAGAAAGCAA TGAGCAATAA GAAGTTTGAA TTTGCTCCTC AAGTTTTACT TCTAAAACAG TTTTAGGGGA ACCCATATGA 12330

12,340 12,360 12,380 12,400 12,420

AU TCTATTTCCC CCAGTAGCTC AGCTTTTGAA ATTTTGGAGG CTTCACTCTT TGTCTCTACA AGCTCATGGT CCTGGGTTAG GTTGCCAACC 12420

Ref TCTATTTCCC CCAGTAGCTC AGCTTTTGAA ATTTTGGAGG CTTCACTCTT TGTCTCTACA AGCTCATGGT CCTGGGTTAG GTTGCCAACC 12420

12,440 12,460 12,480 12,500

AU GTTTCTTCTC TTACTTCTGT TACTGCTTCA ATACGACTCT CATTATAAGA CAAGTTTGAG TCATCAATTA TCTCCTCAGT TTTCTCTTCT 12510

Ref GTTTCTTCTC TTACTTCTGT TACTGCTTCA ATACGACTCT CATTATAAGA CAAGTTTGAG TCATCAATTA TCTCCTCAGT TTTCTCTTCT 12510

12,520 12,540 12,560 12,580 12,600

AU GCTTCTCGAT CTTCGCTATG CAGACTTACA GGATCTCTGG ACTCCTCATC CTGTCTCATT TCCTCAAATT CTTTGGTGAT GTGGGATGCT 12600

Ref GCTTCTCGAT CTTCGCTATG CAGACTTACA GGATCTCTGG ACTCCTCATC CTGTCTCATT TCCTCAAATT CTTTGGTGAT GTGGGATGCT 12600

12,620 12,640 12,660 12,680

AU TCTCCATCAC TGGTTAAATA TTCTTCAACC TGCAAATTTT CTGCGACTAT CTGCTCTTTG GTCTCGACTT CCGTGACACA TTCAGTTAGG 12690

Ref TCTCCATCAC TGGTTAAATA TTCTTCAACC TGCAAATTTT CTGCGACTAT CTGCTCTTTG GTCTCGACTT CCGTGACACA TTCAGTTAGG 12690

12,700 12,720 12,740 12,760 12,780

AU ACTGAACTGT AGTCATCCAC CTGTGCATAA GGTAGTTAAA AAGTTTAACA ATATGACATC AAAAATGTTA TTTGAGTAAC TATTCATTTT 12780

Ref ACTGAACTGT AGTCATCCAC CTGTGCATAA GGTAGTTAAA AAGTTTAACA ATATGACATC AAAAATGTTA TTTGAGTAAC TATTCATTTT 12780

12,800 12,820 12,840 12,860

AU GAACCTGAAA AGTGTACTTA TTCACTAACG TTTGATACTG ATTTTCATAT AATTTACTTG ATTTTGTTTT TTAAGAAGAA AGAGAGTGAA 12870

Ref GAACCTGAAA AGTGTACTTA TTCACTAACG TTTGATACTG ATTTTCATAT AATTTACTTG ATTTTGTTTT TTAAGAAGAA AGAGAGTGAA 12870

12,880 12,900 12,920 12,940 12,960

AU AAGAGTAGAA TAATTGTTCA CTAACCTTTG CACTATAATT CGTGATAACC ATCTCAGGAA TCTGATTTTC TTTTTCCATT TCAGTCTCTT 12960

Ref AAGAGTAGAA TAATTGTTCA CTAACCTTTG CACTATAATT CGTGATAACC ATCTCAGGAA TCTGATTTTC TTTTTCCATT TCAGTCTCTT 12960

12,980 13,000 13,020 13,040

AU CTTTCAAGCT TTCCGGTTTT TCTATGTTCT TAGCAAGATT CTCTTCTCCT GTGGCCTTTT TGTCATCCTT GCTAGTACCC AAGGTTGCTT 13050

Ref CTTTCAAGCT TTCCGGTTTT TCTATGTTCT TAGCAAGATT CTCTTCTCCT GTGGCCTTTT TGTCATCCTT GCTAGTACCC AAGGTTGCTT 13050

13,060 13,080 13,100 13,120 13,140

AU CCAAATCCAT GGCTCCATCC AATGGCTTTC CATCTTCAAA TTTTGATCTT TCCAGTCCTA CATCTGATGA ATTTTCAACT GGCAGTGTTT 13140

Ref CCAAATCCAT GGCTCCATCC AATGGCTTTC CATCTTCAAA TTTTGATCTT TCCAGTCCTA CATCTGATGA ATTTTCAACT GGCAGTGTTT 13140

13,160 13,180 13,200 13,220

AU CCGCTTCGCA TGCGTCGGTT TGATTCTCTA GTTCTCTATG TTCTTCACCA CTAGAGTCAT CGGTGACCTC TAGATTAGAT TTCTCCGCCA 13230

Ref CCGCTTCGCA TGCGTCGGTT TGATTCTCTA GTTCTCTATG TTCTTCACCA CTAGAGTCAT CGGTGACCTC TAGATTAGAT TTCTCCGCCA 13230

13,240 13,260 13,280 13,300 13,320

AU ACTGTTCCTC GACTTCATGT CTACTCTCAT TTCCTGTATC TTCAGGCTTA GCTTTAGGGA CATCTACTAG TGGATTATCT TCAGTTAGAA 13320

Ref ACTGTTCCTC GACTTCATGT CTACTCTCAT TTCCTGTATC TTCAGGCTTA GCTTTAGGGA CATCTACTAG TGGATTATCT TCAGTTAGAA 13320

13,340 13,360 13,380 13,400

AU TTGATTTTTC ATCCGTTATT TGTGGCACAG GAGTTTCTGA GTGCTCACTG GAACCCTGCT CTTTTTCTGA AGTTTCATCA GTTCTTTTCC 13410

Ref TTGATTTTTC ATCCGTTATT TGTGGCACAG GAGTTTCTGA GTGCTCACTG GAACCCTGCT CTTTTTCTGA AGTTTCATCA GTTCTTTTCC 13410

13,420 13,440 13,460 13,480 13,500

AU TTTCTTCAAT GTGTGTGGCC TCATATTCCT TCACTTCGTC TCCTAATATT TCTTTCAAGG GAATCTCCTT GGTGGTCATC TCCAATTGAT 13500

Ref TTTCTTCAAT GTGTGTGGCC TCATATTCCT TCACTTCGTC TCCTAATATT TCTTTCAAGG GAATCTCCTT GGTGGTCATC TCCAATTGAT 13500

```
                       13,520                        13,540                        13,560                        13,580
AU  CTTCACTTCC CGCATTTGGA ACTTTATCCT GCATTGCATC TTCTTCTTTC CCCATGTCCT TCTCGTGTGT TACGTGAGAG TCCCCTGATT 13590
Ref CTTCACTTCC CGCATTTGGA ACTTTATCCT GCATTGCATC TTCTTCTTTC CCCATGTCCT TCTCGTGTGT TACGTGAGAG TCCCCTGATT 13590
            13,600                        13,620                        13,640                        13,660                        13,680
AU  TCACATCAAG TTCAGCACTG CATGCACCAC TCTGTTCATG TTCAATTCTT GCTAAGCTGT TCTCTTCGAT GACAACAACA TTATTTATTT 13680
Ref TCACATCAAG TTCAGCACTG CATGCACCAC TCTGTTCATG TTCAATTCTT GCTAAGCTGT TCTCTTCGAT GACAACAACA TTATTTATTT 13680
                       13,700                        13,720                        13,740                        13,760
AU  CGTTCTTTCC CTTTTCGACT TCAGCATGAG CTTCAGTTTC CTTTTTTTCT TCTGTTTCTG CTACACATGA TGCCGAGGTT TCTTCTTCAA 13770
Ref CGTTCTTTCC CTTTTCGACT TCAGCATGAG CTTCAGTTTC CTTTTTTTCT TCTGTTTCTG CTACACATGA TGCCGAGGTT TCTTCTTCAA 13770
            13,780                        13,800                        13,820                        13,840                        13,860
AU  TGTCCTTTTT GGACTCCTTG AGCTCAAGCG ACAAACTACA GACATGGTCT TCTTTCACTT CTATTGGTGT TGTTTCTTCT TTTGACGCCT 13860
Ref TGTCCTTTTT GGACTCCTTG AGCTCAAGCG ACAAACTACA GACATGGTCT TCTTTCACTT CTATTGGTGT TGTTTCTTCT TTTGACGCCT 13860
                       13,880                        13,900                        13,920                        13,940
AU  GTATAATATC AAACAGACTA GAATCATTAT TTGGCTTTGC TGTTTCCTCT ATGGGCGCTG TTTCCTAGAT GATTTAGTTA CTAGCCTTTA 13950
Ref GTATAATATC AAACAGACTA GAATCATTAT TTGGCTTTGC TGTTTCCTCT ATGGGCGCTG TTTCCTAGAT GATTTAGTTA CTAGCCTTTA 13950
            13,960                        13,980                        14,000                        14,020                        14,040
AU  AAACAATTGG ATATATATAT TGATGGAAGA CTGAGTTCAG AAACATTGAA TAAGTGGAAA AGTTAAGATG TTAGTGAGTC ATACTATCTA 14040
Ref AAACAATTGG ATATATATAT TGATGGAAGA CTGAGTTCAG AAACATTGAA TAAGTGGAAA AGTTAAGATG TTAGTGAGTC ATACTATCTA 14040
                       14,060                        14,080                        14,100                        14,120
AU  CTTACCTCTG TTATCGACAC CACTGCTGCA TTCTTCTCTT CTTCAGCTTT TTCTTTCATT GTATCCGTCA TTAGTGATGT CTGATCTTTC 14130
Ref CTTACCTCTG TTATCGACAC CACTGCTGCA TTCTTCTCTT CTTCAGCTTT TTCTTTCATT GTATCCGTCA TTAGTGATGT CTGATCTTTC 14130
            14,140                        14,160                        14,180                        14,200                        14,220
AU  TTGTTATCTT CCTCATCTAG AATTTGTTTT TCTGTATTCT CAACGGTGCT GTCTGGTTCC AGATTTTCAG AGACCTGAAT AAAAAGTAAT 14220
Ref TTGTTATCTT CCTCATCTAG AATTTGTTTT TCTGTATTCT CAACGGTGCT GTCTGGTTCC AGATTTTCAG AGACCTGAAT AAAAAGTAAT 14220
                       14,240                        14,260                        14,280                        14,300
AU  ATTGGGAGAT TGGTGAAACG TGGAGGTTTT CAGCTTCCAA CTTGATCTTA CGACTTACAA AGAAAAAAAA AAACTTTATT TTAAGAACTA 14310
Ref ATTGGGAGAT TGGTGAAACG TGGAGGTTTT CAGCTTCCAA CTTGATCTTA CGACTTACAA AGAAAAAAAA AAACTTTATT TTAAGAACTA 14310
            14,320                        14,340                        14,360                        14,380                        14,400
AU  GGCTCTTCAA AATTTTAGAA GTAACATGAA TTTCACCTCT ACTTCAGGTG TGGCTTCATT TTCATTTTCT TCTGTAGCTT TCAGTGAGTC 14400
Ref GGCTCTTCAA AATTTTAGAA GTAACATGAA TTTCACCTCT ACTTCAGGTG TGGCTTCATT TTCATTTTCT TCTGTAGCTT TCAGTGAGTC 14400
                       14,420                        14,440                        14,460                        14,480
AU  ATTGATGATA GCACATTCTT CCTTCACGAT CAATGTTGGG CCTTCACAAA CCTGCATGAA GAATAAGGTG TACTTTAATT AATAATCTCT 14490
Ref ATTGATGATA GCACATTCTT CCTTCACGAT CAATGTTGGG CCTTCACAAA CCTGCATGAA GAATAAGGTG TACTTTAATT AATAATCTCT 14490
            14,500                        14,520                        14,540                        14,560                        14,580
AU  GAAAGAGATT AAAATTGTTT TAAAAAAAGG TTTTAAACTG AGTTAGTATT CATTCATGCC CTAAATTTAG ACCTCTTTGG TTTCTTCCTG 14580
Ref GAAAGAGATT AAAATTGTTT TAAAAAAAGG TTTTAAACTG AGTTAGTATT CATTCATGCC CTAAATTTAG ACCTCTTTGG TTTCTTCCTG 14580
                       14,600                        14,620                        14,640                        14,660
AU  ATTTAAGTTA TCTTCTACTT TCTCCATGGC ATCCTTTGGT AGTGCACTTC TGTTCTCTGA CTCCTGCAGT ATACTCTCTT CTTTGTGTTC 14670
Ref ATTTAAGTTA TCTTCTACTT TCTCCATGGC ATCCTTTGGT AGTGCACTTC TGTTCTCTGA CTCCTGCAGT ATACTCTCTT CTTTGTGTTC 14670
            14,680                        14,700                        14,720                        14,740                        14,760
AU  TAAAGCTTCA TTTTCCACTG GTTCAACATC GGAGAACTTG TACTGCTTTT CGTGCTCATT ATCCATTAGT TGTCCACCGA ATGAGACAGT 14760
Ref TAAAGCTTCA TTTTCCACTG GTTCAACATC GGAGAACTTG TACTGCTTTT CGTGCTCATT ATCCATTAGT TGTCCACCGA ATGAGACAGT 14760
                       14,780                        14,800                        14,820                        14,840
AU  AACATGTAAA TCATCAGCAT TCTCGTTTGG AATCTCAACT TCCACGGTTT CATTACTCTG ACTTGCTTTA GTTTGTAGGT GGTCAGTTGG 14850
Ref AACATGTAAA TCATCAGCAT TCTCGTTTGG AATCTCAACT TCCACGGTTT CATTACTCTG ACTTGCTTTA GTTTGTAGGT GGTCAGTTGG 14850
            14,860                        14,880                        14,900                        14,920                        14,940
AU  TGAGTCTTCC CTCGTTTGTC CCTCGGATCT CTTGTCATGG TCTTTGTCCG ATGTTGCTTC TTCAATGCTT GTGTTTGTTT CTGCAGTTTC 14940
Ref TGAGTCTTCC CTCGTTTGTC CCTCGGATCT CTTGTCATGG TCTTTGTCCG ATGTTGCTTC TTCAATGCTT GTGTTTGTTT CTGCAGTTTC 14940
                       14,960                        14,980                        15,000                        15,020
AU  GGCACAGATG GCAGTATTGA CATCCTCAGA AACTGCATCT AAAGGACACA CATGGTTCTC CTTTTTGGGT TCATTCTTTT GCTTGAAGCT 15030
Ref GGCACAGATG GCAGTATTGA CATCCTCAGA AACTGCATCT AAAGGACACA CATGGTTCTC CTTTTTGGGT TCATTCTTTT GCTTGAAGCT 15030
            15,040                        15,060                        15,080                        15,100                        15,120
AU  CTCCTGGATT TCGTCCTTGT GGGAATTTTC CTCTTGATTC TTCTGTACGA CACCAATATT TTCTACTTCA CTAGTTTTTA CTTCAGGCAT 15120
Ref CTCCTGGATT TCGTCCTTGT GGGAATTTTC CTCTTGATTC TTCTGTACGA CACCAATATT TTCTACTTCA CTAGTTTTTA CTTCAGGCAT 15120
                       15,140                        15,160                        15,180                        15,200
AU  TGGAACAATG GATTCCTTGT CCTCTCTCAT TTCAGACAAT CCCTTTGTTA TGGCCAGCTC CTTGTCTGTC ACCTGTATGA CTCTCACATC 15210
Ref TGGAACAATG GATTCCTTGT CCTCTCTCAT TTCAGACAAT CCCTTTGTTA TGGCCAGCTC CTTGTCTGTC ACCTGTATGA CTCTCACATC 15210
            15,220                        15,240                        15,260                        15,280                        15,300
AU  CACCGCAACG TTTTCTGAGT GCTTGTGTTG ATCTTGATCT TCTTCTGAAG TGTCCTCGGC TATTTTCCTA TCTTCAATGA GAGTCGTATC 15300
Ref CACCGCAACG TTTTCTGAGT GCTTGTGTTG ATCTTGATCT TCTTCTGAAG TGTCCTCGGC TATTTTCCTA TCTTCAATGA GAGTCGTATC 15300
                       15,320                        15,340                        15,360                        15,380
AU  ATATTTTTCC AAAGGCATAG AAGAATGTTC CTTAACATCA TCTCCTAATA CCTCTTTCAA CAGAATCTCC TTGGAGGTCA TTTCCATTTG 15390
Ref ATATTTTTCC AAAGGCATAG AAGAATGTTC CTTAACATCA TCTCCTAATA CCTCTTTCAA CAGAATCTCC TTGGAGGTCA TTTCCATTTG 15390
            15,400                        15,420                        15,440                        15,460                        15,480
AU  ATTTTCACTT CCTGTGTTTA GAAATTTATC CTGCACTCCA TCTTCTTCTC GGGAAATCTC GATGTCCTTC TCACTTGTCA CATATGATTC 15480
Ref ATTTTCACTT CCTGTGTTTA GAAATTTATC CTGCACTCCA TCTTCTTCTC GGGAAATCTC GATGTCCTTC TCACTTGTCA CATATGATTC 15480
                       15,500                        15,520                        15,540                        15,560
AU  TACTGATTTT ACCTCAATTT CTGCACTTTC TAAACCAATC CCTTCATCTT CACCATCAAC AACATTATTT ATTTCATTCA TTTCCTCTTC 15570
Ref TACTGATTTT ACCTCAATTT CTGCACTTTC TAAACCAATC CCTTCATCTT CACCATCAAC AACATTATTT ATTTCATTCA TTTCCTCTTC 15570
            15,580                        15,600                        15,620                        15,640                        15,660
AU  TTCTGGTTCA GCTTTAGCTT CCTTTAAGTT TGTTTTTACC GTTTCCGCTG TTTGTGATGT GCAGTCCAAA TCAGCAGTAT TGATTTGTTC 15660
Ref TTCTGGTTCA GCTTTAGCTT CCTTTAAGTT TGTTTTTACC GTTTCCGCTG TTTGTGATGT GCAGTCCAAA TCAGCAGTAT TGATTTGTTC 15660
                       15,680                        15,700                        15,720                        15,740
AU  GTTTACCCCT GTTTCTTCTT CAGTATCCAT TTTGGACTCC TCGAGCTCAA ATGAAGAGCT GCAGACAAGT TCTTCTTTGA ATTTGATTAC 15750
Ref GTTTACCCCT GTTTCTTCTT CAGTATCCAT TTTGGACTCC TCGAGCTCAA ATGAAGAGCT GCAGACAAGT TCTTCTTTGA ATTTGATTAC 15750
```

```
                15,760                        15,780                        15,800                        15,820                        15,840
AU   AGTAATTTCT TTTTCTGCTG TACCTGAAGC TGCAGTTTCT GGTCCTCTAC ACAGCTGCAG GATATACAAA ATTTCAATAT AACTCAGACA 15840
Ref  AGTAATTTCT TTTTCTGCTG TACCTGAAGC TGCAGTTTCT GGTCCTCTAC ACAGCTGCAG GATATACAAA ATTTCAATAT AACTCAGACA 15840
                           15,860                        15,880                        15,900                        15,920
AU   AAGTTCTGGA TGGTTATGGA CTTATAAGAC AAATTCCAAT GGGCCATGGG TCGTCTTTGT GCTAAGTTCA TGAACTGAAC TTTTTATTCT 15930
Ref  AAGTTCTGGA TGGTTATGGA CTTATAAGAC AAATTCCAAT GGGCCATGGG TCGTCTTTGT GCTAAGTTCA TGAACTGAAC TTTTTATTCT 15930
                15,940                        15,960                        15,980                        16,000                        16,020
AU   TTGAAATCCA AGTTATCGTG AGGTTTGCTA CTAGCATCAG TTGCCTCATG ACACCTCTCT TATTAAGGAT CTCTATTTTT GGCCTTATCT 16020
Ref  TTGAAATCCA AGTTATCGTG AGGTTTGCTA CTAGCATCAG TTGCCTCATG ACACCTCTCT TATTAAGGAT CTCTATTTTT GGCCTTATCT 16020
                           16,040                        16,060                        16,080                        16,100
AU   TTTCTGGTTT TGATCAGAAT TCACTCAGCT CTATATTTTT ACACTGTGTA ATGGATAGCC CATCACTTCT GATTGTATTG TTTTGCATTG 16110
Ref  TTTCTGGTTT TGATCAGAAT TCACTCAGCT CTATATTTTT ACACTGTGTA ATGGATAGCC CATCACTTCT GATTGTATTG TTTTGCATTG 16110
                16,120                        16,140                        16,160                        16,180                        16,200
AU   TACGCATGTG TTTGCCAATA ATAATGGTTG TTTGTTCCAG TTGTAATTTT CTACCTCTTC AATTGTAATC TGATTATTGT TGGTGTCTTC 16200
Ref  TACGCATGTG TTTGCCAATA ATAATGGTTG TTTGTTCCAG TTGTAATTTT CTACCTCTTC AATTGTAATC TGATTATTGT TGGTGTCTTC 16200
                           16,220                        16,240                        16,260                        16,280
AU   CTCCTCTAGA ATTTGTTTTC CATCATTCTC AACTGTGTTG TCCGGTTCTA GCTTTTCAGC GACCTGAATG AAAATATATT CATAGATTGG 16290
Ref  CTCCTCTAGA ATTTGTTTTC CATCATTCTC AACTGTGTTG TCCGGTTCTA GCTTTTCAGC GACCTGAATG AAAATATATT CATAGATTGG 16290
                16,300                        16,320                        16,340                        16,360                        16,380
AU   TTGAACATGG AGGTTTCAGA TTTTAGCAAA TAGATTGAAA TTGAGTTAGT ATTCATGATA GGAACTCTTT TGATTACCTC AGCTTCATCC 16380
Ref  TTGAACATGG AGGTTTCAGA TTTTAGCAAA TAGATTGAAA TTGAGTTAGT ATTCATGATA GGAACTCTTT TGATTACCTC AGCTTCATCC 16380
                           16,400                        16,420                        16,440                        16,460
AU   TTGTCCACAG TTTTCATATT TAGATTCTGC TTTTGCACTT CATCATTTTC ATCAGTTCGG ACATCAGGAT CCGATTCAGA GGATATCTCT 16470
Ref  TTGTCCACAG TTTTCATATT TAGATTCTGC TTTTGCACTT CATCATTTTC ATCAGTTCGG ACATCAGGAT CCGATTCAGA GGATATCTCT 16470
                16,480                        16,500                        16,520                        16,540                        16,560
AU   TCAAGCTGCA CAATCTCCAC TTCTGTCTCT TGAGCATCAC TCTTAATATT TTCTACAGTC TTCTCGGCTG AGCTTATATT TGATGCATTT 16560
Ref  TCAAGCTGCA CAATCTCCAC TTCTGTCTCT TGAGCATCAC TCTTAATATT TTCTACAGTC TTCTCGGCTG AGCTTATATT TGATGCATTT 16560
                           16,580                        16,600                        16,620                        16,640
AU   ACAATCTTGT CGGTGTCGTT CAAGCTCGTC TCTGCTGTTA CTTCTAGTAC AGCTTCAAGA CGACTCTCCT CATGACACGG TTTCGAGTCA 16650
Ref  ACAATCTTGT CGGTGTCGTT CAAGCTCGTC TCTGCTGTTA CTTCTAGTAC AGCTTCAAGA CGACTCTCCT CATGACACGG TTTCGAGTCA 16650
                16,660                        16,680                        16,700                        16,720                        16,740
AU   TCAATAATCT CCTTGGTTTT CTCTTCTGCT TCTCGATCTT TGCTACGCAG ACTTACAGGA TCTCCAGACT CTTCAGCCTG ACTCATTTCT 16740
Ref  TCAATAATCT CCTTGGTTTT CTCTTCTGCT TCTCGATCTT TGCTACGCAG ACTTACAGGA TCTCCAGACT CTTCAGCCTG ACTCATTTCT 16740
                           16,760                        16,780                        16,800                        16,820
AU   TCTAATTCTT TGGTGATGTG GGATGATTCT ACATCATTGG TTGAATGTTC TTTGACCTCC AAGTTTTCTG CAACTATCTG CTCTTTGGAC 16830
Ref  TCTAATTCTT TGGTGATGTG GGATGATTCT ACATCATTGG TTGAATGTTC TTTGACCTCC AAGTTTTCTG CAACTATCTG CTCTTTGGAC 16830
                16,840                        16,860                        16,880                        16,900                        16,920
AU   TCTGCTTCCA TGAAACATTC AGTTGAGACA GAACTGTAGT CTTCCACCTG CGCATAACAC AGATAAAATG TTTAACAATA TAACATCCGA 16920
Ref  TCTGCTTCCA TGAAACATTC AGTTGAGACA GAACTGTAGT CTTCCACCTG CGCATAACAC AGATAAAATG TTTAACAATA TAACATCCGA 16920
                           16,940                        16,960                        16,980                        17,000
AU   AAATCTGTTT GAGTATCTAT TCATTTTGAA AGTGAAAAGT GTAATTATTC ACTAACGTTC GATACCCATT TTCATATCTT CAATTTGTTT 17010
Ref  AAATCTGTTT GAGTATCTAT TCATTTTGAA AGTGAAAAGT GTAATTATTC ACTAACGTTC GATACCCATT TTCATATCTT CAATTTGTTT 17010
                17,020                        17,040                        17,060                        17,080                        17,100
AU   TTGTTTTTTA ACAAGAAAAA GAGCAGAATA AGTATTCACT AACCTTTGCA TTATAAGTCA TGGTAACCAT CTCAGGAATC TGATTTTCTT 17100
Ref  TTGTTTTTTA ACAAGAAAAA GAGCAGAATA AGTATTCACT AACCTTTGCA TTATAAGTCA TGGTAACCAT CTCAGGAATC TGATTTTCTT 17100
                           17,120                        17,140                        17,160                        17,180
AU   TTTCTATTTC AGTATCTTCC TTCAAGCTTT CCGGTTTCTC CATGTTCCTT GCTAATTTCT CTTCTTCAGT AGCCTTCTCA TCATCCTTGT 17190
Ref  TTTCTATTTC AGTATCTTCC TTCAAGCTTT CCGGTTTCTC CATGTTCCTT GCTAATTTCT CTTCTTCAGT AGCCTTCTCA TCATCCTTGT 17190
                17,200                        17,220                        17,240                        17,260                        17,280
AU   GAGTACCCAA GGTTGCTTCC AAATCCACAG CTTCATCCAA TGGCTTTCCA TCTTCAATTT TTGATCTATC CAGTCCTACA TCTGATGGAT 17280
Ref  GAGTACCCAA GGTTGCTTCC AAATCCACAG CTTCATCCAA TGGCTTTCCA TCTTCAATTT TTGATCTATC CAGTCCTACA TCTGATGGAT 17280
                           17,300                        17,320                        17,340                        17,360
AU   TTTCAACTGG CAGTGCTTTT GCTTCGCAAG CATTGGTTTG ACTCTCGAGT TCTCTATGTT CTTCAACAGT AGAGACATCG GTGACCTCTA 17370
Ref  TTTCAACTGG CAGTGCTTTT GCTTCGCAAG CATTGGTTTG ACTCTCGAGT TCTCTATGTT CTTCAACAGT AGAGACATCG GTGACCTCTA 17370
                17,380                        17,400                        17,420                        17,440                        17,460
AU   GATTAGATTC CTTGGCCAAC TGTTCCTCGA CTACTTGTCC ACTTCCGTTT CCCGTATCTT TAGACACAGC TTTAGGGACA TCTACTAGCG 17460
Ref  GATTAGATTC CTTGGCCAAC TGTTCCTCGA CTACTTGTCC ACTTCCGTTT CCCGTATCTT TAGACACAGC TTTAGGGACA TCTACTAGCG 17460
                           17,480                        17,500                        17,520                        17,540
AU   GATTATCTTC ACTTTGTATT AATTTCTCAT CCGTTATTTG TGGCATAGGA GTTTCTGAGT GCTCACGCTG ATCCTGATCT TTGTCTGAAG 17550
Ref  GATTATCTTC ACTTTGTATT AATTTCTCAT CCGTTATTTG TGGCATAGGA GTTTCTGAGT GCTCACGCTG ATCCTGATCT TTGTCTGAAG 17550
                17,560                        17,580                        17,600                        17,620                        17,640
AU   TTTCGTCAGT TATTTTCCTT TCTTCAGAGA ATGTTGCCTC AGATTCCTTC ACTTCATCTC CTAATGTTTC TTTCAAGGGA ATCTCCTTGG 17640
Ref  TTTCGTCAGT TATTTTCCTT TCTTCAGAGA ATGTTGCCTC AGATTCCTTC ACTTCATCTC CTAATGTTTC TTTCAAGGGA ATCTCCTTGG 17640
                           17,660                        17,680                        17,700                        17,720
AU   TGGTCATCTC CAATTGATCT TCACTTCCTG CATATGGAAC TTTATCCTGC ACTGTATCTT CTTGTTCGGA ATCTCCATGT CCTTCTCGTG 17730
Ref  TGGTCATCTC CAATTGATCT TCACTTCCTG CATATGGAAC TTTATCCTGC ACTGTATCTT CTTGTTCGGA ATCTCCATGT CCTTCTCGTG 17730
                17,740                        17,760                        17,780                        17,800                        17,820
AU   TGTTACGTAC GAGTCCCCTG ATTTCACATC AATTTCAGCA CTGCATACAC CACTCTCTTC ATGTTCAGTT CTTGCTGAGC AGTTCTCTTT 17820
Ref  TGTTACGTAC GAGTCCCCTG ATTTCACATC AATTTCAGCA CTGCATACAC CACTCTCTTC ATGTTCAGTT CTTGCTGAGC AGTTCTCTTT 17820
                           17,840                        17,860                        17,880                        17,900
AU   GATGACAACA ACATTATAGA TTTTGTTCTT TGTCTTTTCT CTTTCAGCTT CAGCT----- ---------- ---TCAGCAT CAGCATCAGC 17892
Ref  GATGACAACA ACATTATAGA TTTTGTTCTT TGTCTTTTCT CTTTCAGCTT CAGCTTCAGC ATCAGCATCA GCTTCAGCAT CAGCATCAGC 17910
                17,920                        17,940                        17,960                        17,980                        18,000
AU   TTCATCTTCA GCTTCAGCAT CAGCATCAGC TTCAGCTTCA GCTTCCTTTA TGTCTGTTTC TTCTGTTTCT GCTGGATATG ATGCCGAGAT 17982
Ref  TTCATCTTCA GCTTCAGCAT CAGCATCAGC TTCAGCTTCA GCTTCCTTTA TGTCTGTTTC TTCTGTTTCT GCTGGATATG ATGCCGAGAT 18000
```

```
                                     18,020                        18,040                        18,060                        18,080
AU  TTCTTCTTTG GTGTCGTTTT TGGACTCCTT GAGCTCAAAC TCCGAACTAC GAGCCTGGTC TTCTTTCACT TCTATTGGTG TTATTTCTTC 18072
Ref TTCTTCTTTG GTGTCGTTTT TGGACTCCTT GAGCTCAAAC TCCGAACTAC GAGCCTGGTC TTCTTTCACT TCTATTGGTG TTATTTCTTC 18090
                   18,100                        18,120                        18,140                        18,160                    18,180
AU  TTTTGACTCC TGTACAATAT CATACAGACT AGAATTGTTA TTTTGCTTTG CTGTTTCCTC TATGGACACT GTTTCCTCAA TGATTGTGTG 18162
Ref TTTTGACTCC TGTACAATAT CATACAGACT AGAATTGTTA TTTTGCTTTG CTGTTTCCTC TATGGACACT GTTTCCTCAA TGATTGTGTG 18180
                                     18,200                        18,220                        18,240                        18,260
AU  GCTCGTCTGT AAAACATTGA ATATATAAGT TGATGGAAGA TTGAATTCAG AGCCATTGAA TAAGTGGAAA AGTTAAGATG TTAGTGATTC 18252
Ref GCTCGTCTGT AAAACATTGA ATATATAAGT TGATGGAAGA TTGAATTCAG AGCCATTGAA TAAGTGGAAA AGTTAAGATG TTAGTGATTC 18270
                   18,280                        18,300                        18,320                        18,340                    18,360
AU  ATACAATCTA CTTACCACTG TTATCGACAC CACTGCTGTA GTATTCTCTT CCTTAGCTTT TTCTTTCTTC GTCTCAGTCA TTAGTGTTGT 18342
Ref ATACAATCTA CTTACCACTG TTATCGACAC CACTGCTGTA GTATTCTCTT CCTTAGCTTT TTCTTTCTTC GTCTCAGTCA TTAGTGTTGT 18360
                                     18,380                        18,400                        18,420                        18,440
AU  GTGATCCTTC TTGTTGTCTT CCTCCTCTAG AAATTGTTTT TCAGCATTCG CTACTGTGTT GTCTGGTTCC AGCTTTTCAC AGACCTGAAC 18432
Ref GTGATCCTTC TTGTTGTCTT CCTCCTCTAG AAATTGTTTT TCAGCATTCG CTACTGTGTT GTCTGGTTCC AGCTTTTCAC AGACCTGAAC 18450
                   18,460                        18,480                        18,500                        18,520                    18,540
AU  AAAAATAGTA TCAAAAGATT GGTGAAACAT GGAGGTTTTC AGCTTTCAAC TTGATCTTCA GACTTTCAAA ACAAACAAAA AAAATTTCTT 18522
Ref AAAAATAGTA TCAAAAGATT GGTGAAACAT GGAGGTTTTC AGCTTTCAAC TTGATCTTCA GACTTTCAAA ACAAACAAAA AAAATTTCTT 18540
                                     18,560                        18,580                        18,600                        18,620
AU  AAGAACTAGT CTCTGCAAAA TTTTAGAAGT AACATGACTG TCACCTCTAC TTCAGGTGAT GCTTCATTTT CATTTTCTTC TGTAGCTTTC 18612
Ref AAGAACTAGT CTCTGCAAAA TTTTAGAAGT AACATGACTG TCACCTCTAC TTCAGGTGAT GCTTCATTTT CATTTTCTTC TGTAGCTTTC 18630
                   18,640                        18,660                        18,680                        18,700                    18,720
AU  AGTGAGTCAT CAATGATAGT ACATTCTTCC TTCACAATCG ACATTGGACC TTCATGAACC TGCATGAAGA ATAAGGTGTA CTTTTATTAA 18702
Ref AGTGAGTCAT CAATGATAGT ACATTCTTCC TTCACAATCG ACATTGGACC TTCATGAACC TGCATGAAGA ATAAGGTGTA CTTTTATTAA 18720
                                     18,740                        18,760                        18,780                        18,800
AU  TAATTACAAT GATAGTATCT GAAAGAGATT AAAATTGTTT TAAAAAAAAA AGTTTTAAAC TGAGTTAGTA TTCATTCATG CCCTAAATTT 18792
Ref TAATTACAAT GATAGTATCT GAAAGAGATT AAAATTGTTT TAAAAAAAAA AGTTTTAAAC TGAGTTAGTA TTCATTCATG CCCTAAATTT 18810
                   18,820                        18,840                        18,860                        18,880                    18,900
AU  ACGCCTCTTT GGTTTCTTCC TGTTTTAAGT TCTCCATGGC ATCCTTTGGT TCATGTTTCT GTGCACTGCA GTTCTCCGAC TGCTGCAGTA 18882
Ref ACGCCTCTTT GGTTTCTTCC TGTTTTAAGT TCTCCATGGC ATCCTTTGGT TCATGTTTCT GTGCACTGCA GTTCTCCGAC TGCTGCAGTA 18900
                                     18,920                        18,940                        18,960                        18,980
AU  TACTCTCTTC TTTGTGTTCT AAAGCTTCAT TTTCCACTGG TCGAACACTG AGGAGCTCAT ACTGCTTTTC GTGCTCATTG TCCACTAGTT 18972
Ref TACTCTCTTC TTTGTGTTCT AAAGCTTCAT TTTCCACTGG TCGAACACTG AGGAGCTCAT ACTGCTTTTC GTGCTCATTG TCCACTAGTT 18990
                   19,000                        19,020                        19,040                        19,060                    19,080
AU  GTCCAACATA TGAATTCATA ACATGTAAAT CATCAGCCTT TTCGTTTGAA ATCTCAACTT CCACGGTTTC ATTAATGTCA CTTGCTTTAG 19062
Ref GTCCAACATA TGAATTCATA ACATGTAAAT CATCAGCCTT TTCGTTTGAA ATCTCAACTT CCACGGTTTC ATTAATGTCA CTTGCTTTAG 19080
                                     19,100                        19,120                        19,140                        19,160
AU  TTTGTAGGTG GTCAGTTGGT GAGTCTTCGC TTGTTTCTCC CTCAGATCTC TCGTCATGGT CTTTGACTGA TGTTGCTTCT TCGATGCTTG 19152
Ref TTTGTAGGTG GTCAGTTGGT GAGTCTTCGC TTGTTTCTCC CTCAGATCTC TCGTCATGGT CTTTGACTGA TGTTGCTTCT TCGATGCTTG 19170
                   19,180                        19,200                        19,220                        19,240                    19,260
AU  TGCTTGTTTC TGCAGTTTTG GCACAGATGG CAGTATTGAC ATCCTCAGAC ACTGCATCCA AAGGACACGC ATGGTTCTCC TCTTCGGGTT 19242
Ref TGCTTGTTTC TGCAGTTTTG GCACAGATGG CAGTATTGAC ATCCTCAGAC ACTGCATCCA AAGGACACGC ATGGTTCTCC TCTTCGGGTT 19260
                                     19,280                        19,300                        19,320                        19,340
AU  CATTCTTTTG CTTGAAGCTC TCCTCCTGGA TTTCGTCCCT GTGGGAATTT TCCTCCTGAT TCTTCTGTAT GAAACTGATA TTTTCTACTT 19332
Ref CATTCTTTTG CTTGAAGCTC TCCTCCTGGA TTTCGTCCCT GTGGGAATTT TCCTCCTGAT TCTTCTGTAT GAAACTGATA TTTTCTACTT 19350
                   19,360                        19,380                        19,400                        19,420                    19,440
AU  CATTAGTTTT TACTTCAGGC ATTGGAACAA TGGATTCGTC GTCCTTTCTC ATTTCGTCTG ATTCCTTTGT TATAGCCAGC TCCTCATCTG 19422
Ref CATTAGTTTT TACTTCAGGC ATTGGAACAA TGGATTCGTC GTCCTTTCTC ATTTCGTCTG ATTCCTTTGT TATAGCCAGC TCCTCATCTG 19440
                                     19,460                        19,480                        19,500                        19,520
AU  TCTCCTGTAC AACTTTTGCG TCCACCATAT TTTCAGGGTG TTTGTATAGA GCTTGATCTT CTTCTGAAGT TTCATCAACT ATTTTCTTCT 19512
Ref TCTCCTGTAC AACTTTTGCG TCCACCATAT TTTCAGGGTG TTTGTATAGA GCTTGATCTT CTTCTGAAGT TTCATCAACT ATTTTCTTCT 19530
                   19,540                        19,560                        19,580                        19,600                    19,620
AU  CTTCAATGAG AGCTGCATCA TTTTCTTCTG AAGGCATAGT CGAACCTTCC TTAACATCAT CTCCTGATAC CTCTTTCAAT GGAATCTCCT 19602
Ref CTTCAATGAG AGCTGCATCA TTTTCTTCTG AAGGCATAGT CGAACCTTCC TTAACATCAT CTCCTGATAC CTCTTTCAAT GGAATCTCCT 19620
                                     19,640                        19,660                        19,680                        19,700
AU  CAATGGTCAT TTCCATTTGA TTTTCACTTC CTGCATTCGG CATTTTATCC TGCATTGTAT CTTCTTCTCG GGGAGTCTCG ATGTCCTCAC 19692
Ref CAATGGTCAT TTCCATTTGA TTTTCACTTC CTGCATTCGG CATTTTATCC TGCATTGTAT CTTCTTCTCG GGGAGTCTCG ATGTCCTCAC 19710
                   19,720                        19,740                        19,760                        19,780                    19,800
AU  TTGTCACACA CAATTCTTCG GATTTTACGT TAATGTCTGC ACTTTTTAAA CCAATCTCAT CATCTTCACC ATCAACAATA TTATTTATTT 19782
Ref TTGTCACACA CAATTCTTCG GATTTTACGT TAATGTCTGC ACTTTTTAAA CCAATCTCAT CATCTTCACC ATCAACAATA TTATTTATTT 19800
                                     19,820                        19,840                        19,860                        19,880
AU  CATTCTTTTC CTCTTCTGGT TCAGCTTTAG CTTCTTTTAA GTTTGTTTCT TCCGTTTCTG CTGCTTGTGA TGTGCAGTCC AACTCAGCAT 19872
Ref CATTCTTTTC CTCTTCTGGT TCAGCTTTAG CTTCTTTTAA GTTTGTTTCT TCCGTTTCTG CTGCTTGTGA TGTGCAGTCC AACTCAGCAT 19890
                   19,900                        19,920                        19,940                        19,960                    19,980
AU  TACTGATATA TTCTTTTACT GTGGCGGCTT CTTCAGTATC CATCTTGGAC TGCTCGAGCT CAGATGAAGA GCTACAGACA AGTTCTTCTT 19962
Ref TACTGATATA TTCTTTTACT GTGGCGGCTT CTTCAGTATC CATCTTGGAC TGCTCGAGCT CAGATGAAGA GCTACAGACA AGTTCTTCTT 19980
                                     20,000                        20,020                        20,040                        20,060
AU  TGACTTCAAT TGGAGTTATC TCTTCTTTTG CTGTACCTGA AGCTGCAGTT TCTGATCCTT TACACATCTG TGTGGTAAAC AAAATTTCAA 20052
Ref TGACTTCAAT TGGAGTTATC TCTTCTTTTG CTGTACCTGA AGCTGCAGTT TCTGATCCTT TACACATCTG TGTGGTAAAC AAAATTTCAA 20070
                   20,080                        20,100                        20,120                        20,140                    20,160
AU  CATAAACATG ATAAATTTCT GGACGATCAC GGACTATATT ATAAAACAAA TTCCAACAAG TCATGAGCCA TCTTTGTGCT AAGTTCATGA 20142
Ref CATAAACATG ATAAATTTCT GGACGATCAC GGACTATATT ATAAAACAAA TTCCAACAAG TCATGAGCCA TCTTTGTGCT AAGTTCATGA 20160
                                     20,180                        20,200                        20,220                        20,240
AU  ACGGAATTTT TTATTCTTTG AAATCCAAGT TATCTTGAGG TTTGCTAGCC GGCACCAGTT GCCTCATGAC ACCTCCCTTA TTAATGAACT 20232
Ref ACGGAATTTT TTATTCTTTG AAATCCAAGT TATCTTGAGG TTTGCTAGCC GGCACCAGTT GCCTCATGAC ACCTCCCTTA TTAATGAACT 20250
```

```
20,260 20,280 20,300 20,320 20,340
AU  CTGTTTTTGG CCTTTTCTGA TTTTGATCAG ATAACCTTTT GATGTGGCAT AAGACTTCTG GTTGTGCTAT TTTGCATTGT ACGCATGTGT 20322
Ref CTGTTTTTGG CCTTTTCTGA TTTTGATCAG ATAACCTTTT GATGTGGCAT AAGACTTCTG GTTGTGCTAT TTTGCATTGT ACGCATGTGT 20340
20,360 20,380 20,400 20,420
AU  TTGCCAATAA TGGTTATTTG TTCCATGTCT ATTAATGGAA GTAGTTCGGA ATTATATTAT AAGTTTGAAG TTGAAAGATT AGAGAGTTGT 20412
Ref TTGCCAATAA TGGTTATTTG TTCCATGTCT ATTAATGGAA GTAGTTCGGA ATTATATTAT AAGTTTGAAG TTGAAAGATT AGAGAGTTGT 20430
20,440 20,460 20,480 20,500 20,520
AU  ATAATTTTCT TACCTCTTCA ATTGGAATCT GATTATTGTA GGTGTCTTCC TTCTCTAGAA TTTGTTTTCC ATCATTCTCA GCTATGTTGT 20502
Ref ATAATTTTCT TACCTCTTCA ATTGGAATCT GATTATTGTA GGTGTCTTCC TTCTCTAGAA TTTGTTTTCC ATCATTCTCA GCTATGTTGT 20520
20,540 20,560 20,580 20,600
AU  CCGGTTCTAG CTTTTCAGTG ACCTGAATGG AAATATATTG GAAGATTGGT TAAGCATGGA GGTTTCTGCT TTTAGCAAAT AGATTGAAAT 20592
Ref CCGGTTCTAG CTTTTCAGTG ACCTGAATGG AAATATATTG GAAGATTGGT TAAGCATGGA GGTTTCTGCT TTTAGCAAAT AGATTGAAAT 20610
20,620 20,640 20,660 20,680 20,700
AU  TGGGTTAGTA TTCATGATAG GAACTGTTTT GATTACCTCA GCTTCATCCT TGTCCACAGT TTCCATATTT AGGTTCTGCT TTTGCACTTC 20682
Ref TGGGTTAGTA TTCATGATAG GAACTGTTTT GATTACCTCA GCTTCATCCT TGTCCACAGT TTCCATATTT AGGTTCTGCT TTTGCACTTC 20700
20,720 20,740 20,760 20,780
AU  ATCATTGTCA TTAGTTCGGA CATCAGGATC CAATTCCGAA GATATCTCTT CAAGCTGCAC ATTCTCTGCT TCTGTCTCTT GAGCATCACT 20772
Ref ATCATTGTCA TTAGTTCGGA CATCAGGATC CAATTCCGAA GATATCTCTT CAAGCTGCAC ATTCTCTGCT TCTGTCTCTT GAGCATCACT 20790
20,800 20,820 20,840 20,860 20,880
AU  CTTAATATTT CCTACAGTCG TCTCGGCTGA GCTGATATTT GATGCATTTA CAATCTTGTC GGTGTCGTTC AAGCTCGTCT CTGCTGTTAC 20862
Ref CTTAATATTT CCTACAGTCG TCTCGGCTGA GCTGATATTT GATGCATTTA CAATCTTGTC GGTGTCGTTC AAGCTCGTCT CTGCTGTTAC 20880
20,900 20,920 20,940 20,960
AU  TTCTGTTACA GCTTCAATAC AACTCTCCTC ATGACACGGT TTCGAGTCAT CAATAATCTC CTCGGTTTTC TCTTCTGCTT CTCGATCTTT 20952
Ref TTCTGTTACA GCTTCAATAC AACTCTCCTC ATGACACGGT TTCGAGTCAT CAATAATCTC CTCGGTTTTC TCTTCTGCTT CTCGATCTTT 20970
20,980 21,000 21,020 21,040 21,060
AU  GCTACACAGA CTTACAGGAT CTCCGGACTC TTCAGCCTGT CCCATTTCTT CTAATTCTTT GGTGATTTGG GATGCTTCTC CATCATTGGT 21042
Ref GCTACACAGA CTTACAGGAT CTCCGGACTC TTCAGCCTGT CCCATTTCTT CTAATTCTTT GGTGATTTGG GATGCTTCTC CATCATTGGT 21060
21,080 21,100 21,120 21,140
AU  TGAATGTTCT TTGACCTCCA AGTTTTCTGC AACTATCTGC TCTTTGGAAT CGGCTTCCAT GGAACATTCA GTTGAGACTG AACTATAGTC 21132
Ref TGAATGTTCT TTGACCTCCA AGTTTTCTGC AACTATCTGC TCTTTGGAAT CGGCTTCCAT GGAACATTCA GTTGAGACTG AACTATAGTC 21150
21,160 21,180 21,200 21,220 21,240
AU  TGCCACCTGC GCATAACATA GTTACAATGT TTAACAATAT AACATCCAAA ATCTGTTTTG AGTATCTATT CATTTTGAAA ATGAAAAGTG 21222
Ref TGCCACCTGC GCATAACATA GTTACAATGT TTAACAATAT AACATCCAAA ATCTGTTTTG AGTATCTATT CATTTTGAAA ATGAAAAGTG 21240
21,260 21,280 21,300 21,320
AU  TAATTATTCA GTAACGCTTG ATACCCTTTT TTATATCTTT AATTTGATTT TGTATTTTTA GAAGAAAAAG AGTGAAAAGA GTAGAATATC 21312
Ref TAATTATTCA GTAACGCTTG ATACCCTTTT TTATATCTTT AATTTGATTT TGTATTTTTA GAAGAAAAAG AGTGAAAAGA GTAGAATATC 21330
21,340 21,360 21,380 21,400 21,420
AU  TATTCACTAA CCTTTGCATT ATAAGTCATG GTAACCATCT CAGGAATCTG GTTTTCTTTT TCTTTTTCAG CATCTTCTTT CAAGCTTTCA 21402
Ref TATTCACTAA CCTTTGCATT ATAAGTCATG GTAACCATCT CAGGAATCTG GTTTTCTTTT TCTTTTTCAG CATCTTCTTT CAAGCTTTCA 21420
21,440 21,460 21,480 21,500
AU  GTTTTTTCTA TGTTCTTAGC TGAATTCTCT TCTTCAGTAG CCTTTTCGCC ATCCTTGCAA GTACCCAAGG TTGCTTCCAA ATCCATGGTT 21492
Ref GTTTTTTCTA TGTTCTTAGC TGAATTCTCT TCTTCAGTAG CCTTTTCGCC ATCCTTGCAA GTACCCAAGG TTGCTTCCAA ATCCATGGTT 21510
21,520 21,540 21,560 21,580 21,600
AU  TCATCCAGCG GCTTTCCATC TTCAAATTTC GACCTTTCTA GTCCTACATC TGATGAATTT TCTGCTGGCA GTGTTTTTTC TTTGCAAGCA 21582
Ref TCATCCAGCG GCTTTCCATC TTCAAATTTC GACCTTTCTA GTCCTACATC TGATGAATTT TCTGCTGGCA GTGTTTTTTC TTTGCAAGCA 21600
21,620 21,640 21,660 21,680
AU  TCGGTTTGAT TCTCTAGTTC TCTATGTTCT TCACCAGTAG AGACATTGGT GACATCTAGA TTAGATTCCT CAGCCAACTG TTCCTTGACT 21672
Ref TCGGTTTGAT TCTCTAGTTC TCTATGTTCT TCACCAGTAG AGACATTGGT GACATCTAGA TTAGATTCCT CAGCCAACTG TTCCTTGACT 21690
21,700 21,720 21,740 21,760 21,780
AU  TCATGTCTAC TCTCGTTTCC TGTATCTTCA GGCTCAGCTT TAGGGACATC TGCTAGCGGA TTATCTTCAT TTAGAATTAA TTTCTCATCC 21762
Ref TCATGTCTAC TCTCGTTTCC TGTATCTTCA GGCTCAGCTT TAGGGACATC TGCTAGCGGA TTATCTTCAT TTAGAATTAA TTTCTCATCC 21780
21,800 21,820 21,840 21,860
AU  GTTACTTGTG GCACTGGAGT TTCTGAGTGC TCACACAAAT CATGATCTTT TTCTGAAGTT TCATCAGTTA TTTTCCTTTC TTCAATGAGT 21852
Ref GTTACTTGTG GCACTGGAGT TTCTGAGTGC TCACACAAAT CATGATCTTT TTCTGAAGTT TCATCAGTTA TTTTCCTTTC TTCAATGAGT 21870
21,880 21,900 21,920 21,940 21,960
AU  GTGGCCTCAT ATTCTTTCAC TTCATCTCCT AGTATTTCTT TCAAGGGAAT CTCCTTGGTG GTCATCTCCA ATTGATCTTC ATTTTCTTCA 21942
Ref GTGGCCTCAT ATTCTTTCAC TTCATCTCCT AGTATTTCTT TCAAGGGAAT CTCCTTGGTG GTCATCTCCA ATTGATCTTC ATTTTCTTCA 21960
21,980 22,000 22,020 22,040
AU  TTGCGAACTT TATCCTGCAT TGCATCTTCT TCTTTGGGAA TCTCCATGTC CTTCTCATGT GTTACGTATG AGTCCCCTGA TTTCACATCA 22032
Ref TTGCGAACTT TATCCTGCAT TGCATCTTCT TCTTTGGGAA TCTCCATGTC CTTCTCATGT GTTACGTATG AGTCCCCTGA TTTCACATCA 22050
22,060 22,080 22,100 22,120 22,140
AU  ATTTCAGTAC TGCTTACACC AATCTGTTCA TGTTCTGTTC TTGCTGAGCT GTTCTCTTCG ATGACAACAA CATTACATTT TTGGTTCTTT 22122
Ref ATTTCAGTAC TGCTTACACC AATCTGTTCA TGTTCTGTTC TTGCTGAGCT GTTCTCTTCG ATGACAACAA CATTACATTT TTGGTTCTTT 22140
22,160 22,180 22,200 22,220
AU  TCCTTTTCTT GTTCAGCGTC AGCTTCCTTT ATATTTGTTT CTTCGGTTTC TGCTGTATAT GATGCTGAGT TTTCTTCTTT GGTATCGTTT 22212
Ref TCCTTTTCTT GTTCAGCGTC AGCTTCCTTT ATATTTGTTT CTTCGGTTTC TGCTGTATAT GATGCTGAGT TTTCTTCTTT GGTATCGTTT 22230
22,240 22,260 22,280 22,300 22,320
AU  TTGGACTCCC CAAGCTCAAC TGAGGAACTA GGGACATGAT CTTCTTTCAC TTCTATTGGT GCTATTTCTT CTTTTGACTC CTGTATAATA 22302
Ref TTGGACTCCC CAAGCTCAAC TGAGGAACTA GGGACATGAT CTTCTTTCAC TTCTATTGGT GCTATTTCTT CTTTTGACTC CTGTATAATA 22320
22,340 22,360 22,380 22,400
AU  TCAAACAGAC TAGAATTATT ATTTGGTTTT TCTGTTTCCT CTATGGACAC CGATTCCTCG ATGATTGAGT TGCTAGTCTT TAAAAAAATT 22392
Ref TCAAACAGAC TAGAATTATT ATTTGGTTTT TCTGTTTCCT CTATGGACAC CGATTCCTCG ATGATTGAGT TGCTAGTCTT TAAAAAAATT 22410
22,420 22,440 22,460 22,480 22,500
AU  GAATATATAT GTTGATGGAA GATTGAGTTC AGAACAGTTG AGCTATTAGT GAGTCATACA ATCTACTTAC CTCTGTTATC GAAACCACTG 22482
Ref GAATATATAT GTTGATGGAA GATTGAGTTC AGAACAGTTG AGCTATTAGT GAGTCATACA ATCTACTTAC CTCTGTTATC GAAACCACTG 22500
```

22,520 22,540 22,560 22,580

AU CTGCAGTATT CTCTTCTTTA GCTTTTTCAT CCATCGTCTC GGCCATTAAT GTTGTCTGAT CCTTCTTGTT ATCTTCCTCC TCTAGAATTT 22572

Ref CTGCAGTATT CTCTTCTTTA GCTTTTTCAT CCATCGTCTC GGCCATTAAT GTTGTCTGAT CCTTCTTGTT ATCTTCCTCC TCTAGAATTT 22590

22,600 22,620 22,640 22,660 22,680

AU GTTTTTCGGA ATTCTCAACT GTGTTGTCTG GTTCCAGCTT TTCACAGACC TGAACAAAAA ATAATATCAA AAGTTCGGTG AAACATGGAG 22662

Ref GTTTTTCGGA ATTCTCAACT GTGTTGTCTG GTTCCAGCTT TTCACAGACC TGAACAAAAA ATAATATCAA AAGTTCGGTG AAACATGGAG 22680

22,700 22,720 22,740 22,760

AU GTTTTCAGCT TTCAACTTGA TGTTGAAAAA AAATAAAAAC TTCATCTTAA GAACTAGGCT CTTCAAAATT TTAGAAGTAA CATGAATTTC 22752

Ref GTTTTCAGCT TTCAACTTGA TGTTGAAAAA AAATAAAAAC TTCATCTTAA GAACTAGGCT CTTCAAAATT TTAGAAGTAA CATGAATTTC 22770

22,780 22,800 22,820 22,840 22,860

AU ACCTCTACTT CAGGTGTGGC TTCATTTTCA TTTTCTTCTG TAGCTTTCAG TGAGTCATTG ATGATAGCAC ATTCTTCCTT CACAATGAAT 22842

Ref ACCTCTACTT CAGGTGTGGC TTCATTTTCA TTTTCTTCTG TAGCTTTCAG TGAGTCATTG ATGATAGCAC ATTCTTCCTT CACAATGAAT 22860

22,880 22,900 22,920 22,940

AU GTTGAGCCTT CATGAACCTG CATGAAGAAC ATGGTGTACT TTTTTTAACA ATTACAGTGC TAATCTTGGA GAAGTGTTTA AAATTGTTTT 22932

Ref GTTGAGCCTT CATGAACCTG CATGAAGAAC ATGGTGTACT TTTTTTAACA ATTACAGTGC TAATCTTGGA GAAGTGTTTA AAATTGTTTT 22950

22,960 22,980 23,000 23,020 23,040

AU AAAAGAAGCT TGAAACTGAG TTAGTATTCA TTAGTGCTTT AAATCCAGAC CTCTTTGGTT TCTTCCTGAT TTAAGTTATC TTCTACGTTC 23022

Ref AAAAGAAGCT TGAAACTGAG TTAGTATTCA TTAGTGCTTT AAATCCAGAC CTCTTTGGTT TCTTCCTGAT TTAAGTTATC TTCTACGTTC 23040

23,060 23,080 23,100 23,120

AU TCCTTGGCAT CCTTTGGTAG TGCACTTCTG TTCTCTGACT CCTGCTGTAT ACTCTCTTCT TTGTGTTCTA AAGCTTCATT TTCCACTGGT 23112

Ref TCCTTGGCAT CCTTTGGTAG TGCACTTCTG TTCTCTGACT CCTGCTGTAT ACTCTCTTCT TTGTGTTCTA AAGCTTCATT TTCCACTGGT 23130

23,140 23,160 23,180 23,200 23,220

AU TCAACATCGG AGAACTTGTA CTGCTTTTCG TGCTCATTGT CCATTAGTTG TCCAACGAAT GAGTCAGTAA CATGTAAATC ATCAGCATTC 23202

Ref TCAACATCGG AGAACTTGTA CTGCTTTTCG TGCTCATTGT CCATTAGTTG TCCAACGAAT GAGTCAGTAA CATGTAAATC ATCAGCATTC 23220

23,240 23,260 23,280 23,300

AU TCGTTTGAAA TCTCAACTTC CACGGTTTCA TTACTCTGAC TTGCTTTAGT TTGTAGGTGG TCAGTTGGTG AGTCTTCCCT CGTTTGTCCC 23292

Ref TCGTTTGAAA TCTCAACTTC CACGGTTTCA TTACTCTGAC TTGCTTTAGT TTGTAGGTGG TCAGTTGGTG AGTCTTCCCT CGTTTGTCCC 23310

23,320 23,340 23,360 23,380 23,400

AU TCGGATCTCT TGTCATGGTC TTTGACTGGT GTTGCTTCTT CAATGCCTGT GCTTGTTTCT GCAGTTTCGG CACAGATGGC AGTATTGACA 23382

Ref TCGGATCTCT TGTCATGGTC TTTGACTGGT GTTGCTTCTT CAATGCCTGT GCTTGTTTCT GCAGTTTCGG CACAGATGGC AGTATTGACA 23400

23,420 23,440 23,460 23,480

AU TCCTCAGAAA CTGCATTCCA AGGACATGCA CGGTTCTCCT TTTCGGGTTC ATTCTTTTGC TTGAAGCTCT CCTCTAGGAT TTCGTCCTTC 23472

Ref TCCTCAGAAA CTGCATTCCA AGGACATGCA CGGTTCTCCT TTTCGGGTTC ATTCTTTTGC TTGAAGCTCT CCTCTAGGAT TTCGTCCTTC 23490

23,500 23,520 23,540 23,560 23,580

AU TTGGAATTTC CCTCCTGATT CTTCTGTGCG AGACCAATAT TTTCTACTTC ACTAATTTCT CCTTCAGGCA TTGGAACAAT GGATTCTTTG 23562

Ref TTGGAATTTC CCTCCTGATT CTTCTGTGCG AGACCAATAT TTTCTACTTC ACTAATTTCT CCTTCAGGCA TTGGAACAAT GGATTCTTTG 23580

23,600 23,620 23,640 23,660

AU TCCTTTCTCA TTTCTTCCGA TCCCTTTGTT ATAGCGAGCC CCTCGCCTGT CACCTGTACA ACTTTCACAT CCACCACAAC ATTTTCTGAG 23652

Ref TCCTTTCTCA TTTCTTCCGA TCCCTTTGTT ATAGCGAGCC CCTCGCCTGT CACCTGTACA ACTTTCACAT CCACCACAAC ATTTTCTGAG 23670

23,680 23,700 23,720 23,740 23,760

AU TGCTTTTGTT GAGCTTGATC TTCTTCAGAA GTTTCATCAA CTAATTTCCT CACTTCTAAG AGAGTTGCAT CGTTTTCTTC TGAATGCATA 23742

Ref TGCTTTTGTT GAGCTTGATC TTCTTCAGAA GTTTCATCAA CTAATTTCCT CACTTCTAAG AGAGTTGCAT CGTTTTCTTC TGAATGCATA 23760

23,780 23,800 23,820 23,840

AU GCAGAATGTT CCTTAACATC ATCTCCTAAT ATATCTTTCA ACAGAATCTT GTCGGTTGTC ATTTCCATTT GATTTTCACT GCCTGCATTC 23832

Ref GCAGAATGTT CCTTAACATC ATCTCCTAAT ATATCTTTCA ACAGAATCTT GTCGGTTGTC ATTTCCATTT GATTTTCACT GCCTGCATTC 23850

23,860 23,880 23,900 23,920 23,940

AU AGAATTTTAT CCTGCATTCT ATCTTCTTCT TGGGGAATAT CTCTGTCCTT CTCACCTGTC ATGCACGATT CTGCTGATTT TGCATCAATG 23922

Ref AGAATTTTAT CCTGCATTCT ATCTTCTTCT TGGGGAATAT CTCTGTCCTT CTCACCTGTC ATGCACGATT CTGCTGATTT TGCATCAATG 23940

23,960 23,980 24,000 24,020

AU TCAGCACTTC CTAAACCATT CCCATGATCT TCACCATCAA CGACATTATT TATTTCATTC TTTTTCTCGT CTTCTTCTGC TTCGGCTTTA 24012

Ref TCAGCACTTC CTAAACCATT CCCATGATCT TCACCATCAA CGACATTATT TATTTCATTC TTTTTCTCGT CTTCTTCTGC TTCGGCTTTA 24030

24,040 24,060 24,080 24,100 24,120

AU GCTTCCTTTA AGTTTGTTTG TAACATTTCC GCTGCTTGTG AAGTGCAGTC CAAATCAGCA GTATCAATAT GTTCTTTTAC TGCTGTTGCT 24102

Ref GCTTCCTTTA AGTTTGTTTG TAACATTTCC GCTGCTTGTG AAGTGCAGTC CAAATCAGCA GTATCAATAT GTTCTTTTAC TGCTGTTGCT 24120

24,140 24,160 24,180 24,200

AU TCTTCAGTAT CCATTTTAGA CTCCTTGGGC TCAAACGAAG AGCTGCAGAC AAGTTCTTCT TTGACTTCGA TTAGAGTTAT TTCTTCTTTT 24192

Ref TCTTCAGTAT CCATTTTAGA CTCCTTGGGC TCAAACGAAG AGCTGCAGAC AAGTTCTTCT TTGACTTCGA TTAGAGTTAT TTCTTCTTTT 24210

24,220 24,240 24,260 24,280 24,300

AU GCTGTACCCA AAGCTTCCGT TTCTGATCCT TCACATGCCT TCACGATAAA CAAAATTTCA ATATAAACAT GATAAAGTTC CAGATGGTTA 24282

Ref GCTGTACCCA AAGCTTCCGT TTCTGATCCT TCACATGCCT TCACGATAAA CAAAATTTCA ATATAAACAT GATAAAGTTC CAGATGGTTA 24300

24,320 24,340 24,360 24,380

AU CAGACTATTA TAAGACAAAT TCCAACAGGT CACGGGTCAT CTTAGTGCTA AGTTAATGAA CTGAATTTTT TATCCTTTGA AATCCCAGTT 24372

Ref CAGACTATTA TAAGACAAAT TCCAACAGGT CACGGGTCAT CTTAGTGCTA AGTTAATGAA CTGAATTTTT TATCCTTTGA AATCCCAGTT 24390

24,400 24,420 24,440 24,460 24,480

AU ATCTTGTGGT TTGCTTGCTG GCATCAGTTG CTTCATGACA TCTCCTATTG TGGAACTCTT TTTTTGGCCT TCTCTTTTCT GAATTTGATC 24462

Ref ATCTTGTGGT TTGCTTGCTG GCATCAGTTG CTTCATGACA TCTCCTATTG TGGAACTCTT TTTTTGGCCT TCTCTTTTCT GAATTTGATC 24480

24,500 24,520 24,540 24,560

AU TGAATTTACT CGGCTCTATT ACTTTTACAC TGTTTAATGG ATAGTCTTTC GATGGGGCAT AAGACTTCTG GTTGTGCTGT TTTGCATTGT 24552

Ref TGAATTTACT CGGCTCTATT ACTTTTACAC TGTTTAATGG ATAGTCTTTC GATGGGGCAT AAGACTTCTG GTTGTGCTGT TTTGCATTGT 24570

24,580 24,600 24,620 24,640 24,660

AU ATGCATGTGT ATGCCAAAGA TTTTTATTTG TTCCATGTCT ATTGATGGAA GTTCGAGTTC AGAAATATAT TATAAGTTTG AAGTTTGAAG 24642

Ref ATGCATGTGT ATGCCAAAGA TTTTTATTTG TTCCATGTCT ATTGATGGAA GTTCGAGTTC AGAAATATAT TATAAGTTTG AAGTTTGAAG 24660

24,680 24,700 24,720 24,740

AU ATTAGAGAGT CATATAATTT TCTTACCTCT TTAAGTGTAA TCTGATCATT GTTGGTGTCT TCCTCTAGAA TTTGTTTTCC ATCATTCTCA 24732

Ref ATTAGAGAGT CATATAATTT TCTTACCTCT TTAAGTGTAA TCTGATCATT GTTGGTGTCT TCCTCTAGAA TTTGTTTTCC ATCATTCTCA 24750

```
24,760 24,780 24,800 24,820 24,840
AU  ACTGTGTTGT CCAGTTCCAG CTTTTCAGTG ACCTGATTGA AAATATATTG AGAGATTGGT TAAAAATGGA GCTTTCAGAT TTTAACAAAA 24822
Ref ACTGTGTTGT CCAGTTCCAG CTTTTCAGTG ACCTGATTGA AAATATATTG AGAGATTGGT TAAAAATGGA GCTTTCAGAT TTTAACAAAA 24840
24,860 24,880 24,900 24,920
AU  AAATTGAAAT TGAGTTAGTA TTATGATCTA GAATCAGACC TCTTTAGTTT CTTCGTGTTT CAAGTTATCT TCTATGTGAT CATTTAGCTC 24912
Ref AAATTGAAAT TGAGTTAGTA TTATGATCTA GAATCAGACC TCTTTAGTTT CTTCGTGTTT CAAGTTATCT TCTATGTGAT CATTTAGCTC 24930
24,940 24,960 24,980 25,000 25,020
AU  TTGCTGTATC CCCTTGTGTT TGTTGTTTAA AGCTTCACTG CACTCTGAGC CATTTCCGGA GTTCAAGTGA TATGCCTCTT TTATAAACAT 25002
Ref TTGCTGTATC CCCTTGTGTT TGTTGTTTAA AGCTTCACTG CACTCTGAGC CATTTCCGGA GTTCAAGTGA TATGCCTCTT TTATAAACAT 25020
25,040 25,060 25,080 25,100
AU  TGTGTTTTCA TTGCCCATTT CACTTGTTTC AGATTTTAAA TCTGTTGCTG CTGTTTCTGT GAATATGTGC TCTGGAATTG TGTAATCACT 25092
Ref TGTGTTTTCA TTGCCCATTT CACTTGTTTC AGATTTTAAA TCTGTTGCTG CTGTTTCTGT GAATATGTGC TCTGGAATTG TGTAATCACT 25110
25,120 25,140 25,160 25,180 25,200
AU  GCTTTGACTT GCTTTAGAGG CATCTTCACT AACTTTTTCC TTCTCCTTGA CTGCAACCAT TGCATCATTG AATTGTTCAT TGGGTTGAAC 25182
Ref GCTTTGACTT GCTTTAGAGG CATCTTCACT AACTTTTTCC TTCTCCTTGA CTGCAACCAT TGCATCATTG AATTGTTCAT TGGGTTGAAC 25200
25,220 25,240 25,260 25,280
AU  ATCGGAGAAC TCAAACTGCT TTTCATACTC GTTTTCAGCT GATTGTTGAA CTGATGAATC AGTAACATGT AAATCATCAG CATTGTCATT 25272
Ref ATCGGAGAAC TCAAACTGCT TTTCATACTC GTTTTCAGCT GATTGTTGAA CTGATGAATC AGTAACATGT AAATCATCAG CATTGTCATT 25290
25,300 25,320 25,340 25,360 25,380
AU  TGAAATCTCA AGTTCCATAG TTTTGGTATT CTGGTTTACT TCATTTTTTT GGTGTTTGGT TGATGAGTCT TCCCTCCCTT CTCTCTCGGA 25362
Ref TGAAATCTCA AGTTCCATAG TTTTGGTATT CTGGTTTACT TCATTTTTTT GGTGTTTGGT TGATGAGTCT TCCCTCCCTT CTCTCTCGGA 25380
25,400 25,420 25,440 25,460
AU  TCTTTCTTCA TGGTTTTCGA CTGATGTTTT TTCTCTGATG CTTGTGCTTG TTTCTGTAGT TTCACGAGAG ACGGCAGTAT TGACATCCTC 25452
Ref TCTTTCTTCA TGGTTTTCGA CTGATGTTTT TTCTCTGATG CTTGTGCTTG TTTCTGTAGT TTCACGAGAG ACGGCAGTAT TGACATCCTC 25470
25,480 25,500 25,520 25,540 25,560
AU  ATGAACTACA TTCAGGGGAC ATGAAGGCAT CTCCTTTTCG GGTTCATTTT TCTGCTGGAT TGGGATCTCT TCCTCCGGTT TTCCTTCAGG 25542
Ref ATGAACTACA TTCAGGGGAC ATGAAGGCAT CTCCTTTTCG GGTTCATTTT TCTGCTGGAT TGGGATCTCT TCCTCCGGTT TTCCTTCAGG 25560
25,580 25,600 25,620 25,640
AU  CATAAGAACA GTGGATTCCT CTTCCCTTTT CATTTCATCT AATGCCCCAG TTACTGCCGG CTTTTTGTCT GTGTCTGGTA CAACTTCCAT 25632
Ref CATAAGAACA GTGGATTCCT CTTCCCTTTT CATTTCATCT AATGCCCCAG TTACTGCCGG CTTTTTGTCT GTGTCTGGTA CAACTTCCAT 25650
25,660 25,680 25,700 25,720 25,740
AU  GTCCACTGCA ATCTTTTCTA AGTGCTTATA CGCCCTATCT TTTTCCAAAG TTTCATCTGT TATTTTCCTT TCTTCAGTGG TTTCGTATTC 25722
Ref GTCCACTGCA ATCTTTTCTA AGTGCTTATA CGCCCTATCT TTTTCCAAAG TTTCATCTGT TATTTTCCTT TCTTCAGTGG TTTCGTATTC 25740
25,760 25,780 25,800 25,820
AU  TTCTGAAGGA ATGGTAGAAT GTTCCTTAAC TTCATCTCCT ATTGCTTCTT TCAAGGGAAC ATCCTCAGTG GTCATCTCCA TTCTATCTTC 25812
Ref TTCTGAAGGA ATGGTAGAAT GTTCCTTAAC TTCATCTCCT ATTGCTTCTT TCAAGGGAAC ATCCTCAGTG GTCATCTCCA TTCTATCTTC 25830
25,840 25,860 25,880 25,900 25,920
AU  ACTTACTGCA TTCGGAATTT TATCCTGCAT TCCATCTTCT TCTTGGGGTT TGGGAGCCTC CACGTTCTTC TCGCATGTTA CAGAGAAGTC 25902
Ref ACTTACTGCA TTCGGAATTT TATCCTGCAT TCCATCTTCT TCTTGGGGTT TGGGAGCCTC CACGTTCTTC TCGCATGTTA CAGAGAAGTC 25920
25,940 25,960 25,980 26,000
AU  CATTGAATTC ACATCAATGT TAGCACCTTC TACACTGACC ATGTCATGTT CAGTTGTGGC TAGGCCAATC TCTTTGACCG CAACAACATT 25992
Ref CATTGAATTC ACATCAATGT TAGCACCTTC TACACTGACC ATGTCATGTT CAGTTGTGGC TAGGCCAATC TCTTTGACCG CAACAACATT 26010
26,020 26,040 26,060 26,080 26,100
AU  CTTTTCCCCA TCTGCTTCAT CTTCTTTTAC TGATGTTTCT TCTTCAGTGT CCATTCTGGA TTCCTTGAGC TCAAACGACG AGCCACGAAC 26082
Ref CTTTTCCCCA TCTGCTTCAT CTTCTTTTAC TGATGTTTCT TCTTCAGTGT CCATTCTGGA TTCCTTGAGC TCAAACGACG AGCCACGAAC 26100
26,120 26,140 26,160 26,180
AU  AAGGTCCTCT TCGAGGGTTT GTTGTATTTC TTCTTTTGAC TCCTTTTCAT CAAATGGACT TGGATCATTA TCTCGCTTTG CTCTTTGCTC 26172
Ref AAGGTCCTCT TCGAGGGTTT GTTGTATTTC TTCTTTTGAC TCCTTTTCAT CAAATGGACT TGGATCATTA TCTCGCTTTG CTCTTTGCTC 26190
26,200 26,220 26,240 26,260 26,280
AU  TATAGAGTCC ATTTCCGTGA CCTTCGAGTT GGTAGCCTGT ATAACAATTG AGCGGGTAAG TTAACAACAA ATAATTGTAC GAAAGGAAAA 26262
Ref TATAGAGTCC ATTTCCGTGA CCTTCGAGTT GGTAGCCTGT ATAACAATTG AGCGGGTAAG TTAACAACAA ATAATTGTAC GAAAGGAAAA 26280
26,300 26,320 26,340 26,360
AU  GAATTGTTGG ACCTAAATTA ACTATCATTG TTAGCTGACC TCTTCTGTCA ACTTCATTAG GCTGCTTTCA ACTTGCTTCA TGTTTTCCTC 26352
Ref GAATTGTTGG ACCTAAATTA ACTATCATTG TTAGCTGACC TCTTCTGTCA ACTTCATTAG GCTGCTTTCA ACTTGCTTCA TGTTTTCCTC 26370
26,380 26,400 26,420 26,440 26,460
AU  TGCAATTTCA TCTGTAATTG ACGAATCAGA GATGTCAGGT ATTGGATGGT TCTTTGTTTC CGGATTTTCT TTTCCGGTAC CTGGAGCCTC 26442
Ref TGCAATTTCA TCTGTAATTG ACGAATCAGA GATGTCAGGT ATTGGATGGT TCTTTGTTTC CGGATTTTCT TTTCCGGTAC CTGGAGCCTC 26460
26,480 26,500 26,520 26,540
AU  CATTTCTGAT TCTTCATGGA CCTGCATGAA AAAACACAAT TTTGATTTAA CTACGACAAA TTACTAGAAG GTAACAGATC AGATTATAAA 26532
Ref CATTTCTGAT TCTTCATGGA CCTGCATGAA AAAACACAAT TTTGATTTAA CTACGACAAA TTACTAGAAG GTAACAGATC AGATTATAAA 26550
26,560 26,580 26,600 26,620 26,640
AU  CAAGAACAAA CTTGAAGTAG GATAAAGCAA AAAAACTGTT TAAATGCAGA GTGTGGTACA AGATGGTCCG GGTCTGCTGT TCTGCATGTT 26622
Ref CAAGAACAAA CTTGAAGTAG GATAAAGCAA AAAAACTGTT TAAATGCAGA GTGTGGTACA AGATGGTCCG GGTCTGCTGT TCTGCATGTT 26640
26,660 26,680 26,700 26,720
AU  GTGCATGTGT GTGGATAATG ATTGTTTTTG TCATGCCTTT CGTGGAAGTT CGAGTTTGGA TATACTGAAT AAGTGAGAAG TTCAGAGATC 26712
Ref GTGCATGTGT GTGGATAATG ATTGTTTTTG TCATGCCTTT CGTGGAAGTT CGAGTTTGGA TATACTGAAT AAGTGAGAAG TTCAGAGATC 26730
26,740 26,760 26,780 26,800 26,820
AU  AGTATGTCAT ATAATCTACT TACCTCTTTA ACAGACATCG TTTCCCTCCC ATCTCCCTCA GATCTGTCTT CATGGTCTTT GACAGATGTT 26802
Ref AGTATGTCAT ATAATCTACT TACCTCTTTA ACAGACATCG TTTCCCTCCC ATCTCCCTCA GATCTGTCTT CATGGTCTTT GACAGATGTT 26820
26,840 26,860 26,880 26,900
AU  GCCTCTTCGA TGCTTGTGCT TGTTTTTGCA GTTTCACCAG AGATGTCAAT ATTTGCATCT TTGTAAACTG CATTCATAGG ATTTGCAGGC 26892
Ref GCCTCTTCGA TGCTTGTGCT TGTTTTTGCA GTTTCACCAG AGATGTCAAT ATTTGCATCT TTGTAAACTG CATTCATAGG ATTTGCAGGC 26910
26,920 26,940 26,960 26,980 27,000
AU  TTCTCATTTG GGGTTGAAAC ATGTTCTGCT TCAGTAACGT ATTCTTTAGG CATTGCAATT GCGGATTCCT CATCTATTTC CATTTCATCT 26982
Ref TTCTCATTTG GGGTTGAAAC ATGTTCTGCT TCAGTAACGT ATTCTTTAGG CATTGCAATT GCGGATTCCT CATCTATTTC CATTTCATCT 27000
```

```
                                27,020                        27,040                        27,060                        27,080
AU  GATGCCTCCG TCACGGCCAG CTCCTTGTCT GCTGCCTGTA TTGCGTCCAC TGCAACCTTT TCAGATTCAG AGTTTGCATC ATCCTTACAT 27072
Ref GATGCCTCCG TCACGGCCAG CTCCTTGTCT GCTGCCTGTA TTGCGTCCAC TGCAACCTTT TCAGATTCAG AGTTTGCATC ATCCTTACAT 27090
                 27,100                        27,120                        27,140                        27,160                        27,180
AU  AATTCATTTG ACGTGCTGAA ATCTTGGTCA GTTTTCTCAT CAATCTCACA TTGTCCAGTT TCTTCTCTTG TACACTCTTC AACAATATTC 27162
Ref AATTCATTTG ACGTGCTGAA ATCTTGGTCA GTTTTCTCAT CAATCTCACA TTGTCCAGTT TCTTCTCTTG TACACTCTTC AACAATATTC 27180
                                27,200                        27,220                        27,240                        27,260
AU  TCCATCCTGA TATCAGAGGC TTGAGAATGC AGCTCTTTGG TTTTCTTGAT GGAAGAAGTA TCGAACAAAT TCTCATCTTG GCTGGCATCA 27252
Ref TCCATCCTGA TATCAGAGGC TTGAGAATGC AGCTCTTTGG TTTTCTTGAT GGAAGAAGTA TCGAACAAAT TCTCATCTTG GCTGGCATCA 27270
                 27,280                        27,300                        27,320                        27,340                        27,360
AU  TTTTCTCCAT TGACGCATTT GTTCTCTTCA GGTTCATGTA CGCAAGCAAC CTCTTCACTT TCAGCTACCA TTGAGGGTAA GTTGTTCACT 27342
Ref TTTTCTCCAT TGACGCATTT GTTCTCTTCA GGTTCATGTA CGCAAGCAAC CTCTTCACTT TCAGCTACCA TTGAGGGTAA GTTGTTCACT 27360
                                27,380                        27,400                        27,420                        27,440
AU  ATTGGATTAT GAGAATCCAT TGAAGCTTGT TTCTCGAGTT CTTCACTGCC ATGATCCATG TCACCCTTTT CAATTCCGTC TTGAGTTCGG 27432
Ref ATTGGATTAT GAGAATCCAT TGAAGCTTGT TTCTCGAGTT CTTCACTGCC ATGATCCATG TCACCCTTTT CAATTCCGTC TTGAGTTCGG 27450
                 27,460                        27,480                        27,500                        27,520                        27,540
AU  TCCTCTTCTT CGATACGAAC CACTGTTTTT TCTTTGTCCT CTAGTATCTT CTCTTCTTTA AATGGAGTGC CATCCACCTC TGTGTGTTCT 27522
Ref TCCTCTTCTT CGATACGAAC CACTGTTTTT TCTTTGTCCT CTAGTATCTT CTCTTCTTTA AATGGAGTGC CATCCACCTC TGTGTGTTCT 27540
                                27,560                        27,580                        27,600                        27,620
AU  AATATTCCAC TGGTCTCATT GGCAACCATG GAAATCTCAG GTGATACTAA AAAACTTTCA CCCTTAGAGA TTCCAGTATT GCCTTCAGTG 27612
Ref AATATTCCAC TGGTCTCATT GGCAACCATG GAAATCTCAG GTGATACTAA AAAACTTTCA CCCTTAGAGA TTCCAGTATT GCCTTCAGTG 27630
                 27,640                        27,660                        27,680                        27,700                        27,720
AU  ATGATATGTG CCGTTTCGAT CCCCTTATTA TCGGCAGCTA CTTCCAAGGT CAAGTGCTTG CTCTCTGCTG TTTTCTGCAT GAAGAAAACA 27702
Ref ATGATATGTG CCGTTTCGAT CCCCTTATTA TCGGCAGCTA CTTCCAAGGT CAAGTGCTTG CTCTCTGCTG TTTTCTGCAT GAAGAAAACA 27720
                                27,740                        27,760                        27,780                        27,800
AU  AAGCTTTTAT TTTCATTTAC ATATGTGGTA TTTATTCTCT GGAAGAATAA GCTTCTTTGG TAACAAAAAA AATGGCATAC TTAAATCTAT 27792
Ref AAGCTTTTAT TTTCATTTAC ATATGTGGTA TTTATTCTCT GGAAGAATAA GCTTCTTTGG TAACAAAAAA AATGGCATAC TTAAATCTAT 27810
                 27,820                        27,840                        27,860                        27,880                        27,900
AU  TTCTGGTAAT TGTTCCCTGT CCTATGTAAT ATTAAAGCTA GTTGGCTGTC AGATAACAGT TGGCAATTTT GGACATGAAC TATAGACGTG 27882
Ref TTCTGGTAAT TGTTCCCTGT CCTATGTAAT ATTAAAGCTA GTTGGCTGTC AGATAACAGT TGGCAATTTT GGACATGAAC TATAGACGTG 27900
                                27,920                        27,940                        27,960                        27,980
AU  AAAGAATAAC GTGAACATTC TCGAGACTCG GTCTATAATG TCTCGAGACA CATTGATGAA TTTTGTTAAT TTGAATTGGA TTTGAATCTT 27972
Ref AAAGAATAAC GTGAACATTC TCGAGACTCG GTCTATAATG TCTCGAGACA CATTGATGAA TTTTGTTAAT TTGAATTGGA TTTGAATCTT 27990
                 28,000                        28,020                        28,040                        28,060                        28,080
AU  TGACTTCGAG TTTGATACTG TATAACACGG GATTTCTGTT ATGCATGACC ATATCCGTAA TTGAAAGTGG TTTGAATTAT GTCATGTCCG 28062
Ref TGACTTCGAG TTTGATACTG TATAACACGG GATTTCTGTT ATGCATGACC ATATCCGTAA TTGAAAGTGG TTTGAATTAT GTCATGTCCG 28080
                                28,100                        28,120                        28,140                        28,160
AU  AGGTGGTTGT GCAAGCATCG TGAACACAAT ACAAACATTG TCCTCTTCGG GGGTCGACTA ACCCCTCGAG GCTTTCATTC AATGGCGTGT 28152
Ref AGGTGGTTGT GCAAGCATCG TGAACACAAT ACAAACATTG TCCTCTTCGG GGGTCGACTA ACCCCTCGAG GCTTTCATTC AATGGCGTGT 28170
                 28,180                        28,200                        28,220                        28,240                        28,260
AU  CTCCTTGGAT TTGAAACCAA CACTTCCTAC AACACATATA GTAGTCTATC TTGAAAACAT AGTACCGTTT GATCTTTGCA AAAAAGACCG 28242
Ref CTCCTTGGAT TTGAAACCAA CACTTCCTAC AACACATATA GTAGTCTATC TTGAAAACAT AGTACCGTTT GATCTTTGCA AAAAAGACCG 28260
                                28,280                        28,300                        28,320                        28,340
AU  CTATATATAT GTGTTATAGG AGGTCTTAGG TTCAATCCAA CTAAGCGCAT CTTTAGGTGA GAAAGCTTTT ACGAGGGTTA GTGGACGCTC 28332
Ref CTATATATAT GTGTTATAGG AGGTCTTAGG TTCAATCCAA CTAAGCGCAT CTTTAGGTGA GAAAGCTTTT ACGAGGGTTA GTGGACGCTC 28350
                 28,360                        28,380                        28,400                        28,420                        28,440
AU  AAAGAAGACA ATTGTGCTCT CAATGCCCGT GTAACACAGC CTATGGCTGG TCAAATGGTG ATAGGTCGTG ACAATTATTT TTTAGAAAAG 28422
Ref AAAGAAGACA ATTGTGCTCT CAATGCCCGT GTAACACAGC CTATGGCTGG TCAAATGGTG ATAGGTCGTG ACAATTATTT TTTAGAAAAG 28440
                                28,460                        28,480                        28,500                        28,520
AU  AGATTTAGCT TGTCTTTAAT GTTAGTGTTA GTATCTGGTA CGGATATGTG CCCGATACGT GTTCAATTTT CTTTTTCCAA GTTTTCCTTG 28512
Ref AGATTTAGCT TGTCTTTAAT GTTAGTGTTA GTATCTGGTA CGGATATGTG CCCGATACGT GTTCAATTTT CTTTTTCCAA GTTTTCCTTG 28530
                 28,540                        28,560                        28,580                        28,600                        28,620
AU  TATTTCTAAA GAGAACTTGT TGATGCATGA ACCTTGCATC AACAGATCTA AAAATATATG CTTACTACCT TCTTTAAGCT GATTGAAAAC 28602
Ref TATTTCTAAA GAGAACTTGT TGATGCATGA ACCTTGCATC AACAGATCTA AAAATATATG CTTACTACCT TCTTTAAGCT GATTGAAAAC 28620
                                28,640                        28,660                        28,680                        28,700
AU  CGGAGATTAT GTGCTTCATC AATCAACTAG AAGGATAGTA ATAGAACTTA GAGATCCTCC AGTTTTAAGT GTTTCAACAA CACCGAAAAA 28692
Ref CGGAGATTAT GTGCTTCATC AATCAACTAG AAGGATAGTA ATAGAACTTA GAGATCCTCC AGTTTTAAGT GTTTCAACAA CACCGAAAAA 28710
                 28,720                        28,740                        28,760                        28,780                        28,800
AU  CTCAAAAGCA GAAGTGCTAT TTAAAGATGA AACCTGACGC ATGATCTTTC TGGTTTAATC TGTCATCAAA GACATCTGAT CTTTGATGAC 28782
Ref CTCAAAAGCA GAAGTGCTAT TTAAAGATGA AACCTGACGC ATGATCTTTC TGGTTTAATC TGTCATCAAA GACATCTGAT CTTTGATGAC 28800
                                28,820                        28,840                        28,860                        28,880
AU  GTAGAGTGTT CTAAAGTTTG CTTAAAGCAC AATACGTGTA AGATGAAAGA ACATTACCAC TATTGAAATG GGCTCTTGAA TGATCACTGC 28872
Ref GTAGAGTGTT CTAAAGTTTG CTTAAAGCAC AATACGTGTA AGATGAAAGA ACATTACCAC TATTGAAATG GGCTCTTGAA TGATCACTGC 28890
                 28,900                        28,920                        28,940                        28,960                        28,980
AU  TCCAGTTGCC ATTTTTTCCT GCACAAGTTA TCGAATTCTA GTTTGAGAGC CTATCATATC AAACATTCAT AAACTGTGAA TCATGTAAGA 28962
Ref TCCAGTTGCC ATTTTTTCCT GCACAAGTTA TCGAATTCTA GTTTGAGAGC CTATCATATC AAACATTCAT AAACTGTGAA TCATGTAAGA 28980
                                29,000                        29,020                        29,040                        29,060
AU  TGAAAGAACT TTACCACTAT GGAAATGGGT TCTTGAATAA TCACTGATTC AGTTGCAATT TTTTCCTGCA CAAGTGATCG AATTCGAGTT 29052
Ref TGAAAGAACT TTACCACTAT GGAAATGGGT TCTTGAATAA TCACTGATTC AGTTGCAATT TTTTCCTGCA CAAGTGATCG AATTCGAGTT 29070
                 29,080                        29,100                        29,120                        29,140                        29,160
AU  CAAGAACACT GAACAATACT GCCTATCATC CTGAAACATT CATAAACCAT GAATCATATA AGATGAAAGA ACTTTACCAC TATGGAAACC 29142
Ref CAAGAACACT GAACAATACT GCCTATCATC CTGAAACATT CATAAACCAT GAATCATATA AGATGAAAGA ACTTTACCAC TATGGAAACC 29160
                                29,180                        29,200                        29,220
AU  GGTTCTTGAA TGATCGCTGC TTCAGTTGCC ATCTACTTTT CCTGCAGATA TAATAGAATT CG 29204
Ref GGTTCTTGAA TGATCGCTGC TTCAGTTGCC ATCTACTTTT CCTGCAGATA TAATAGAATT CG 29222
```
